# Switching *N*-Alkylation Regioselectivity of Trifluoromethylated Pyrazoles Guided by Functional Group Tuning

**DOI:** 10.3390/ijms262110335

**Published:** 2025-10-23

**Authors:** Yulia O. Edilova, Yulia S. Kudyakova, Ekaterina A. Osipova, Pavel A. Slepukhin, Yanina V. Burgart, Victor I. Saloutin, Denis N. Bazhin

**Affiliations:** 1 Postovsky Institute of Organic Synthesis, Ural Branch of the Russian Academy of Sciences, Yekaterinburg 620137, Russia; 2 Department of Organic and Biomolecular Chemistry, Ural Federal University Named After the First President of Russia B.N. Eltsin, Mira Str. 19, Yekaterinburg 620002, Russia

**Keywords:** regioselectivity, alkylation, pyrazole, hydrazone, azine, carboxylate

## Abstract

The similar properties of the nitrogen atoms in azole ring complicate the regioselective *N*-functionalization of pyrazoles. This work demonstrates the role of the hydrazone substituent in the control of the alkylation selectivity of (trifluoromethyl)pyrazoles. Reaction conditions for the synthesis of 3- and 5-(trifluoromethyl)pyrazoles were developed, and all types of regioisomers formed under the alkylation of bis-pyrazolyl *NH*-ketazine were isolated. The structures of the synthesized compounds were confirmed by NMR spectroscopy and XRD data.

## 1. Introduction

**Figure 1:**
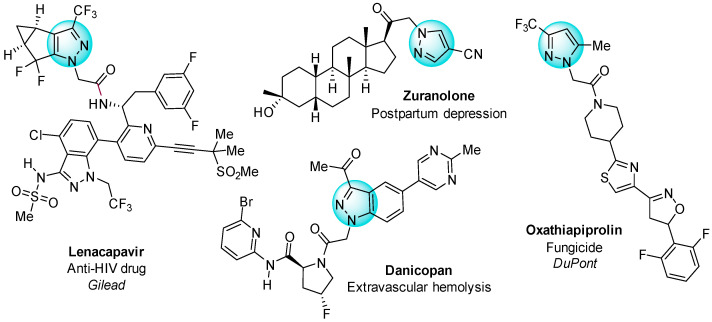
Approved pyrazole-based drugs and agrochemicals synthesized by *N*-alkylation of heterocyclic core.

Pyrazole-based compounds increasingly attract attention as promising agents in design of pharmaceuticals and agrochemicals (Figure 1) [1,2,3,4,5,6,7,8,9,10,11,12,13,14,15,16,17,18]. Many pyrazole derivatives also demonstrate significant luminescent, sensor, and catalytic properties, highlighting their versatile applications [14,19,20,21,22,23,24,25,26,27]. The tunability of the pyrazole core at four possible positions, including three carbon atoms, and one of the two nitrogen atoms makes this heterocycle a valuable tool in organic synthesis.

The diversity of pyrazole structures is achieved by heterocondensations of polyfunctional molecules, the so-called “building blocks”, to form five-membered cycles having two neighboring *N*-atoms substituted with different functional groups [14,15,19,20,21,22,23,24,25,26,27,28,29,30,31]. Another approach to substituted pyrazoles includes cycloaddition reactions with diazo compounds and unsaturated reagents. To modify the pyrazole ring, both metal-catalyzed and cross-coupling reactions are used [14].

**Figure 2:**
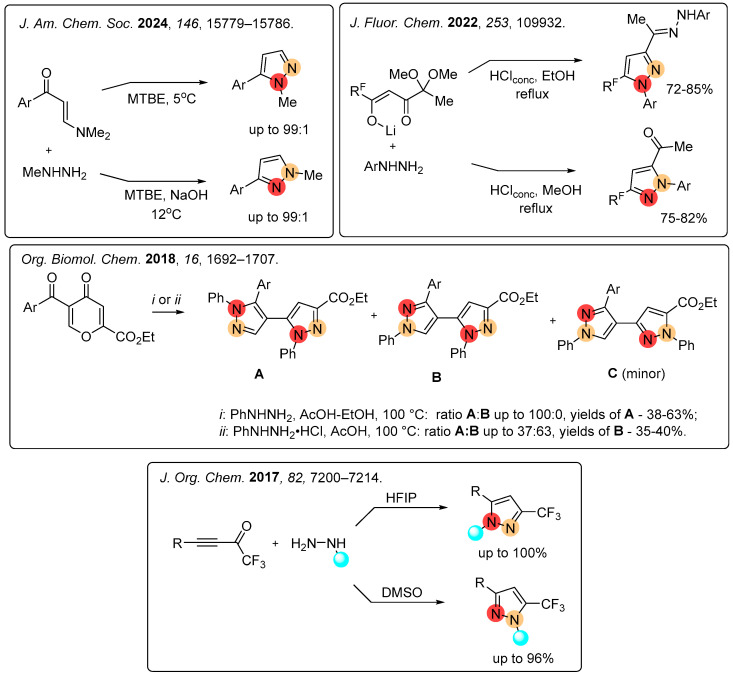
Regioselective approaches to the pyrazole core construction [33,34,35,36].

However, the regioselective synthesis of *N*-substituted pyrazoles remains a challenging issue [32,33,34,35,36,37,38,39,40,41,42,43,44,45,46,47,48,49]. The regioselectivity can be achieved by varying the β-dielectrophilic reagents, including acylacetylenes, dicarbonyl compounds, alkoxyenones, enaminoketones, and pyranones, etc. [14]. The choice of reaction conditions plays a crucial role in directing the nucleophilic attack of substituted hydrazines in reactions with enaminoketones, functionalized β-diketonates, and pyranes, leading to the formation of individual regioisomers (Figure 2) [33,34,35,36].

**Figure 3:**
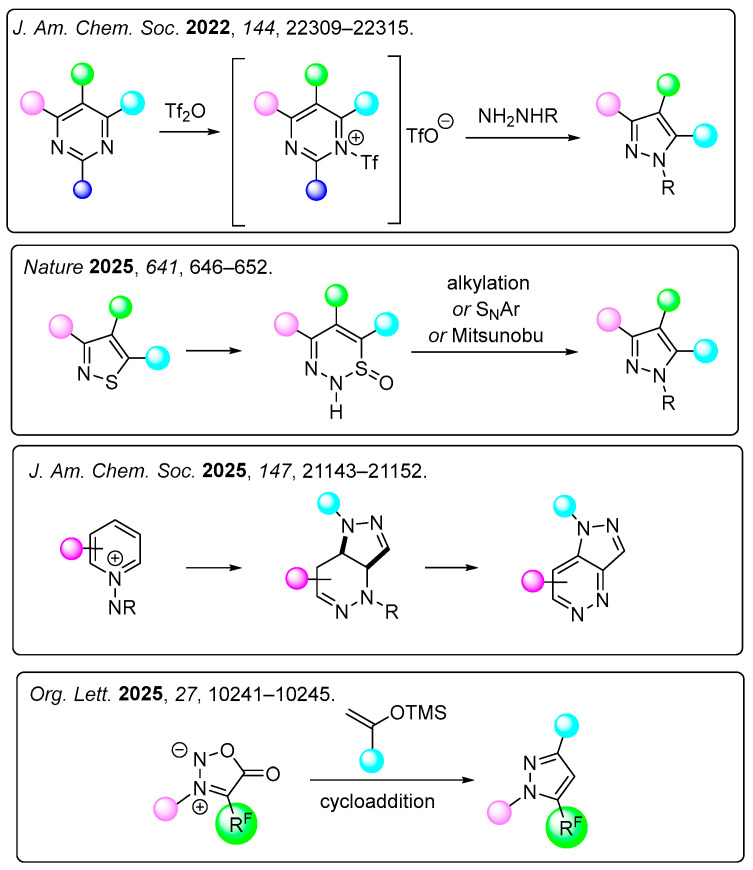
Skeletal editing in the regioselective pyrazole synthesis [39,40,41,42].

To obtain exclusively one regioisomeric pyrazole it is offered the transformations of five- or six-membered heterocycles (e.g., pyrimidines, sydnones, and thiazoles) based on skeletal editing (Figure 3) [39,40,41,42].

**Figure 4:**
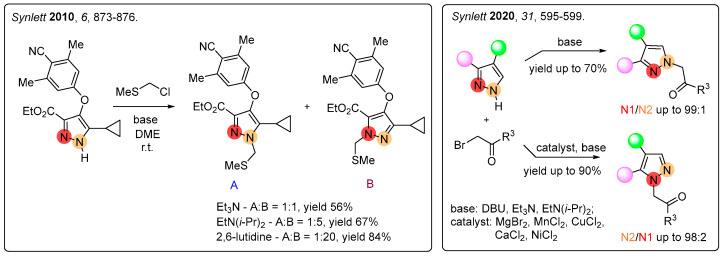
Switching pyrazole *N*-alkylation regioselectivity [37,38].

*N*-Alkylation of unsymmetric pyrazoles typically yields a mixture of regioisomers. Regioselectivity of this reaction can be controlled by the nature of the bases used or by changing the size and charge of the cation (Figure 4) [37,38].

**Figure 5:**
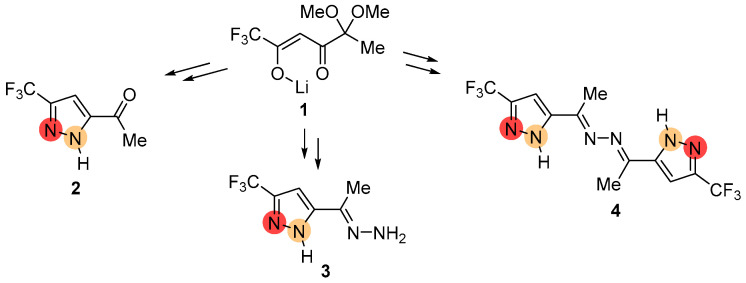
*NH*-pyrazoles derived from 1,2,4-triketone analog.

Recently we have developed the synthesis of unsymmetric pyrazoles with different functional groups, based on the condensation of hydrazines with 1,2,4-triketones and their analogs [49,50,51,52]. In particular, acetyl- and hydrazone-substituted CF_3_-pyrazoles **2**–**4** are a flexible platform for derivatization (Figure 5).

**Scheme 1:**
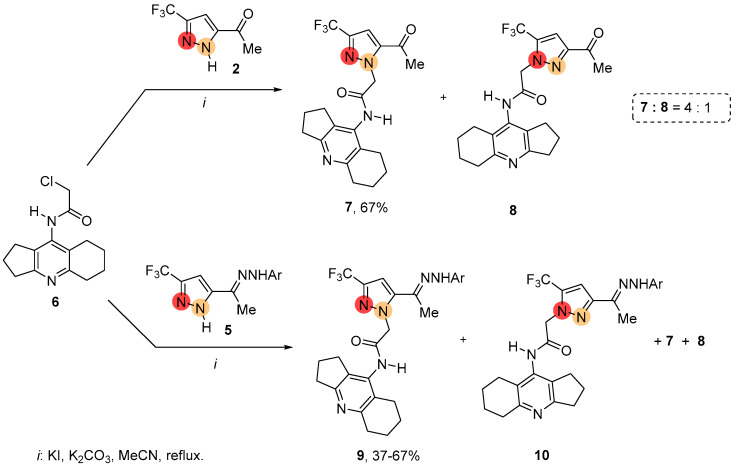
Previous results on *N*-alkylation of functionalized CF_3_-pyrazoles.

To design the bioactive molecules, acetylpyrazole **2** and its derivatives **5** were alkylated with amiridine-based chloroacetamide **6** (Scheme 1) [52]. Under standard conditions, the *N*-alkylation of acetylpyrazole **2** in the presence of potassium carbonate in acetonitrile resulted in a mixture of two possible products, **7** and **8**, with the prevalence of 3-CF_3_-regioisomer. A similar alkylation of acetylpyrazole derivatives **5** also proceeded with the formation of two possible regioisomers, **9** and **10**. However, in this case the reaction was accompanied by side processes involving the transformations of hydrazone substituents.

In this work we have investigated the alkylation of functionalized trifluoromethylated pyrazoles with ethyl iodoacetate. The attachment of a carboxylate substituent to the nitrogen atom of pyrazole is considered to be particularly valuable for the further synthesis of complex molecules in the search for biologically active compounds. On the other hand, pyrazolocarboxylates are attractive molecules as ligands for the synthesis of coordination compounds, including MOFs.

## 2. Results

### 2.1. Regioselective Synthesis of Pyrazoles

**Scheme 2:**
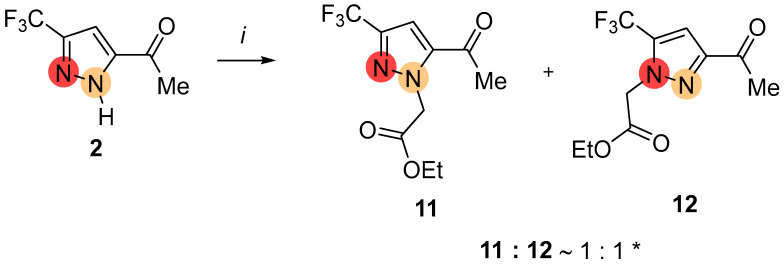
*N*-alkylation of acetyl-CF_3_-pyrazole **2**. *Reaction conditions*: *i:* ICH_2_CO_2_Et, K_2_CO_3_, MeCN, reflux. * According to ^19^F NMR data of the reaction mixture (see Section 4.3.3, Figure S1).

We initially chose CF_3_-pyrazole **2** with a 3-acetyl group, one of the available functional heterocycles based on the 1,2,4-triketone precursor, as the starting substrate for the alkylation. Pyrazole **2** reacted with iodoacetate in the presence of potassium carbonate under reflux in polar aprotic solvent to give an equimolar mixture of 3-CF_3_- and 5-CF_3_-regioisomeric pyrazoles **11** and **12** (Scheme 2). In contrast to the reported work [52], the lack of regioselectivity in *NH*-alkylation of pyrazole **2** was observed because of the functional group changing in the alkylating agent. The ratio of regioisomers was determined based on ^19^F NMR spectroscopy (see Section 2.2).

**Scheme 3:**
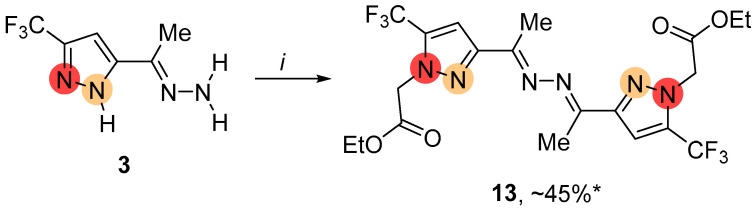
*N*-alkylation of hydrazine-substituted CF_3_-pyrazole **3**. *Reaction conditions*: *i:* ICH_2_CO_2_Et, K_2_CO_3_, MeCN, reflux. * According to ^19^F NMR data of the reaction mixture (see Section 4.3.3, Figure S2).

The alkylation of hydrazine-substituted pyrazole **3** under the same conditions primarily led to symmetric ketazine **13**, which had a substituent at the nitrogen atom adjacent to the CF_3_ group in the azole ring (Scheme 3). In addition to C-N bond formation, transamination of hydrazone fragments in pyrazole molecules took place. Along with compound **13**, the complex mixture of alkylated products was observed.

**Scheme 4:**
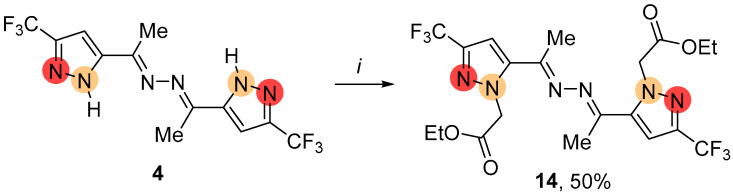
*N*-alkylation of *bis*-CF_3_-pyrazole **4**. *Reaction conditions*: *i:* ICH_2_CO_2_Et, NaH, DME–MeCN, r.t.

In contrast to a similar reaction of pyrazole **3**, the alkylation of *NH*-ketazine **4** mainly produced the bis-pyrazole **14** (Scheme 4). According to NMR and XRD data, this symmetric product had the *N*-substituent adjacent to the hydrazone moiety. The traces of mono- and bis-alkylated regioisomeric by-products were registered by GC-MS and NMR spectroscopy. Replacing K_2_CO_3_/MeCN under reflux with the NaH/DME-MeCN system at room temperature resulted in enhanced selectivity, leading to a preparative yield of 50% for ketazine **14** (see Section 4.3.2).

**Scheme 5:**
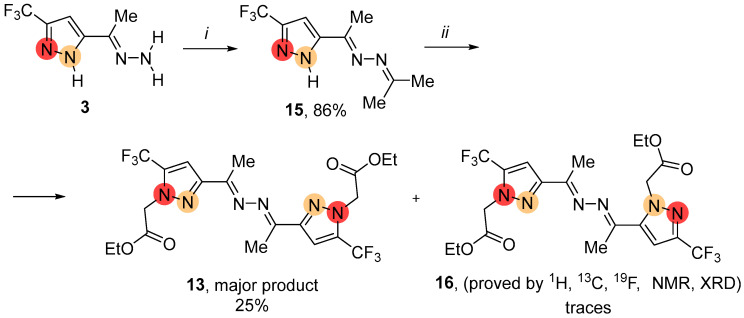
*N*-alkylation of hydrazine-substituted CF_3_-pyrazole. *Reaction conditions*: *i:* acetone, reflux; *ii:* ICH_2_CO_2_Et, K_2_CO_3_, MeCN, reflux.

The reaction of pyrazole **3** with acetone yielded the product **15** that contained an azine fragment with a structure similar to *NH*-ketazine **4** (Scheme 5). However, the preferred nucleophilic center of the pyrazole **15** was the nitrogen atom adjacent to the CF_3_ group. Furthermore, the process in this case was accompanied by both the hydrolysis and transamination of hydrazone fragments to provide symmetric 5,5′- bis(trifluoromethyl)ketazine **13** as a major product. We also observed the minor formation of unsymmetric 3,5′-bis(trifluoromethyl)ketazine **16**.

**Scheme 6:**
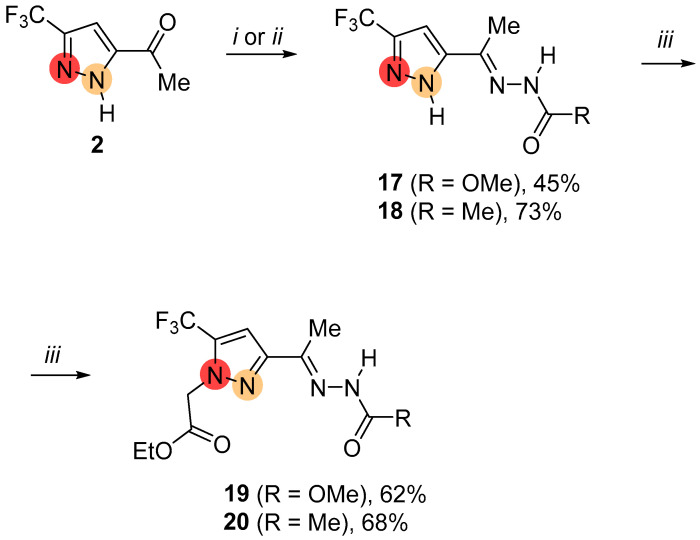
*N*-alkylation of CF_3_-pyrazoles with modified hydrazone groups. *Reaction conditions*: *i:* methyl carbazate, HCl_conc_, MeOH, reflux; *ii:* acetohydrazide, AcOH, MeCN, reflux; *iii:* ICH_2_CO_2_Et, K_2_CO_3_, MeCN, reflux.

To study the influence of the functional group on the alkylation, we synthesized the acetohydrazide- and carbazide-substituted pyrazoles **17** and **18** (Scheme 6). The further reactions of *NH*-pyrazoles **17** and **18** with ethyl iodoacetate proceeded in a highly regioselective manner, yielding exclusively the substituted 5-CF_3_-pyrazoles **19** and **20**.

**Scheme 7:**
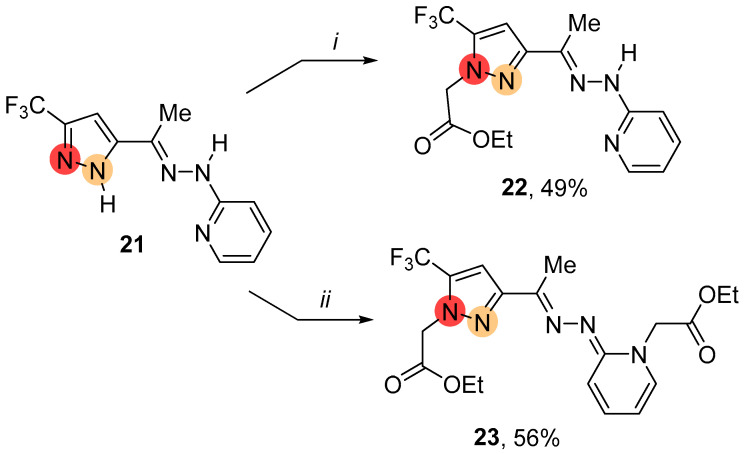
*N*-alkylation of CF_3_-pyrazole **21**. *Reaction conditions*: *i:* ICH_2_CO_2_Et (1.1 eq), NaH (1.1 eq), DME–MeCN, reflux; *ii:* ICH_2_CO_2_Et (2.2 eq), NaH (2.2 eq), DME–MeCN, reflux.

Pyridine-containing substituents are known to act as directing groups in regioselective processes [53]. The previously obtained pyrazolylhydrazone **21** with a pyridine moiety is therefore an interesting substrate for studying the alkylation [34]. It was found that using sodium hydride instead of potassium carbonate prevents the formation of regioisomeric products. As a result, the reaction of **21** with ethyl iodoacetate proceeded regioselectively at room temperature (Scheme 7). In this case, the structure of the product depended on the ratio of the starting substrate and the alkylating agent. An equimolar mixture of ethyl iodoacetate and **21** resulted in the formation of 5-regioisomeric CF_3_-pyrazole **22**. Increasing the molar ratio of ethyl iodoacetate to the substrate promoted the further alkylation of the pyridine nitrogen atom, yielding disubstituted product **23**.

**Scheme 8:**
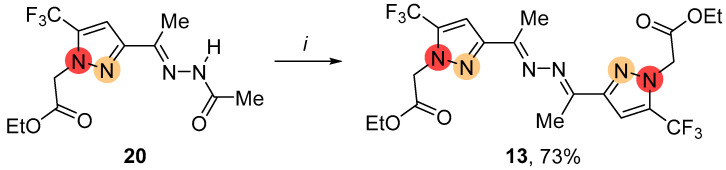
Synthesis of 5,5′-bis-CF_3_-pyrazole **13**. *Reaction conditions*: *i:* NH_2_NH_2_·2HCl, EtOH, reflux.

Next, we developed an efficient approach to the synthesis of regioisomeric 5,5′-bis-CF_3_-pyrazolylketazine **13** using the reaction of functionalized *N*-alkylated pyrazole **20** with hydrazine (Scheme 8). Pyrazole **20** reacted with hydrazine in ethanol under acid conditions to give the ketazine **13** in a high yield.

**Scheme 9:**
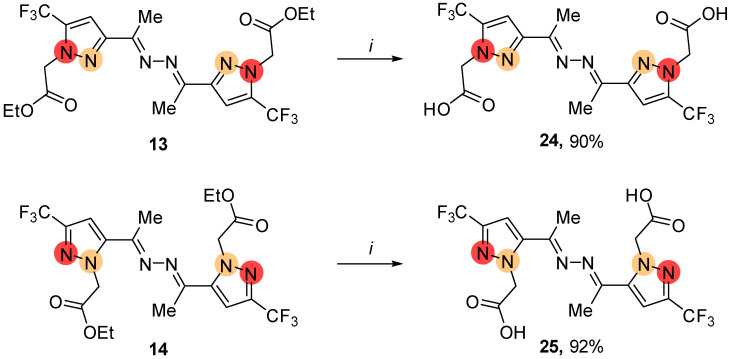
Hydrolysis of ester derivatives. *Reaction conditions*: *i:* NaOH_aq_ (10%), EtOH, reflux.

Considering the potential of carboxylate ligands for coordination chemistry, we successfully hydrolyzed the regioisomeric CO_2_Et-containing ketazines **13** and **14** into the corresponding bis-pyrazole carboxylates **24** and **25** under basic conditions (Scheme 9).

### 2.2. Regioisomeric Structure Determination

**Figure 6:**
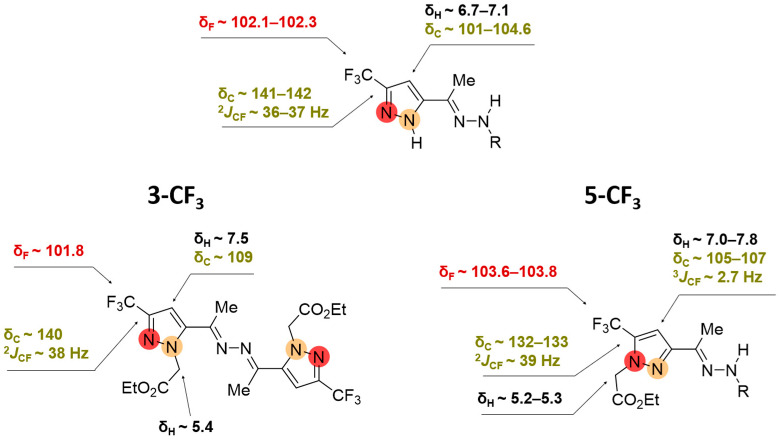
Characteristic ^1^H, ^13^C, and ^19^F NMR signals (DMSO-*d*_6_, *δ*, ppm) for starting *NH*-pyrazoles (**3**, **4**, **15**, **17**, **18**), alkylated 3-CF_3_-regioisomer (**14**) and 5-CF_3_-regioisomers (**13**, **19**, **20**, **22**).

**Figure 7:**
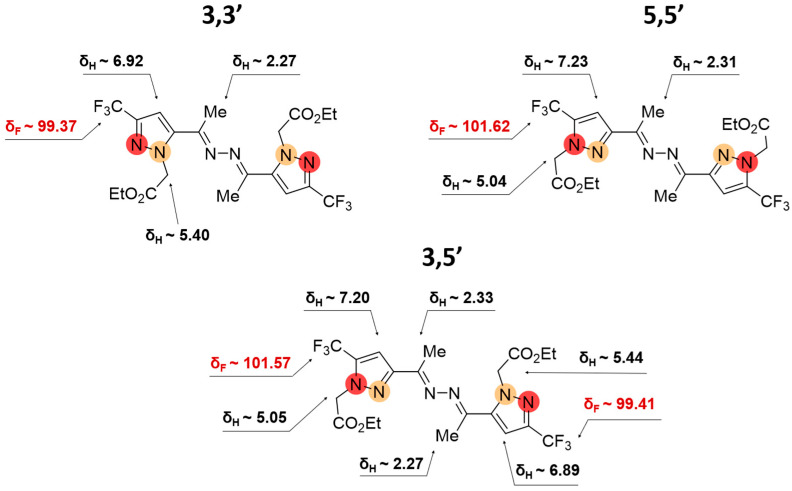
^1^H, ^19^F NMR data (CDCl_3_, *δ*, ppm) for regioisomeric bis-(trifluoromethyl)ketazines **13**, **14**, and **16**.

The structural differentiation of 3-CF_3_- and 5-CF_3_-substituted pyrazoles was readily achieved by analysis of ^19^F NMR spectra [34] (Figure 6 and Figure 7). Compared with *NH*-pyrazoles, the signal of the trifluoromethyl group of 5-CF_3_-pyrazoles was shifted downfield when substitution occurred at the adjacent nitrogen atom, whereas for 3-CF_3_-substituted derivatives, it appeared upfield in both DMSO-*d_6_* and CDCl_3_ solutions. According to ^13^C NMR data, the characteristic signals of the CCF_3_- and CH groups were observed at different δ_C_ ranges and exhibited distinct *J*_CF_ coupling constants, enabling unambiguous isomer identification (Figure 6). As for the proton signals, in the case of alkylated ketazine products, the differences were most evident in CDCl_3_ solutions (Figure 7).

**Figure 8:**
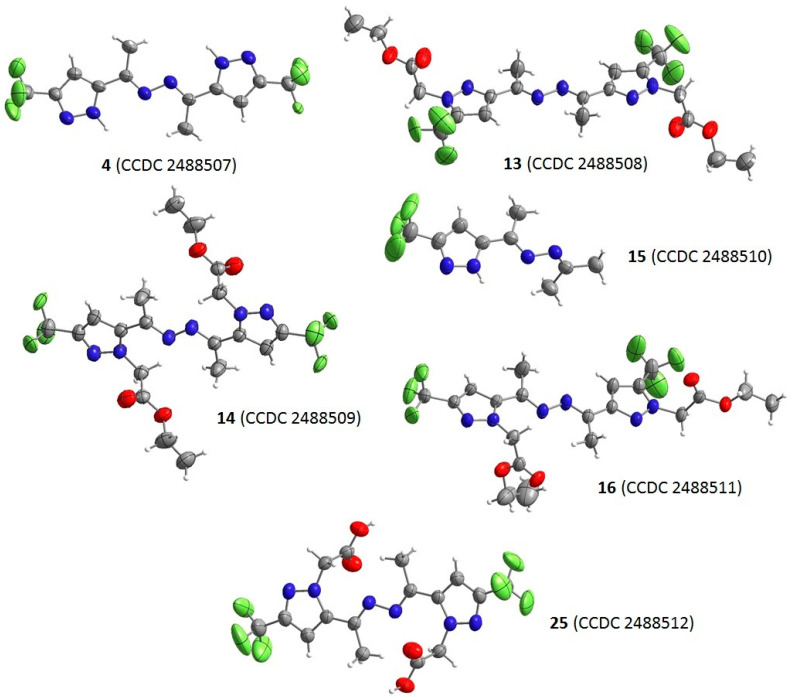
Molecular structure of pyrazoles **4**, **13**–**16**, **25** according to the XRD data.

The crystal structures of (bis)pyrazoles **4**, **13**–**16**, and **25** were confirmed by XRD data (Figure 8, Table S1). All these products have substituents located in the *trans* position relative to the diazo fragment, as expected. In most pyrazoles, the azine fragment and the azole rings were coplanar. However, in product **15**, the two imine groups lay in different planes and formed a dihedral angle of ~74° (see Figure S43). The orientation of the azole rings depended on the substituent position at the nitrogen atom of pyrazole. 3-CF_3_-Regioisomers had a common *N*,*N*-cavity involving the nitrogen atoms of the azole and hydrazone moieties, while the orientation of the azole rings and the hydrazone group in 5-CF_3_-pyrazole **13** prevented the formation of a single cavity. The structural patterns described for regioisomeric products also apply to the unsymmetric ketazine **16**. Crystal packings of pyrazoles **4**, **13**–**16**, and **25** are shown in *Supporting Information* (Figures S44–S49).

## 3. Discussion

**Scheme 10:**
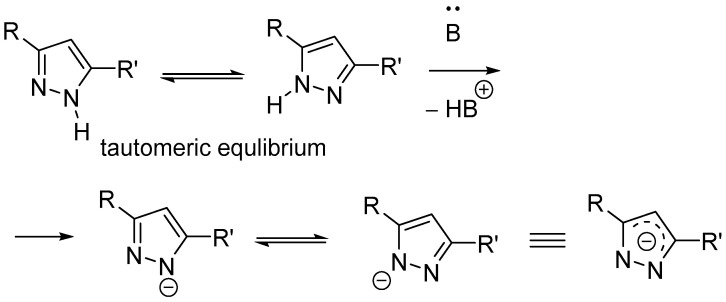
Tautomeric forms of pyrazole and resonance structures of the pyrazolate anion.

Pyrazole nitrogen atoms are known to have similar electronic properties. Therefore, *NH*-pyrazoles can exist in solution as an equilibrium mixture of two tautomers (Scheme 10). Pyrazolates are also expected to exhibit similar dual behavior due to the negative charge delocalization. This explains the lack of selectivity in some pyrazole *N*-alkylations [54].

Previous studies have shown that the selective introduction of an alkyl group at the *NH* fragment of pyrazoles is governed by both the electronic and steric properties of the substituents in the azole ring [37,38]. Alternatively, the pyrazole substituent may exert a directing effect during the reaction with the alkylating agent. This tendency became the focus of our research.

According to NMR data, initial trifluoromethylated *NH*-pyrazoles **2**–**4**, **15**, **17**, **18**, **21** exist exclusively as 3-CF_3_-regioisomers (see Section 2.2, Figure 6). This points to the influence of the CF_3_ group on the pyrazole nitrogen atoms, making them non-equivalent. In contrast, the selectivity of the reaction between pyrazoles and ethyl iodoacetate is guided by the azole functional group.

**Figure 9:**
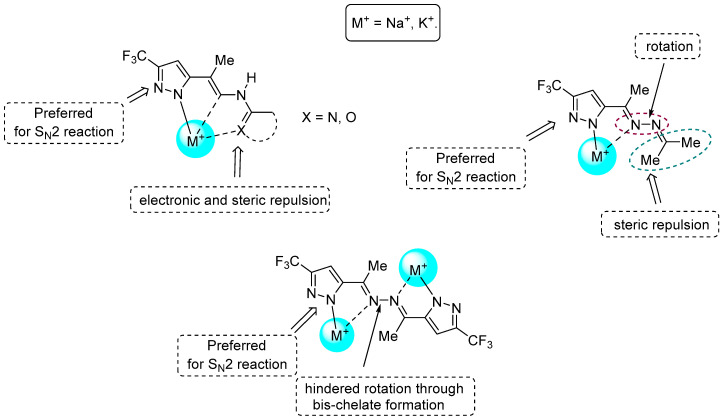
Proposed structural factors influencing on the regioselectivity of *NH*-pyrazole alkylations.

For acetylpyrazole **2**, alkylation at the *NH* group was low-selective (Scheme 2). However, conversion of the acetyl group into a hydrazone moiety enhances the regioselectivity in some cases. The functionalization of the hydrazone fragment is crucial for the synthesis of *N*-substituted 5-CF_3_-pyrazoles. We propose that the functional group is coordinated with alkali metal ions of in situ-generated pyrazolates (Figure 9). The formation of a single chelate cavity sterically hinders one of the pyrazole N-atoms, preventing it from participating in a bimolecular nucleophilic substitution with ethyl iodoacetate. In the case of *NH*-ketazine **4** and pyrazole **15**, such a coordination cavity does not appear to form; therefore, there are no steric hindrances for nucleophilic attack at the pyrazole nitrogen atom adjacent to the hydrazone fragment.

The alkylation of *NH*-ketazine **4** results in symmetric product **14** containing a substituent at the pyrazole nitrogen atom adjacent to the azine group. In compound **4**, the pyrazole substituents are presumably located in the *trans* position relative to the diazo fragment. This molecular conformation leads to alkylation of those nitrogen atoms of bis-pyrazole **4** that are distant from the CF_3_ group.

## 4. Materials and Methods

### 4.1. General Chemistry

Melting points were obtained on a Stuart SMP3 apparatus (Barloworld Scientific, Staffordshire, UK) and were uncorrected. The IR spectra were measured on a Perkin Elmer Spectrum Two instrument (Perkin Elmer, Waltham, MA, USA) equipped with a diamond crystal attenuated total reflectance (ATR) accessory in the 400–4000 cm^–1^ range. The ^1^H, ^19^F, and ^13^C NMR spectra of the synthesized compounds (see Supplementary Materials, Figures S1–S42) were recorded on a Bruker DRX-400 (400 MHz for ^1^H and 376 MHz for ^19^F) and Bruker Avance-500 spectrometers (Bruker BioSpin, Ettlingen, Germany) (500 MHz for ^1^H, 126 MHz for ^13^C, and 470 MHz for ^19^F) in DMSO-d_6_ and CDCl_3_ using SiMe_4_ (^1^H) and C_6_F_6_ (^19^F) as the internal standards. The ^13^C NMR chemical shifts were referenced to the DMSO-d_6_ signal (δ_C_ 39.5) or CDCl_3_ signal (δ_C_ 77.2). Microanalyses (C, H, N) were carried out on a Perkin Elmer PE 2400 II automatic analyzer (Perkin Elmer, Waltham, MA, USA). Mass spectra of reaction mixtures were recorded on a Shimadzu GCMSQP2020 gas chromatograph-mass spectrometer, EI ionization (70 eV). Analytical TLC (reaction progress monitoring and the products purity evaluation) was performed using ALUGRAM Xtra SIL G/UV254 plates (Macherey-Nagel GmbH & Co., Düren, Germany).

Single crystals of pyrazoles **4**, **13**–**16**, and **25** were obtained by slow evaporation from the corresponding solvent. The XRD experiments were performed on an Xcalibur 3 automated X-ray diffractometer (Oxford Diffraction, Milton Park Abingdon, Oxfordshire, UK) with a CCD detector according to standard procedure (MoKα-irradiation, graphite monochromator, ω-scans with 1° step at T = 295 (2) K). Empirical absorption correction was applied. The solution and refinement of the structures were accomplished using the OLEX2 1.2/1.3 software package [55]. The structures were solved with the SHELXT [56] structure solution program using Intrinsic Phasing and refined with the SHELXL [57] refinement package using Least Squares minimization. The X-ray diffraction data were deposited with the Cambridge Crystallographic Data Centre (CCDC no. 2488507 (**4**), 2488508 (**13**), 2488509 (**14**), 2488510 (**15**), 2488511 (**16**), 2488512 (**25**)). Copies of the data can be obtained, free of charge, on application to CCDC, 12 Union Road, Cambridge CB2 1EZ, UK [fax: +44(0)1223-336,033 or e-mail: deposit@ccdc.cam.ac.uk].

### 4.2. Reagents and Solvents

Acetylpyrazole **2** [49], hydrazonopyrazole **3**, *NH*-ketazine **4** [50], and *NH*-pyrazole **21** [34] were prepared according to previously described procedures. Other reagents were purchased from commercial sources and used as received.

### 4.3. Experimental Procedures and Characterization Data of the Synthesized Compounds

#### 4.3.1. Synthesis of *NH*-Pyrazoles **15**, **17**, **18**

*5-[1-(Propan-2-ylidenehydrazineylidene)ethyl]-3-(trifluoromethyl)-1H-pyrazole* (**15**). Hydrazonopyrazole **3** (5 mmol, 960 mg) was dissolved and refluxed in 15 mL of acetone for 4 h. The solvent was evaporated and the residue was recrystallized from Et_2_O. Yield 994 mg (86%); yellow powder; mp 105–106 °C; IR ν 3139–2857 (C–H, N–H), 1646–1627 (C=C, C=N), 1253–1122 (C–F). ^1^H NMR (400 MHz, DMSO-*d_6_*) δ 1.92 (s, 3H, Me), 2.05 (s, 3H, Me), 2.17 (s, 3H, Me), 7.14 (s, 1H, CH), 14.06 (br.s, 1H, NH); ^13^C NMR (126 MHz, DMSO-*d_6_*) δ 14.4, 18.3, 24.7, 103.9, 121.5 (q, *J* = 268.2 Hz, CF_3_), 141.2 (q, *J* = 36.0 Hz, CCF_3_), 143.0 (br.s), 148.8, 161.9; ^19^F NMR (376 MHz, DMSO-*d_6_*) δ 102.19 (s, 3F, CF_3_); Anal. calcd. for C_9_H_11_F_3_N_4_. C, 46.55; H, 4.77; N, 24.13. Found: C, 46.29; H, 4.74; N, 23.97.

*Methyl 2-{1-[3-(trifluoromethyl)-1H-pyrazol-5-yl]ethylidene}hydrazine-1-carboxylate* (**17**). A mixture of acetylpyrazole **2** (5 mmol, 890 mg) and methyl carbazate (5 mmol, 450 mg) was refluxed in 15 mL of MeOH and 1 mL of HCl for 4 h. Then water was added, the precipitate formed was filtered off, washed by Et_2_O and dried. Yield 560 mg (45%); colorless powder; mp 198–199 °C; ^1^H NMR (400 MHz, DMSO-*d_6_*) δ 2.21 (s, 3H, Me), 3.72 (s, 3H, OMe), 7.01 (s, 1H, CH), 10.34 (s, 1H, NH), 13.90 (s, 1H, NH^pyr^); ^13^C NMR (126 MHz, DMSO-*d_6_*) δ 14.4, 52.0, 103.1, 121.6 (q, *J* = 268.1 Hz, CF_3_), 140.6, 141.2 (q, *J* = 37.0 Hz, CCF_3_), 143.0, 154.3; ^19^F NMR (376 MHz, DMSO-*d_6_*) δ 102.11 (s, 3F, CF_3_); Anal. calcd. for C_8_H_9_F_3_N_4_O_2_. C, 38.41; H, 3.63; N, 22.39. Found: C, 38.36; H, 3.43; N, 22.47. IR ν 3292–2958 (C–H, N–H), 1717 (C=O), 1620–1527 (C=C, C=N), 1241–1122 (C–O–C, C–F).

*N’-{1-[3-(Trifluoromethyl)-1H-pyrazol-5-yl]ethylidene}acetohydrazide* (**18**). A mixture of acetylpyrazole **2** (5 mmol, 890 mg) and acetohydrazide (5 mmol, 370 mg) was refluxed in 12 mL of MeCN and 3 mL of AcOH for 4 h. The precipitate formed was filtered off and recrystallized from MeCN–acetone/4:1. Yield 853 mg (73%); colorless powder; mp 228–229 °C; ^1^H NMR (400 MHz, DMSO-*d_6_*) δ (***E*** + ***Z***) 7.06 (s, 1H, CH), 13.87 (br.s, 1H, NH^pyr^); (***E***, 75%) 2.20 (s, 3H, Me), 2.26 (s, 3H, C(O)Me), 10.56 (s, 1H, NH); (***Z***, 25%) 2.05 (s, 3H, Me), 2.25 (s, 3H, C(O)Me), 10.47 (s, 1H, NH); ^13^C NMR (126 MHz, DMSO-*d_6_*) δ (***E*** + ***Z***) 121.5 (q, *J* = 268.1 Hz, CF_3_), 137.7, 141.3 (q, *J* = 36.7 Hz, CCF_3_); (***E***, 75%) 13.9, 20.7, 103.2, 143.1, 173.1; (***Z***, 25%) 14.5, 21.6, 103.6, 142.1, 166.1; ^19^F NMR (376 MHz, DMSO-*d_6_*) δ (***E***, 75%) 102.13 (s, 3F, CF_3_); (***Z***, 25%) 102.10 (s, 3F, CF_3_); Anal. calcd. for C_8_H_9_F_3_N_4_O. C, 41.03; H, 3.87; N, 23.92. Found C, 40.79; H, 3.76; N, 23.68. IR ν 3241–2861 (C–H, N–H), 1664 (C=O), 1611–1568 (C=C, C=N), 1259–1134 (C–F).

#### 4.3.2. Alkylation of NH-Ketazine **4**

*Method A*: A mixture of *NH*-ketazine **4** (1.5 mmol, 530 mg), potassium carbonate (4.5 mmol, 622 mg), and ethyl iodoacetate (3.3 mmol, 710 mg) was refluxed in 10 mL of dry MeCN for 4 h. The reaction mixture was analyzed by GC-MS: 3,3′-bis-(CF_3_)ketazine **14** (56%); 3,5′-bis-(CF_3_)ketazine **16** (35%); unidentified by-products (9%); 5,5′-bis-(CF_3_)ketazine **13** (0%).

*Method B*: Sodium hydride (3.3 mmol, 135 mg; 60% in mineral oil) was added to a solution of *NH*-ketazine **4** (1.5 mmol, 530 mg) in 7 mL of DME at 0–5 °C, then ethyl iodoacetate (3.3 mmol, 710 mg) dissolved in 3 mL of dry MeCN was added dropwise, and the suspension was stirred at room temperature for 3 h. The reaction mixture was analyzed by GC-MS: 3,3′-bis-(CF_3_)ketazine **14** (75%); 3,5′-bis-(CF_3_)ketazine **16** (21%); mono-3-(CF_3_)ketazine (4%); 5,5′-bis-(CF_3_)ketazine **13** (0%).

*Diethyl 2,2′-{[hydrazine-1,2-diylidenebis(ethan-1-yl-1-ylidene)]bis [3-(trifluoromethyl)-1H-pyrazole-5,1-diyl]}diacetate* (**14**) prepared by *Method B* was obtained after solvent evaporation as the residue, which was further washed by hot water and Et_2_O. Yield 392 mg (50%); yellow powder; mp 187–188 °C; ^1^H NMR (500 MHz, DMSO-*d_6_*) δ 1.15 (t, *J* = 7.1 Hz, 6H, 2OCH_2_CH_3_), 2.27 (s, 6H, 2Me), 4.11 (q, *J* = 7.1 Hz, 4H, 2OCH_2_CH_3_), 5.44 (s, 4H, 2CH_2_), 7.51 (s, 2H, 2CH); ^13^C NMR (126 MHz, DMSO-*d_6_*) δ 13.9, 16.2, 55.2, 61.1, 108.6, 121.1 (q, *J* = 268.2 Hz, CF_3_), 139.9 (q, *J* = 38.1 Hz, CCF_3_), 141.1, 152.7, 167.4; ^19^F NMR (470 MHz, DMSO-*d_6_*) δ 101.81 (s, 6F, 2CF_3_); ^1^H NMR (500 MHz, CDCl_3_) δ 1.24 (t, *J* = 7.2 Hz, 6H, 2OCH_2_CH_3_), 2.27 (s, 6H, 2Me), 4.19 (q, *J* = 7.1 Hz, 4H, 2OCH_2_CH_3_), 5.40 (s, 4H, 2CH_2_), 6.92 (s, 2H, 2CH); ^19^F NMR (470 MHz, CDCl_3_) δ 99.37 (s, 6F, 2CF_3_); Anal. calcd. for C_20_H_22_F_6_N_6_O_4_. C, 45.81; H, 4.23; N, 16.03. Found: C, 45.73; H, 4.00; N, 15.82. IR ν 3490, 3152–2908 (C–H), 1754 (C=O), 1620–1540 (C=C, C=N), 1272–1115 (C–O–C, C–F).

#### 4.3.3. General Procedure for the Alkylation of NH-Pyrazoles **2**, **3**, **15**, **17**, **18** Using K_2_CO_3_

A mixture of an appropriate *NH*-pyrazole (2 mmol), potassium carbonate (3 mmol, 415 mg), and ethyl iodoacetate (2.2 mmol, 470 mg) was refluxed in 10 mL of dry MeCN for 4 h.

*Diethyl 2,2′-{[hydrazine-1,2-diylidenebis(ethan-1-yl-1-ylidene)]bis [5-(trifluoromethyl)-1H-pyrazole-3,1-diyl]}diacetate* (**13**) prepared from compound **15** (465 mg) was obtained after solvent evaporation as the residue, which was further washed by hot water and recrystallized from hexane–Et_2_O/2:1. Yield 65 mg (25%). (See Section 4.3.5 for spectral and elemental analysis data).

*Ethyl 2-{3-[1-({1-[1-(2-ethoxy-2-oxoethyl)-3-(trifluoromethyl)-1H-pyrazol-5-yl]ethylidene}hydrazineylidene)ethyl]-5-(trifluoromethyl)-1H-pyrazol-1-yl}acetate* (**16**) prepared from compound **15** (465 mg) was obtained after product 13 isolation by crystallization (slow evaporation) from hexane–Et_2_O/2:1 filtrate. Yield < 10 mg (traces); yellow crystals; mp 117–118 °C; ^1^H NMR (400 MHz, CDCl_3_) δ 1.23 (t, *J* = 7.1 Hz, 3H, OCH_2_CH_3_), 1.29 (t, *J* = 7.1 Hz, 3H, OCH_2_CH_3_), 2.27 (s, 3H, Me), 2.33 (s, 3H, Me), 4.19 (q, *J* = 7.1 Hz, 2H, OCH_2_CH_3_), 4.27 (q, *J* = 7.1 Hz, 2H, OCH_2_CH_3_), 5.05 (s, 2H, CH_2_), 5.44 (s, 2H, CH_2_), 6.89 (s, 1H, CH), 7.20 (d, *J* = 0.9 Hz, 1H, CH); ^13^C NMR (126 MHz, CDCl_3_) δ 14.0, 14.1, 14.3, 16.2, 52.5, 55.3, 61.7, 62.3, 106.9 (q, *J* = 2.5 Hz, CH), 107.4 (unresolved q, CH), 119.6 (q, *J* = 269.3 Hz, CF_3_), 120.9 (q, *J* = 268.6 Hz, CF_3_), 133.9 (q, *J* = 39.8 Hz, CCF_3_), 141.3, 141.8 (q, *J* = 38.9 Hz, CCF_3_), 150.9, 151.8, 155.5, 166.6, 167.5; ^19^F NMR (376 MHz, CDCl_3_) δ 99.41 (s, 3F, CF_3_), 101.57 (s, 3F, CF_3_); Anal. calcd. for C_20_H_22_F_6_N_6_O_4_. C, 45.81; H, 4.23; N, 16.03. Found: C, 46.01; H, 4.12; N, 15.86. IR ν 3507, 3161–2853 (C–H), 1758 (C=O), 1729 (C=O), 1618–1546 (C=C, C=N), 1279–1121 (C–O–C, C–F).

*Methyl 2-{1-[1-(2-ethoxy-2-oxoethyl)-5-(trifluoromethyl)-1H-pyrazol-3-yl]ethylidene}hydrazine-1-carboxylate* (**19**) prepared from compound **17** (500 mg) was obtained after solvent evaporation as the residue, which was further washed by hot water and Et_2_O. Yield 418 mg (62%); colorless powder; mp 159–160 °C; ^1^H NMR (400 MHz, DMSO-*d_6_*) δ 1.20 (t, *J* = 7.1 Hz, 3H, OCH_2_CH_3_), 2.21 (s, 3H, Me), 3.71 (s, 3H, OMe), 4.17 (q, *J* = 7.1 Hz, 2H, OCH_2_CH_3_), 5.25 (s, 2H, CH_2_), 7.07 (s, 1H, CH), 10.30 (s, 1H, NH); ^13^C NMR (126 MHz, DMSO-*d_6_*) δ 13.0, 13.8, 52.0, 52.4, 61.6, 105.6 (d, *J* = 2.7 Hz, CH), 119.5 (q, *J* = 269.0 Hz, CF_3_), 132.6 (q, *J* = 39.0 Hz, CCF_3_), 143.3, 150.6, 154.4, 167.0; ^19^F NMR (376 MHz, DMSO-*d_6_*) δ 103.62 (s, 3F, CF_3_); Anal. calcd. for C_12_H_15_F_3_N_4_O_4_. C, 42.86; H, 4.50; N, 16.66. Found: C, 42.52; H, 4.67; N, 16.34. IR ν 3239–2985 (C–H, N–H), 1744 (C=O), 1697 (C=O), 1613–1510 (C=C, C=N), 1281–1124 (C–O–C, C–F).

*Ethyl 2-{3-[1-(2-acetylhydrazineylidene)ethyl]-5-(trifluoromethyl)-1H-pyrazol-1-yl}acetate* (**20**) prepared from compound **18** (470 mg) was obtained after cooling of the reaction mixture as the precipitate, which was further filtered off and recrystallized from MeCN. Yield 436 mg (68%); colorless powder; mp 170–171 °C; ^1^H NMR (400 MHz, DMSO-*d_6_*) δ (*E* + *Z*) 1.20 (t, *J* = 7.1 Hz, 3H, OCH_2_CH_3_), 4.18 (q, *J* = 7.1 Hz, 2H, OCH_2_CH_3_), 5.25 (s, 2H, CH_2_); (*E*, 70%) 2.21–2.22 (m, 6H, 2Me), 7.21 (s, 1H, CH), 10.55 (s, 1H, NH); (*Z*, 30%) 2.03 (s, 3H, Me), 2.25 (s, 3H, Me), 7.11 (s, 1H, CH), 10.43 (s, 1H, NH); ^13^C NMR (126 MHz, DMSO-*d_6_*) δ (*E* + ***Z***) 13.9, 52.5, 61.6, 119.5 (q, *J* = 269.3 Hz, CF_3_), 132.6 (q, *J* = 39.1 Hz, CCF_3_), 150.7; (*E*, 70%) 12.6, 20.7, 105.6 (d, *J* = 2.5 Hz, CH), 141.2, 167.1, 172.9; (*Z*, 30%) 13.2, 21.6, 105.8, 144.8, 166.1, 167.1; ^19^F NMR (376 MHz, DMSO-*d_6_*) δ (*E*, 70%) 103.68 (s, 3F, CF_3_); (*Z*, 30%) 103.60 (s, 3F, CF_3_); Anal. calcd. for C_12_H_15_F_3_N_4_O_3_. C, 45.00; H, 4.72; N, 17.49. Found: C 44.82, H 4.92, N 17.20. IR ν 3327, 3191–2920 (C–H, N–H), 1741 (C=O^ester^), 1685 (C=O), 1618–1561 (C=C, C=N), 1279–1121 (C–O–C, C–F).

#### 4.3.4. General Procedure for the Alkylation of NH-Pyrazole **21** Using NaH

Sodium hydride (2.2 or 4.4 mmol; 60% in mineral oil) was added to a solution of *NH*-pyrazole **21** (2 mmol, 540 mg) in 7 mL of DME at 0–5 °C, then ethyl iodoacetate (2.2 or 4.4 mmol) dissolved in 3 mL of dry MeCN was added dropwise, the resulting suspension was refluxed for 3 h.

*Ethyl 2-(3-{1-[2-(pyridin-2-yl)hydrazineylidene]ethyl}-5-(trifluoromethyl)-1H-pyrazol-1-yl)acetate* (**22**) was obtained after solvent evaporation as the residue, which was further washed by hot water and Et_2_O. Yield 351 mg (49%); beige powder; mp 223–225 °C; ^1^H NMR (500 MHz, DMSO-*d_6_*) δ 1.21 (t, *J* = 7.1 Hz, 3H, OCH_2_CH_3_), 2.43 (s, 3H, Me), 4.19 (q, *J* = 7.1 Hz, 2H, OCH_2_CH_3_), 5.32 (s, 2H, CH_2_), 7.18 (t, *J* = 6.7 Hz, 1H, CH^Ar^), 7.50 (d, *J* = 8.8 Hz, 1H, CH^Ar^), 7.82 (s, 1H, CH), 8.15 (t, *J* = 8.0 Hz, 1H, CH^Ar^), 8.18–8.20 (m, 1H, CH^Ar^), 11.70 (br.s, 1H, NH); ^13^C NMR (126 MHz, DMSO-*d_6_*) δ 13.5, 13.8, 52.6, 61.6, 107.1, 112.5, 115.4, 119.5 (q, *J* = 269.1 Hz, CF_3_), 132.7 (q, *J* = 39.0 Hz, CCF_3_), 137.3, 144.6, 147.0, 149.8, 150.1, 166.9; ^19^F NMR (470 MHz, DMSO-*d_6_*) δ 103.74 (s, 3F, CF_3_); Anal. calcd. for C_15_H_16_F_3_N_5_O_2_. C, 50.70; H, 4.54; N, 19.71. Found: C, 50.51; H, 4.27; N, 19.83. IR ν 3364, 3223–2814 (C–H, N–H), 1751 (C=O), 1642–1562 (C=C, C=N), 1279–1139 (C–O–C, C–F).

Ethyl 2-[2-({1-[1-(2-ethoxy-2-oxoethyl)-5-(trifluoromethyl)-1*H*-pyrazol-3-yl]ethylidene}hydrazineylidene)pyridin-1(2*H*)-yl]acetate (**23**) was obtained after cooling of the reaction mixture as the precipitate, which was further filtered off and washed by hot water and Et_2_O. Yield 497 mg (56%); yellow powder; ^1^H NMR (400 MHz, DMSO-*d_6_*) δ 1.19 (t, *J* = 7.1 Hz, 3H, OCH_2_CH_3_), 1.20 (t, *J* = 7.1 Hz, 3H, OCH_2_CH_3_), 2.20 (s, 3H, Me), 4.11–4.20 (m, 4H, 2OCH_2_CH_3_), 4.68 (s, 2H, CH_2_), 5.21 (s, 2H, CH_2_), 6.06 (td, *J* = 6.6, 1.5 Hz, 1H, CH^Ar^), 7.18 (ddd, *J* = 9.5, 6.4, 1.7 Hz, 1H, CH^Ar^), 7.27 (s, 1H, CH), 7.46 (d, *J* = 9.3 Hz, 1H, CH^Ar^), 7.52 (d, *J* = 6.2 Hz, 1H, CH^Ar^); ^13^C NMR (126 MHz, DMSO-*d_6_*) δ 12.8, 13.9, 14.1, 52.3, 52.9, 60.6, 61.5, 104.6, 105.5 (d, *J* = 2.7 Hz, CH), 113.4, 119.7 (q, *J* = 269.0 Hz, CF_3_), 132.1 (q, *J* = 38.9 Hz, CCF_3_), 136.0, 139.1, 147.7, 152.4, 156.6, 167.2, 168.1; ^19^F NMR (376 MHz, DMSO-*d_6_*) δ 103.78 (s, 3F, CF_3_); Anal. calcd. for C_19_H_22_F_3_N_5_O_4_. C, 51.70; H, 5.02; N, 15.87. Found: C, 51.46; H, 4.81; N, 15.98.

#### 4.3.5. Transamination Reaction

Diethyl 2,2′-{[hydrazine-1,2-diylidenebis(ethan-1-yl-1-ylidene)]bis [5-(trifluoromethyl)-1*H*-pyrazole-3,1-diyl]}diacetate (**13**). A mixture of pyrazole **20** (1 mmol, 320 mg) and hydrazine dihydrochloride (1 mmol, 105 mg) was refluxed in 5 mL of EtOH and 0.5 mL of HCl for 5 h. Then water was added, the precipitate formed was filtered off and dried without further purification. Yield 191 mg (73%); yellow powder; mp 182–183 °C; ^1^H NMR (500 MHz, CDCl_3_) δ 1.29 (t, *J* = 7.1 Hz, 6H, 2OCH_2_CH_3_), 2.31 (s, 6H, 2Me), 4.26 (q, *J* = 7.1 Hz, 4H, 2OCH_2_CH_3_), 5.04 (s, 4H, 2CH_2_), 7.23 (s, 2H, 2CH); ^13^C NMR (126 MHz, CDCl_3_) δ 14.0, 14.2, 52.5 (d, *J* = 1.8 Hz, CH_2_), 62.2, 106.9 (q, *J* = 2.5 Hz, CH), 119.7 (q, *J* = 269.2 Hz, CF_3_), 133.7 (q, *J* = 39.5 Hz, CCF_3_), 151.3, 153.7, 166.6; ^19^F NMR (470 MHz, CDCl_3_) δ 101.62 (s, 6F, 2CF_3_); ^1^H NMR (500 MHz, DMSO-*d_6_*) δ 1.21 (t, *J* = 7.1 Hz, 6H, 2OCH_2_CH_3_), 2.27 (s, 6H, 2Me), 4.20 (q, *J* = 7.1 Hz, 4H, 2OCH_2_CH_3_), 5.31 (s, 4H, 2CH_2_), 7.37 (s, 2H, 2CH); ^19^F NMR (470 MHz, DMSO-*d_6_*) δ 103.71 (s, 6F, 2CF_3_); Anal. calcd. for C_20_H_22_F_6_N_6_O_4_. C, 45.81; H, 4.23; N, 16.03. Found: C, 46.10; H, 4.41; N, 15.76. IR ν 3483, 3160–2925 (C–H), 1744 (C=O), 1620–1557 (C=C, C=N), 1278–1125 (C–O–C, C–F).

#### 4.3.6. General Procedure for the Hydrolysis of Ester Derivatives **13**, **14**

Compound **13** or **14** (0.3 mmol, 160 mg) was dissolved in 3 mL of EtOH, then 1 mL of 10% NaOH aqueous solution was added, and the mixture was refluxed for 8 h. The solution was neutralized with 0.1 M HCl, then water was added, the precipitate formed was filtered off and dried without further purification.

2,2′-{[Hydrazine-1,2-diylidenebis(ethan-1-yl-1-ylidene)]bis [5-(trifluoromethyl)-1*H*-pyrazole-3,1-diyl]}diacetic acid (**24**). Yield 125 mg (90%); yellow powder; mp 258–259 °C; ^1^H NMR (400 MHz, DMSO-*d_6_*) δ 2.27 (s, 6H, 2Me), 5.18 (s, 4H, 2CH_2_), 7.35 (s, 2H, 2CH), 13.54 (br.s, 2H, 2OH); ^13^C NMR (126 MHz, DMSO-*d_6_*) δ 14.1, 52.8, 106.4 (d, *J* = 2.6 Hz, CH), 119.6 (q, *J* = 269.2 Hz, CF_3_), 132.7 (q, *J* = 39.0 Hz, CCF_3_), 150.1, 153.3, 168.4; ^19^F NMR (376 MHz, DMSO-*d_6_*) δ 103.69 (s, 6F, 2CF_3_); Anal. calcd. for C_16_H_14_F_6_N_6_O_4_. C, 41.04; H, 3.01; N, 17.95. Found: C, 41.32; H, 3.21; N, 17.68. IR ν 3163–2526 (C–H, O–H), 1739 (C=O), 1616–1564 (C=C, C=N), 1277–1136 (C–F).

2,2′-{[Hydrazine-1,2-diylidenebis(ethan-1-yl-1-ylidene)]bis [3-(trifluoromethyl)-1*H*-pyrazole-5,1-diyl]}diacetic acid (**25**). Yield 129 mg (92%); yellow powder; mp 290–291 °C; ^1^H NMR (500 MHz, DMSO-*d_6_*) δ 2.29 (s, 6H, 2Me), 5.36 (s, 4H, 2CH_2_), 7.49 (s, 2H, 2CH), 13.24 (br.s, 2H, 2OH); ^13^C NMR (126 MHz, DMSO-*d_6_*) δ 16.4, 55.2, 108.6, 121.2 (q, *J* = 268.4 Hz, CF_3_), 139.7 (q, *J* = 37.9 Hz, CCF_3_), 141.1, 152.9, 168.9; ^19^F NMR (470 MHz, DMSO-*d_6_*) δ 101.91 (s, 6F, 2CF_3_); Anal. calcd. for C_16_H_14_F_6_N_6_O_4_. C, 41.04; H, 3.01; N, 17.95. Found: C, 40.83; H, 2.94; N, 17.68. IR ν 3168–2558 (C–H, O–H), 1716 (C=O), 1612–1542 (C=C, C=N), 1271–1111 (C–F).

## 5. Conclusions

Thus, we have demonstrated that the regioselectivity of pyrazole alkylation can be controlled by tuning the substituents in the azole ring. The substrates were non-symmetric *NH*-pyrazoles having both trifluoromethyl and functional groups. The alkylation of acetylpyrazoles was low selective, while modification of the carbonyl group to hydrazone one guided the reaction toward forming 3- or 5-CF_3_-pyrazoles containing a carboxylate substituent at the azole *N*-atom. This opens the possibility to synthesize the polyfunctional pyrazoles and *bis*-pyrazoles with two carboxylate groups, which are of interest for further modifications and applications.

## Data Availability

The original contributions presented in this study are included in the Supplementary Materials. Further inquiries can be directed to the corresponding author.

## Supplementary Materials

The following supporting information can be downloaded at: https://www.mdpi.com/article/10.3390/ijms262110335/s1.
