# Supplementary material for: Switching N-Alkylation Regioselectivity of Trifluoromethylated Pyrazoles Guided by Functional Group Tuning
Source: Int J Mol Sci. 2025 Oct 23;26(21):10335. doi: 10.3390/ijms262110335 (PMC12607764; doi:10.3390/ijms262110335)
Supplement: Supplementary file 1 [file ijms-26-10335-s001.zip › ijms-3911049-supplementary.pdf]

International Journal of Molecular Sciences

Supporting information

Switching *N*-alkylation Regioselectivity of Trifluoromethylated Pyrazoles Guided by Functional Group Tuning

Yulia O. Edilova [1], Yulia S. Kudyakova [1,*], Ekaterina A. Osipova [1,2], Pavel A. Slepukhin [1,2], Yanina V. Burgart [1], Victor I. Saloutin [1] and Denis N. Bazhin [1,2,*]

[1] Postovsky Institute of Organic Synthesis, Ural Branch of the Russian Academy of Sciences, Yekaterinburg, 620137, Russian Federation
[2] Ural Federal University named after the First President of Russia B.N. Eltsin, Mira Str. 19, Ekaterinburg, 620002, Russian Federation
* Correspondence: yu.kudyakova@gmail.com (Y.S.K.); dnbazhin@gmail.com (D.N.B.)

Table of contents

Figure S1. ^{19}F NMR (376 MHz, [D6]DMSO) reaction mixture spectrum for **2** 3
Figure S2. ^{19}F NMR (376 MHz, $CDCl_3$) reaction mixture spectrum for **3** 4
Figure S3. ^{1}H NMR (500 MHz, $CDCl_3$) spectrum of compound **13** 5
Figure S4. ^{13}C NMR (126 MHz, $CDCl_3$) spectrum of compound **13** 6
Figure S5. ^{19}F NMR (470 MHz, $CDCl_3$) spectrum of compound **13** 7
Figure S6. ^{1}H NMR (500 MHz, [D6]DMSO) spectrum of compound **13** 8
Figure S7. ^{19}F NMR (470 MHz, [D6]DMSO) spectrum of compound **13** 9
Figure S8. ^{1}H NMR (500 MHz, [D6]DMSO) spectrum of compound **14** 10
Figure S9. ^{13}C NMR (126 MHz, [D6]DMSO) spectrum of compound **14** 11
Figure S10. ^{19}F NMR (470 MHz, [D6]DMSO) spectrum of compound **14** 12
Figure S11. ^{1}H NMR (500 MHz, $CDCl_3$) spectrum of compound **14** 13
Figure S12. ^{19}F NMR (470 MHz, $CDCl_3$) spectrum of compound **14** 14
Figure S13. ^{1}H NMR (400 MHz, [D6]DMSO) spectrum of compound **15** 15
Figure S14. ^{13}C NMR (126 MHz, [D6]DMSO) spectrum of compound **15** 16
Figure S15. ^{19}F NMR (376 MHz, [D6]DMSO) spectrum of compound **15** 17
Figure S16. ^{1}H NMR (400 MHz, $CDCl_3$) spectrum of compound **16** 18
Figure S17. ^{13}C NMR (126 MHz, $CDCl_3$) spectrum of compound **16** 19
Figure S18. ^{19}F NMR (376 MHz, $CDCl_3$) spectrum of compound **16** 20
Figure S19. ^{1}H NMR (400 MHz, [D6]DMSO) spectrum of compound **17** 21
Figure S20. ^{13}C NMR (126 MHz, [D6]DMSO) spectrum of compound **17** 22

Figure S21. ^{19}F NMR (376 MHz, [D6]DMSO) spectrum of compound **17** 23
Figure S22. ^{1}H NMR (400 MHz, [D6]DMSO) spectrum of compound **18** 24
Figure S23. ^{13}C NMR (126 MHz, [D6]DMSO) spectrum of compound **18** 25
Figure S24. ^{19}F NMR (376 MHz, [D6]DMSO) spectrum of compound **18** 26
Figure S25. ^{1}H NMR (400 MHz, [D6]DMSO) spectrum of compound **19** 27
Figure S26. ^{13}C NMR (126 MHz, [D6]DMSO) spectrum of compound **19** 28
Figure S27. ^{19}F NMR (376 MHz, [D6]DMSO) spectrum of compound **19** 29
Figure S28. ^{1}H NMR (400 MHz, [D6]DMSO) spectrum of compound **20** 30
Figure S29. ^{13}C NMR (126 MHz, [D6]DMSO) spectrum of compound **20** 31
Figure S30. ^{19}F NMR (376 MHz, [D6]DMSO) spectrum of compound **20** 32
Figure S31. ^{1}H NMR (500 MHz, [D6]DMSO) spectrum of compound **22** 33
Figure S32. ^{13}C NMR (126 MHz, [D6]DMSO) spectrum of compound **22** 34
Figure S33. ^{19}F NMR (470 MHz, [D6]DMSO) spectrum of compound **22** 35
Figure S34. ^{1}H NMR (400 MHz, [D6]DMSO) spectrum of compound **23** 36
Figure S35. ^{13}C NMR (126 MHz, [D6]DMSO) spectrum of compound **23** 37
Figure S36. ^{19}F NMR (376 MHz, [D6]DMSO) spectrum of compound **23** 38
Figure S37. ^{1}H NMR (400 MHz, [D6]DMSO) spectrum of compound **24** 39
Figure S38. ^{13}C NMR (126 MHz, [D6]DMSO) spectrum of compound **24** 40
Figure S39. ^{19}F NMR (376 MHz, [D6]DMSO) spectrum of compound **24** 41
Figure S40. ^{1}H NMR (500 MHz, [D6]DMSO) spectrum of compound **25** 42
Figure S41. ^{13}C NMR (126 MHz, [D6]DMSO) spectrum of compound **25** 43
Figure S42. ^{19}F NMR (470 MHz, [D6]DMSO) spectrum of compound **25** 44
Figure S43. The structure of compound **15** showing the arrangement of substituents at the hydrazone fragment in two planes 45
Figure S44. Fragment of the crystal packing of compound **4** 45
Figure S45. Fragment of the crystal packing of compound **13** 46
Figure S46. Fragment of the crystal packing of compound **14** 46
Figure S47. Fragment of the crystal packing of compound **15** showing the H(1)…N(3) intermolecular hydrogen bond equal to 2.202(27) Å 47
Figure S48. Fragment of the crystal packing of compound **16** 47
Figure S49. Fragment of the crystal packing of compound **25** 48
Table S1. Crystallographic parameters and structure refinement statistics for pyrazoles **4, 13** and **14** 49
Table S1 (continued). Crystallographic parameters and structure refinement statistics for pyrazoles **15, 16** and **25** 50

Figure S1. ^{19}F NMR (376 MHz, [D6]DMSO) reaction mixture spectrum for **2**

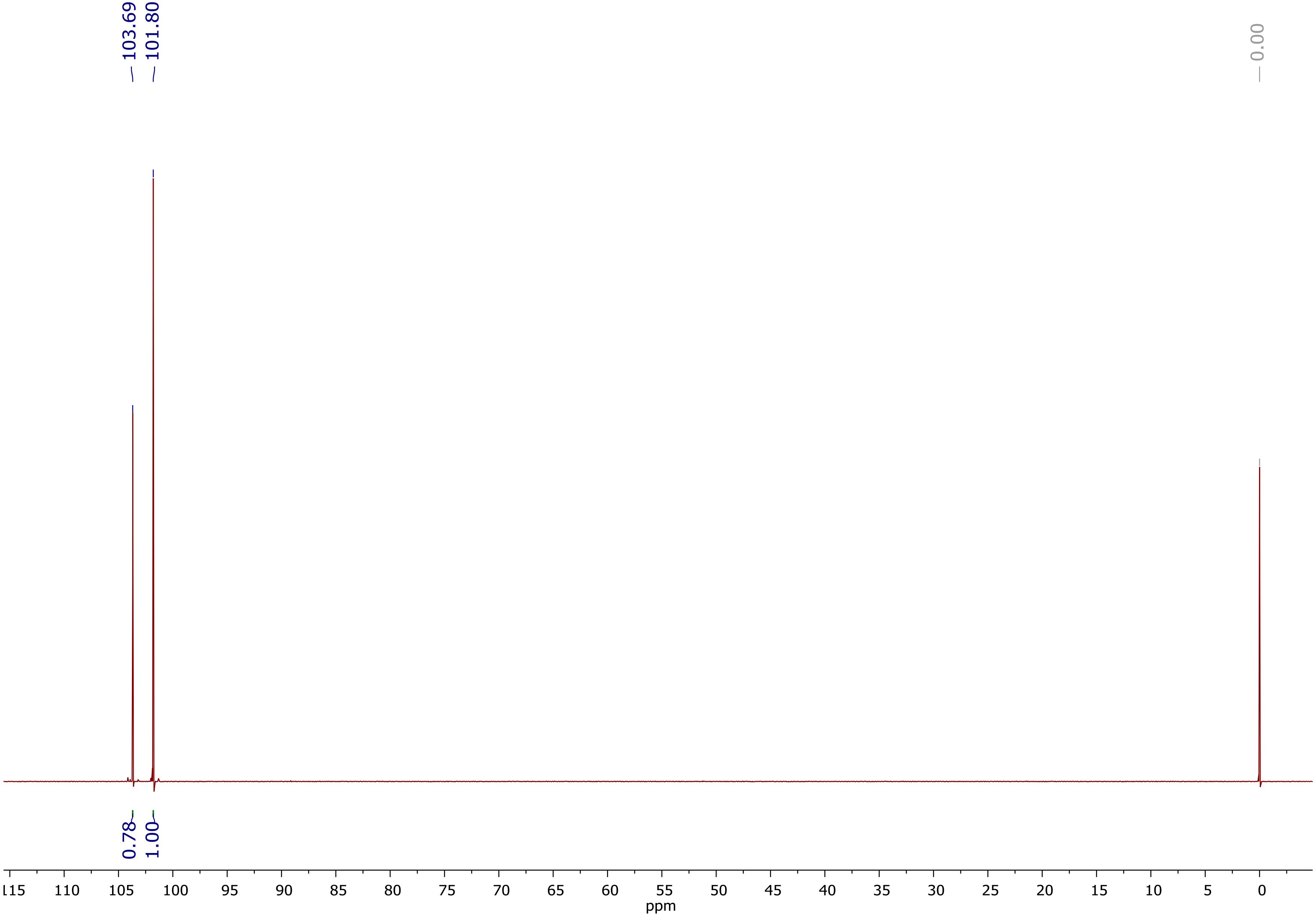

Figure S2. ^{19}F NMR (376 MHz, $CDCl_3$) reaction mixture spectrum for **3**

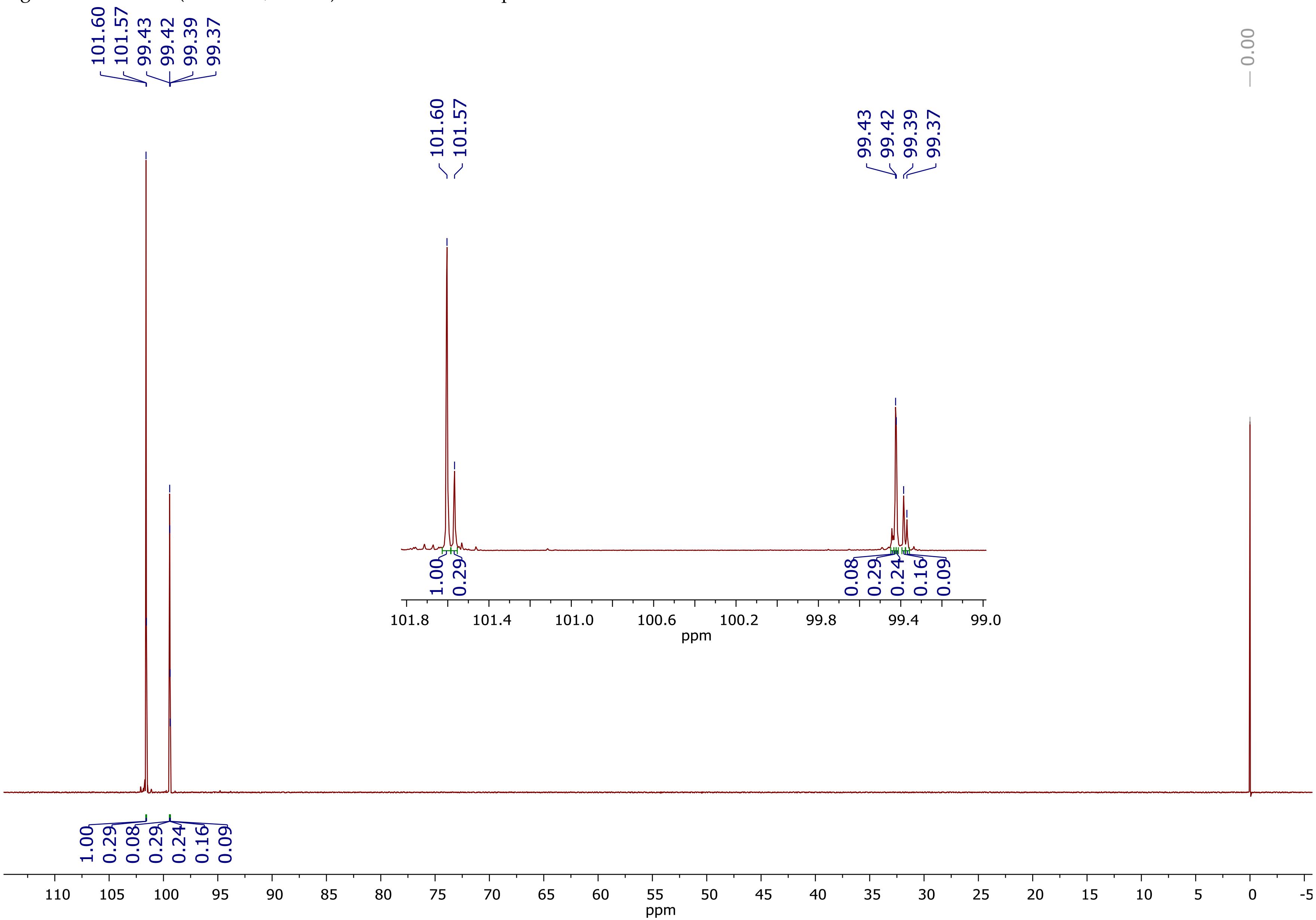

Figure S3. ^{1}H NMR (500 MHz, $CDCl_3$) spectrum of compound **13**

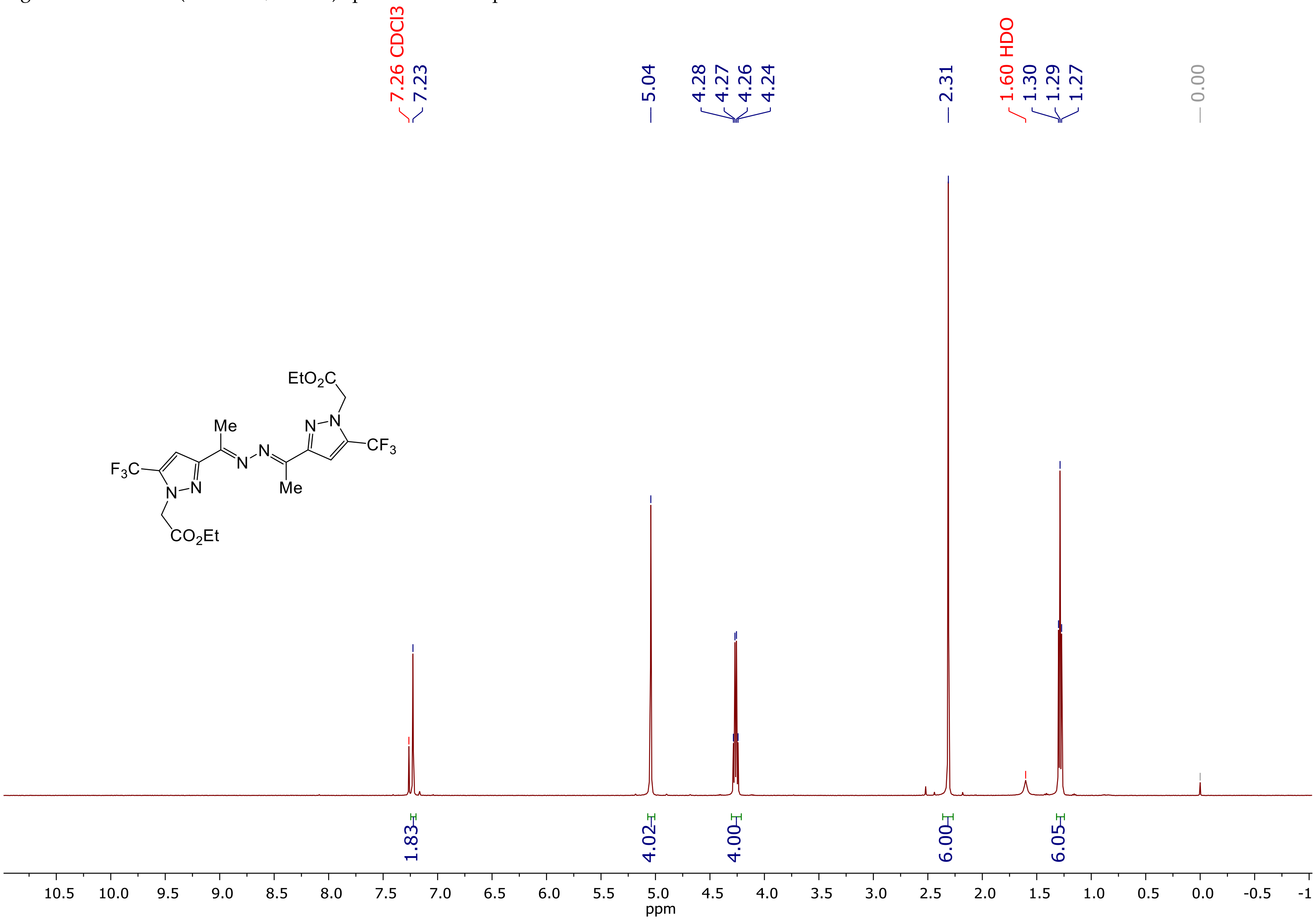

Figure S4. ^{13}C NMR (126 MHz, $CDCl_3$) spectrum of compound **13**

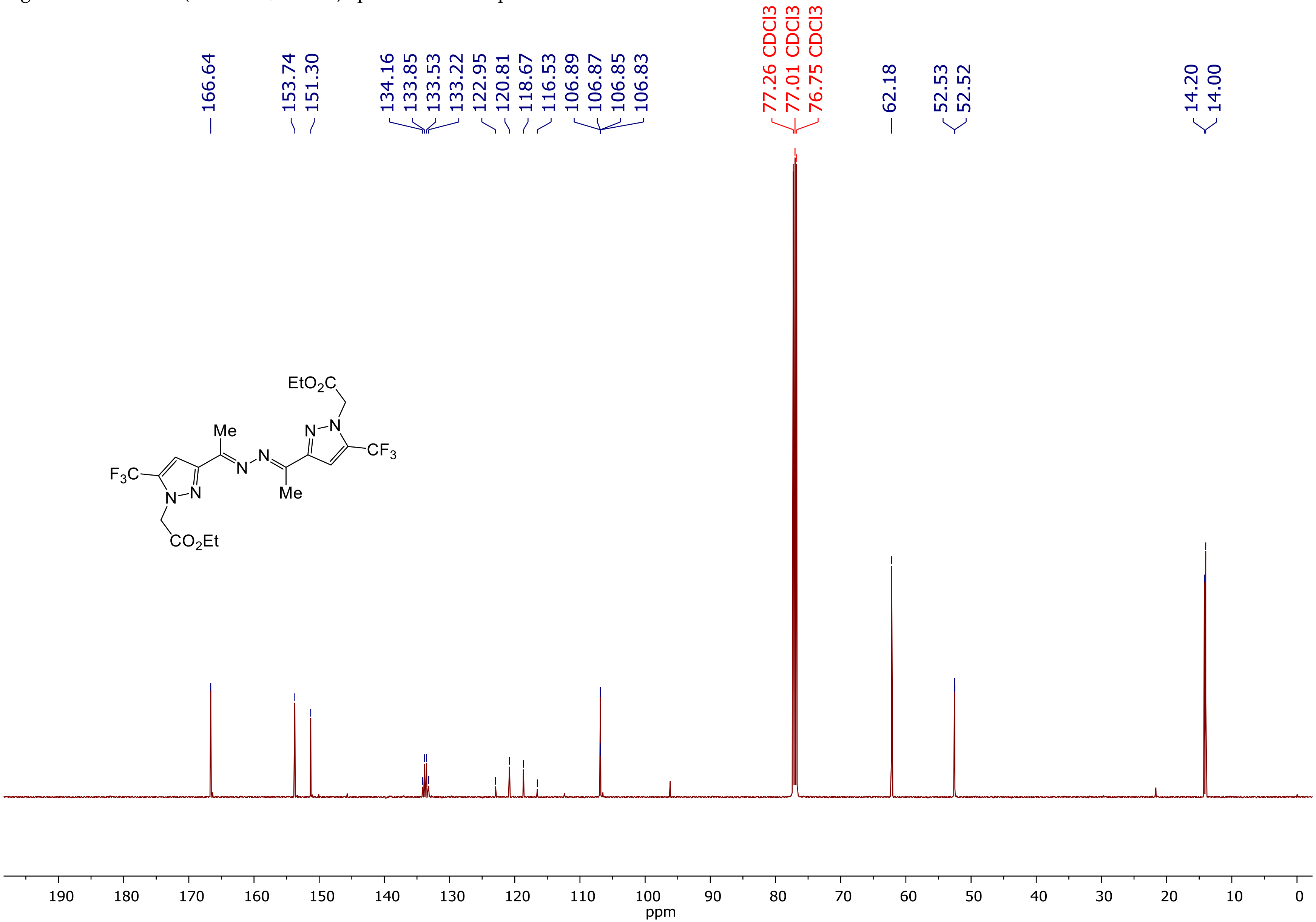

Figure S5. ^{19}F NMR (470 MHz, $CDCl_3$) spectrum of compound **13**

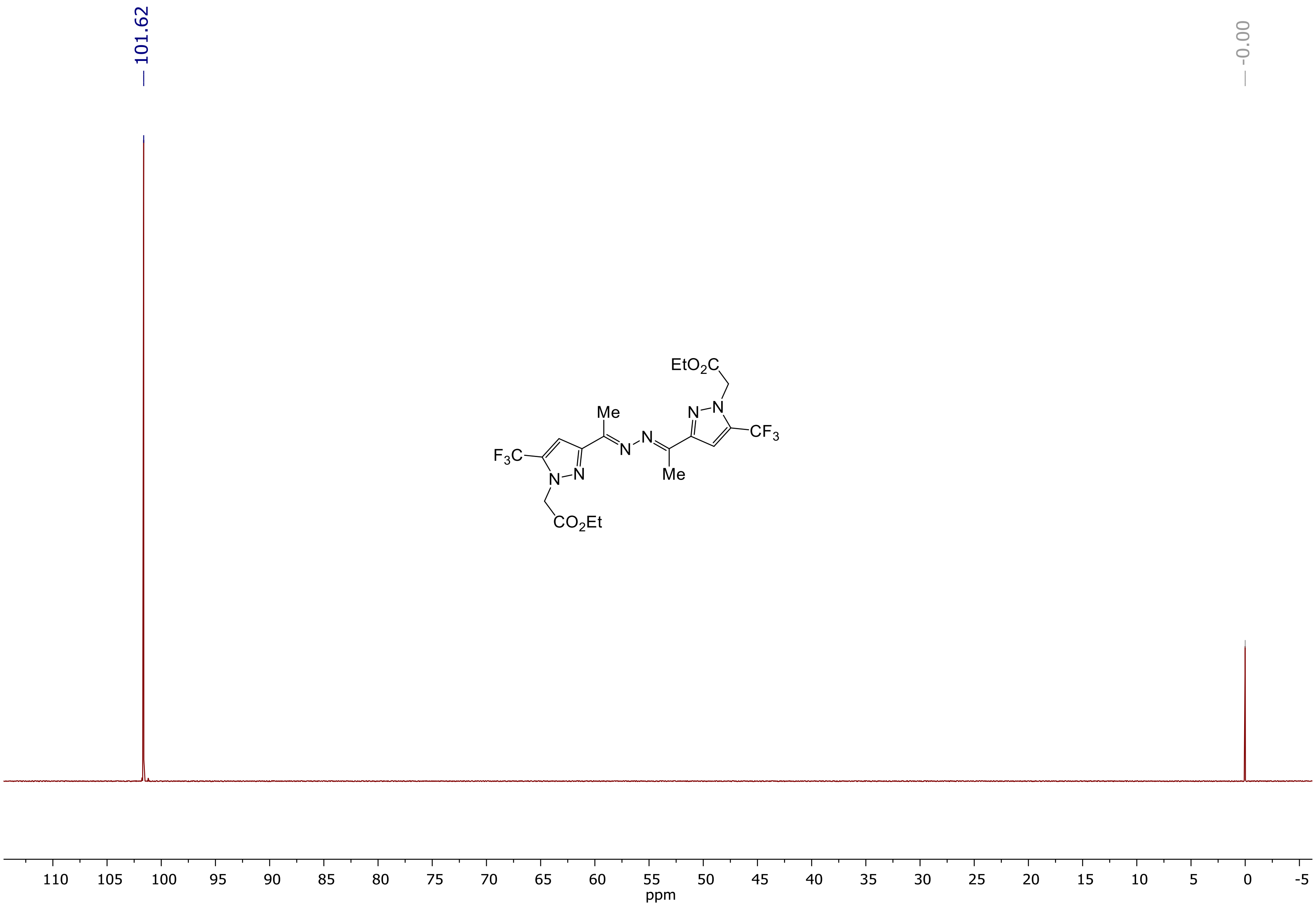

Figure S6. ^{1}H NMR (500 MHz, [D6]DMSO) spectrum of compound **13**

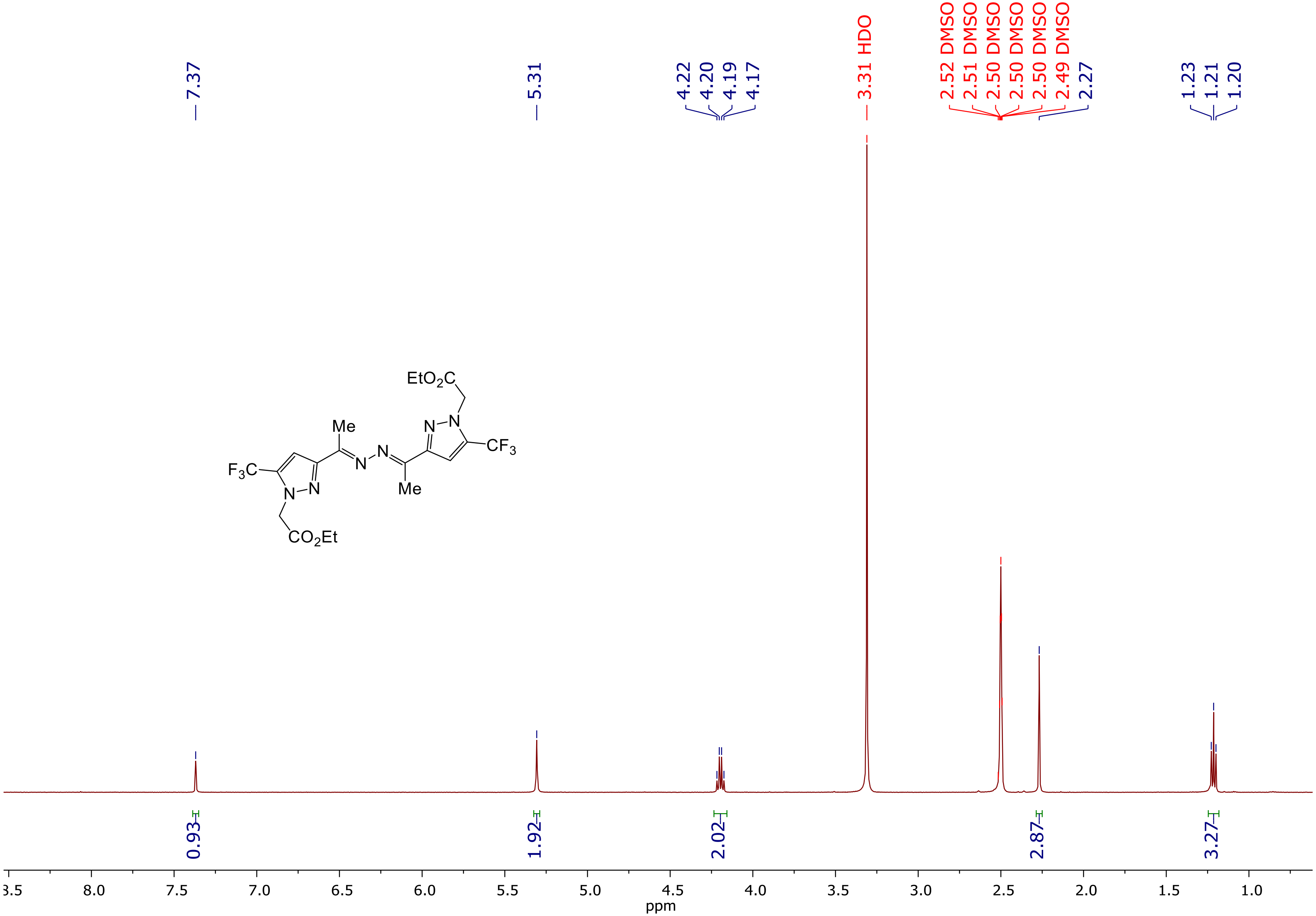

Figure S7. ^{19}F NMR (470 MHz, [D6]DMSO) spectrum of compound **13**

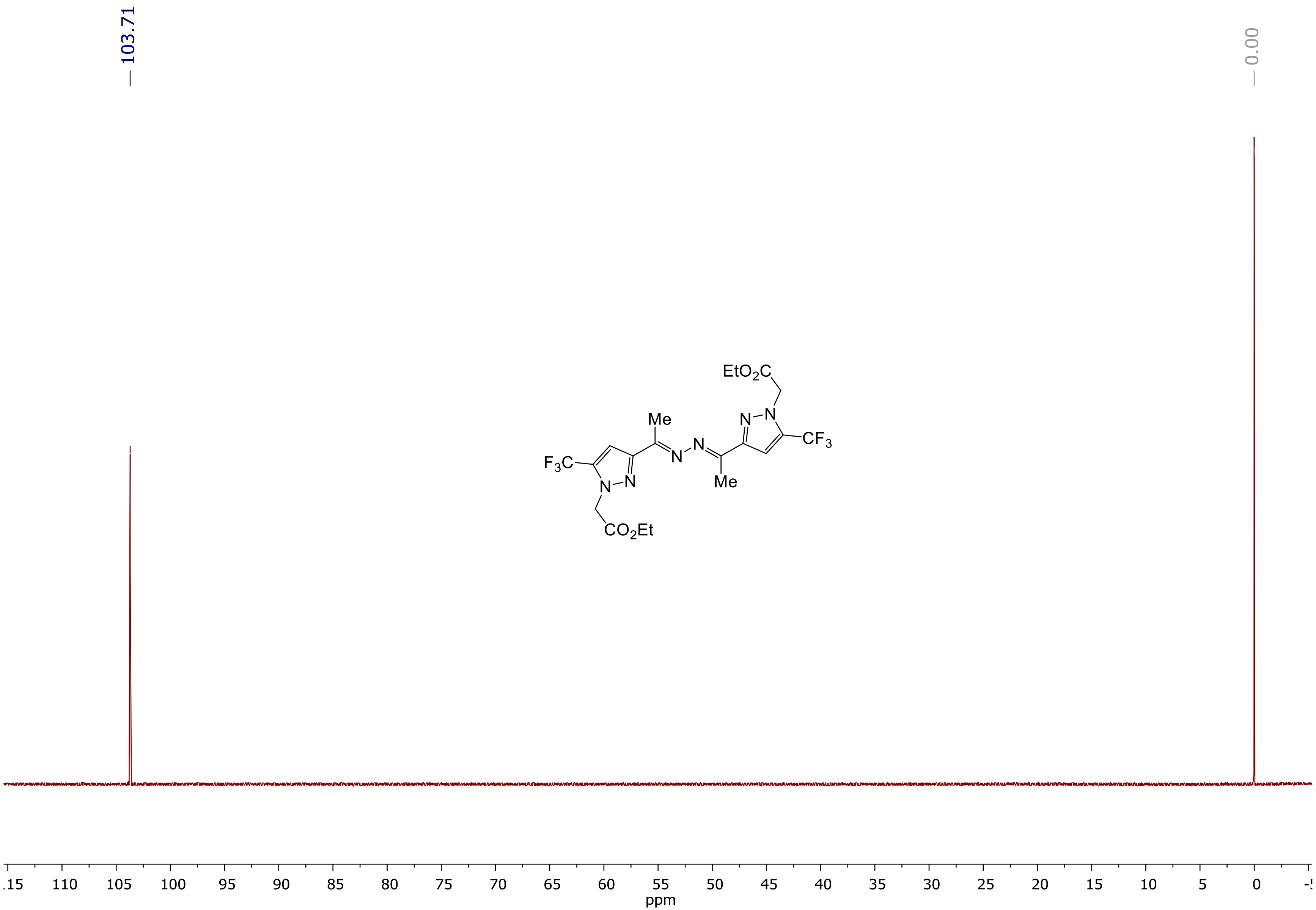

Figure S8. ^{1}H NMR (500 MHz, [D6]DMSO) spectrum of compound **14**

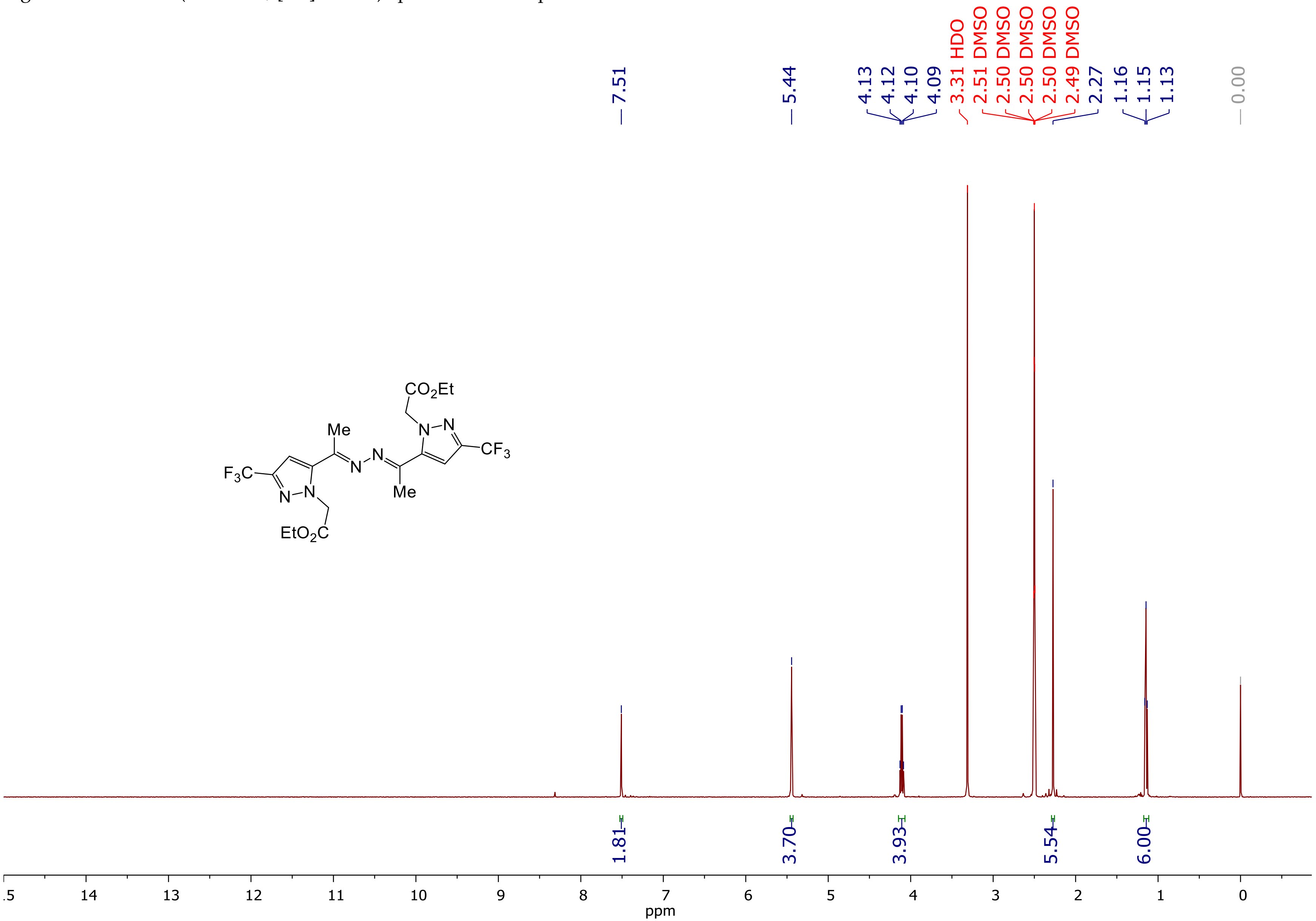

Figure S9. ^{13}C NMR (126 MHz, [D6]DMSO) spectrum of compound **14**

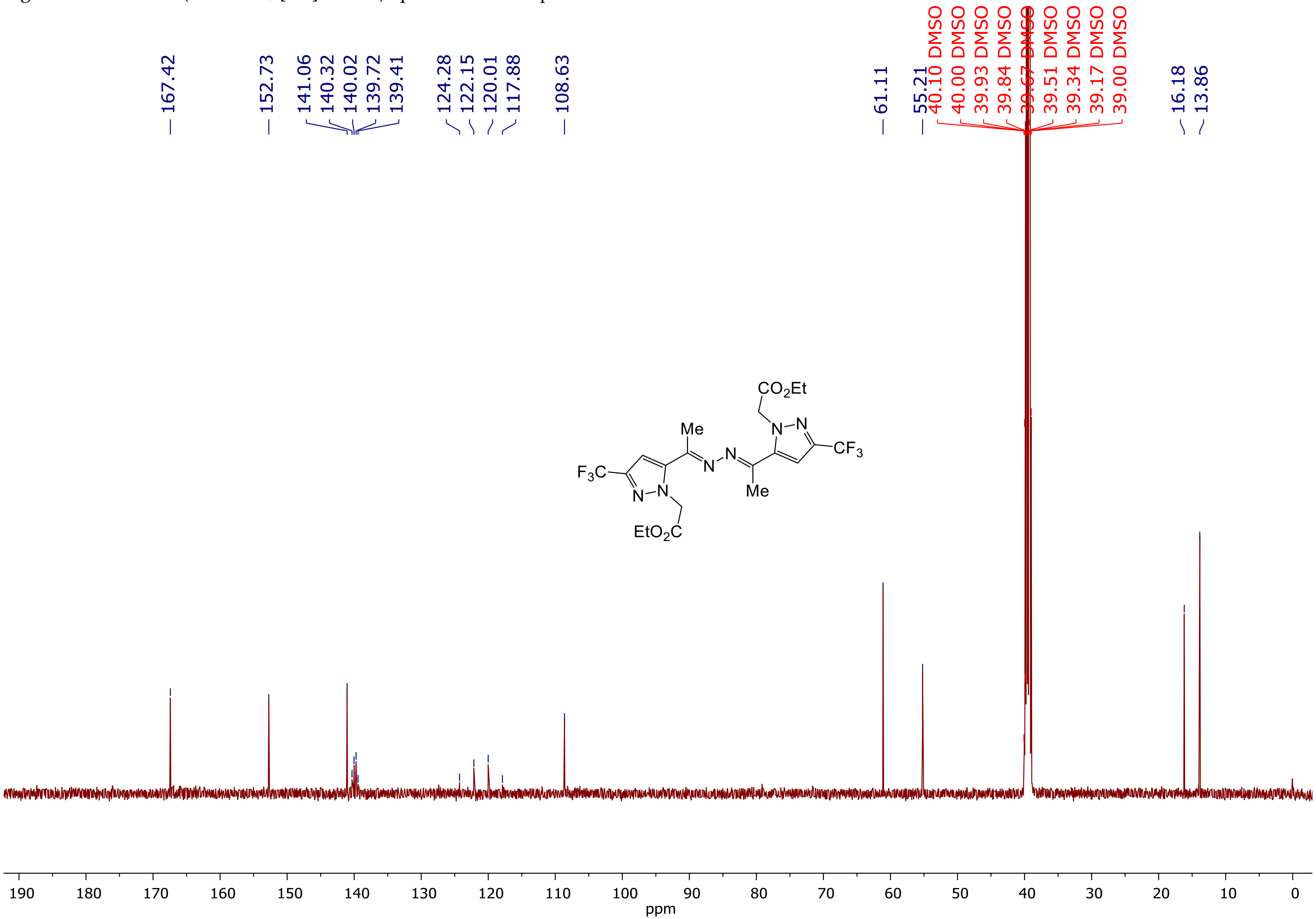

Figure S10. ^{19}F NMR (470 MHz, [D6]DMSO) spectrum of compound **14**

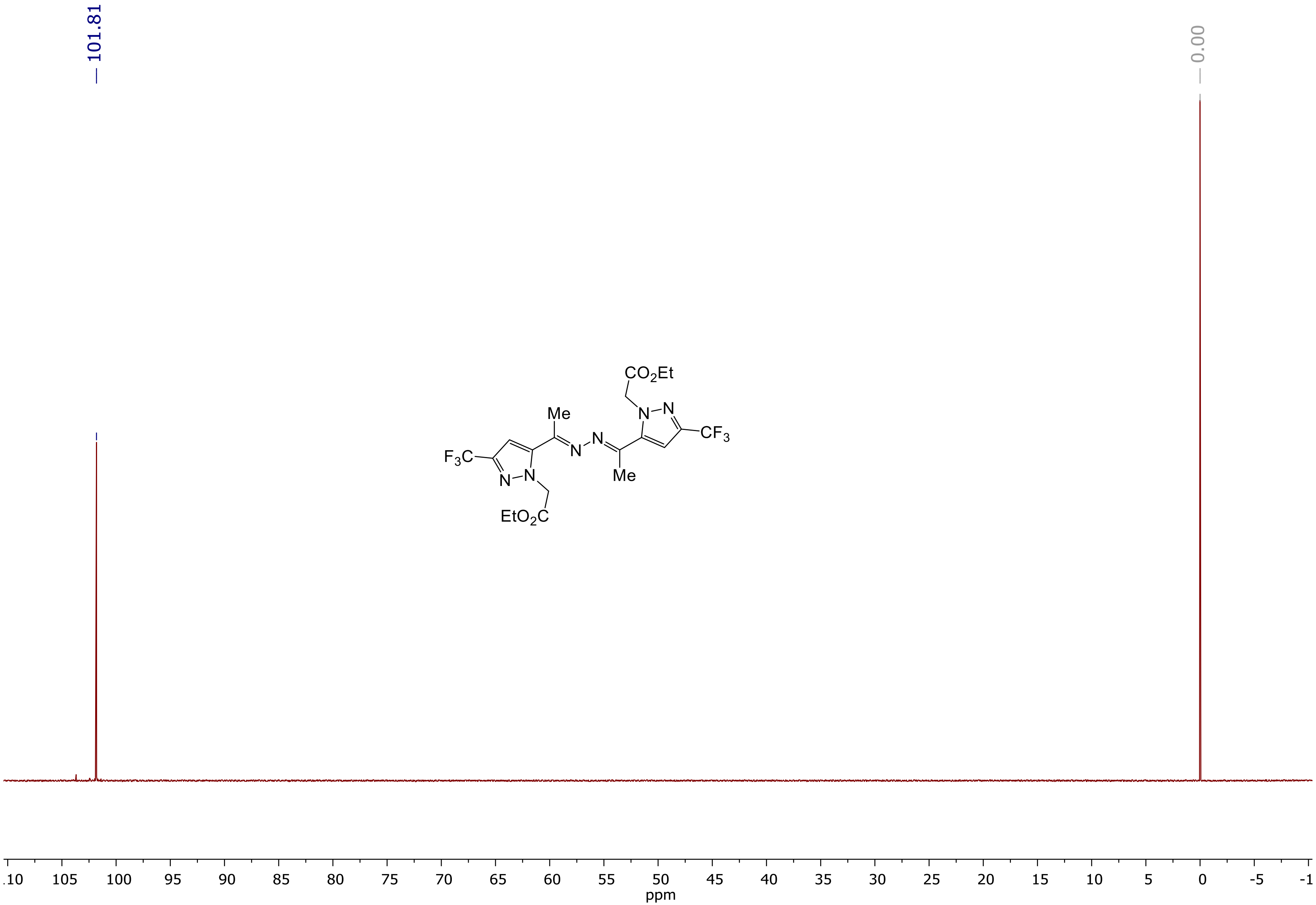

Figure S11. ^{1}H NMR (500 MHz, $CDCl_3$) spectrum of compound **14**

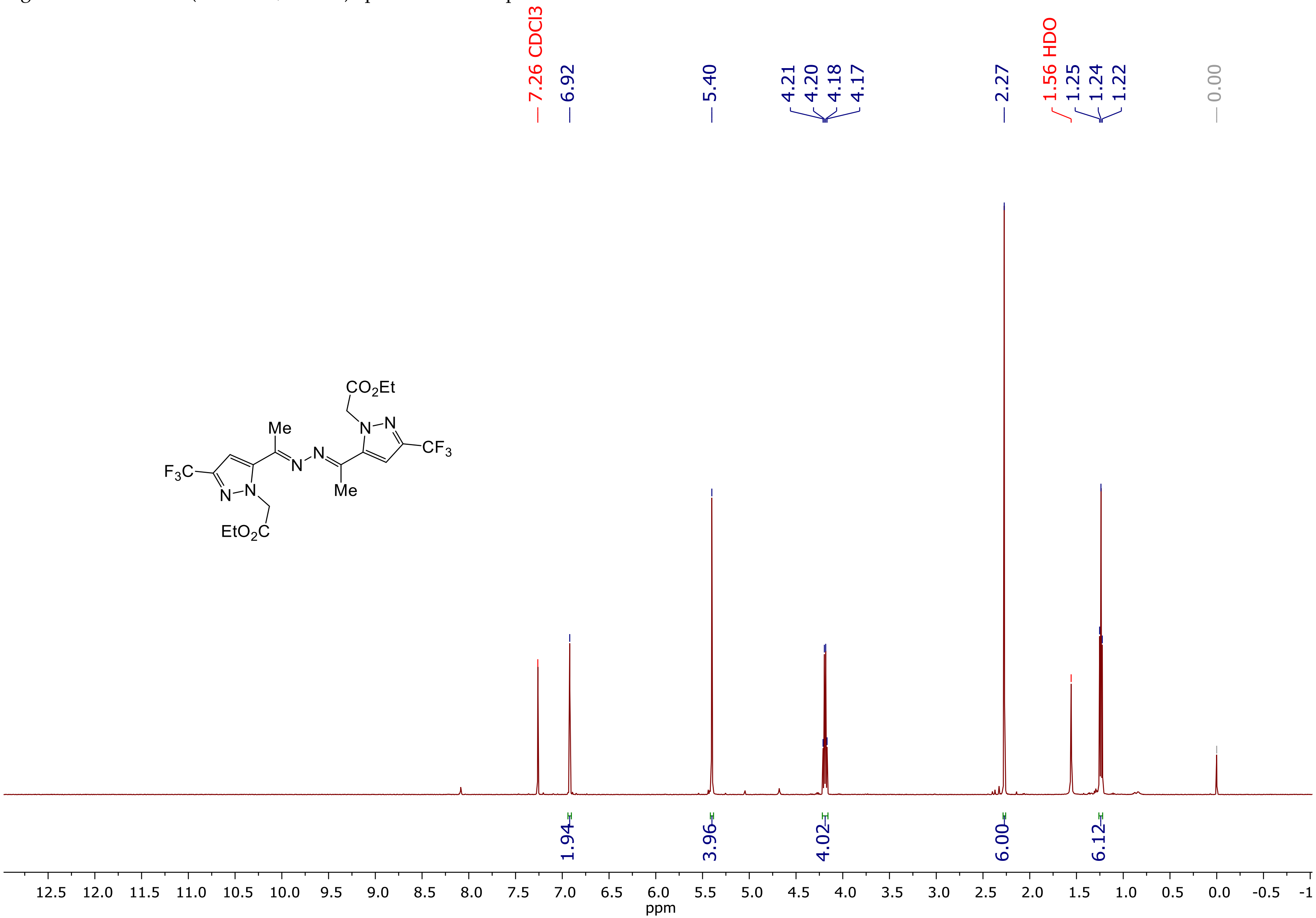

Figure S12. ^{19}F NMR (470 MHz, $CDCl_3$) spectrum of compound **14**

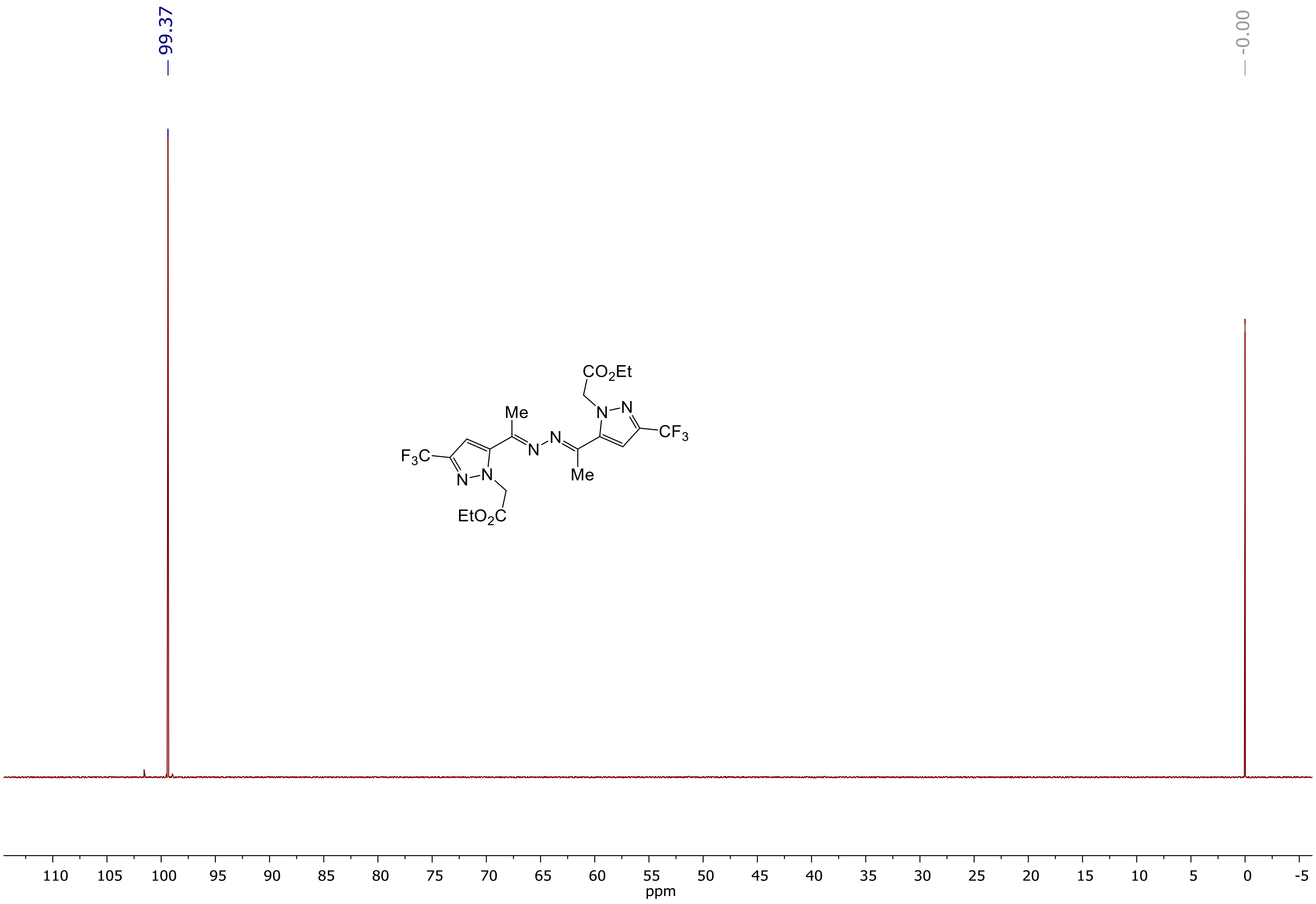

Figure S13. ^{1}H NMR (400 MHz, [D6]DMSO) spectrum of compound **15**

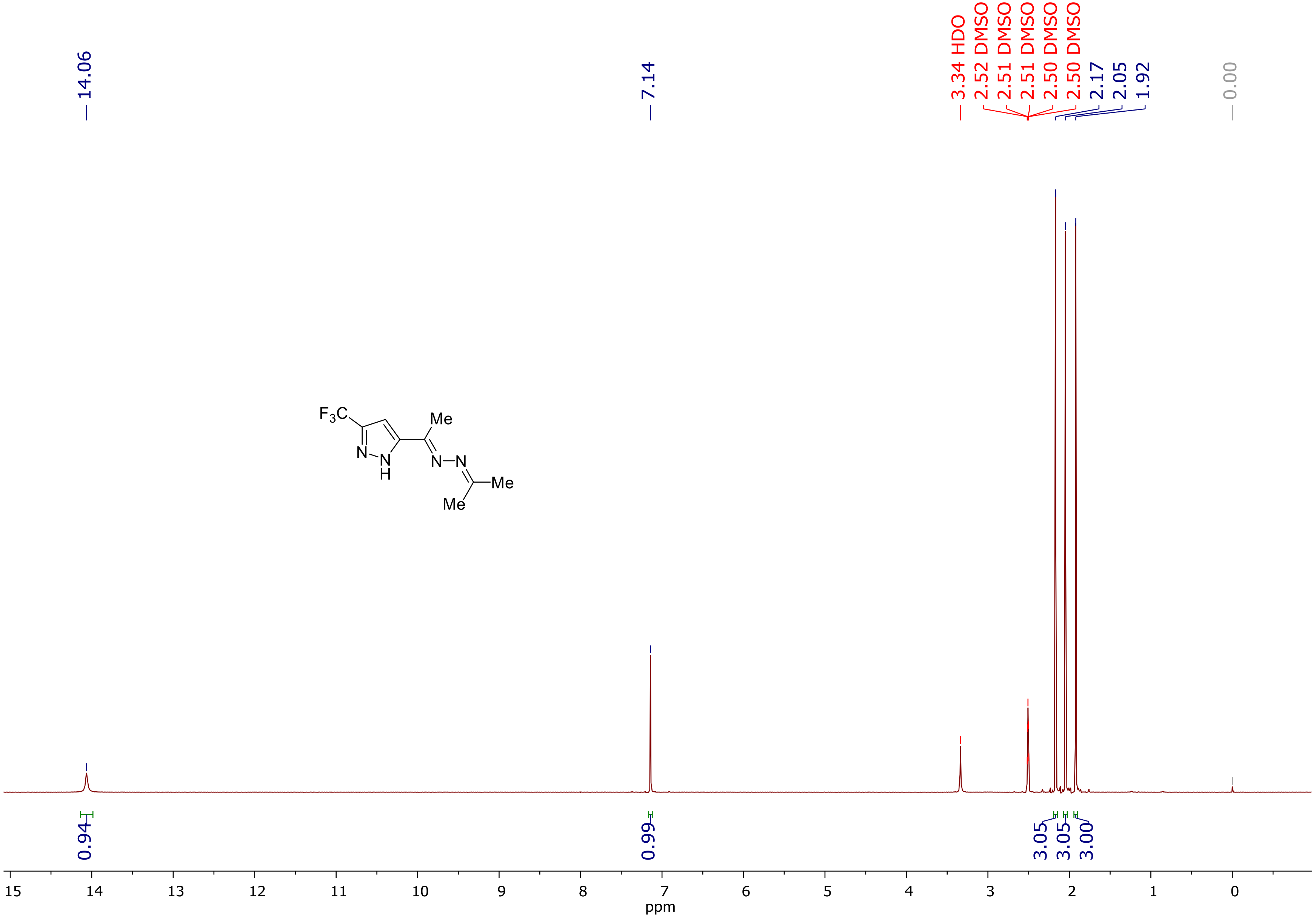

Figure S14. ^{13}C NMR (126 MHz, [D6]DMSO) spectrum of compound **15**

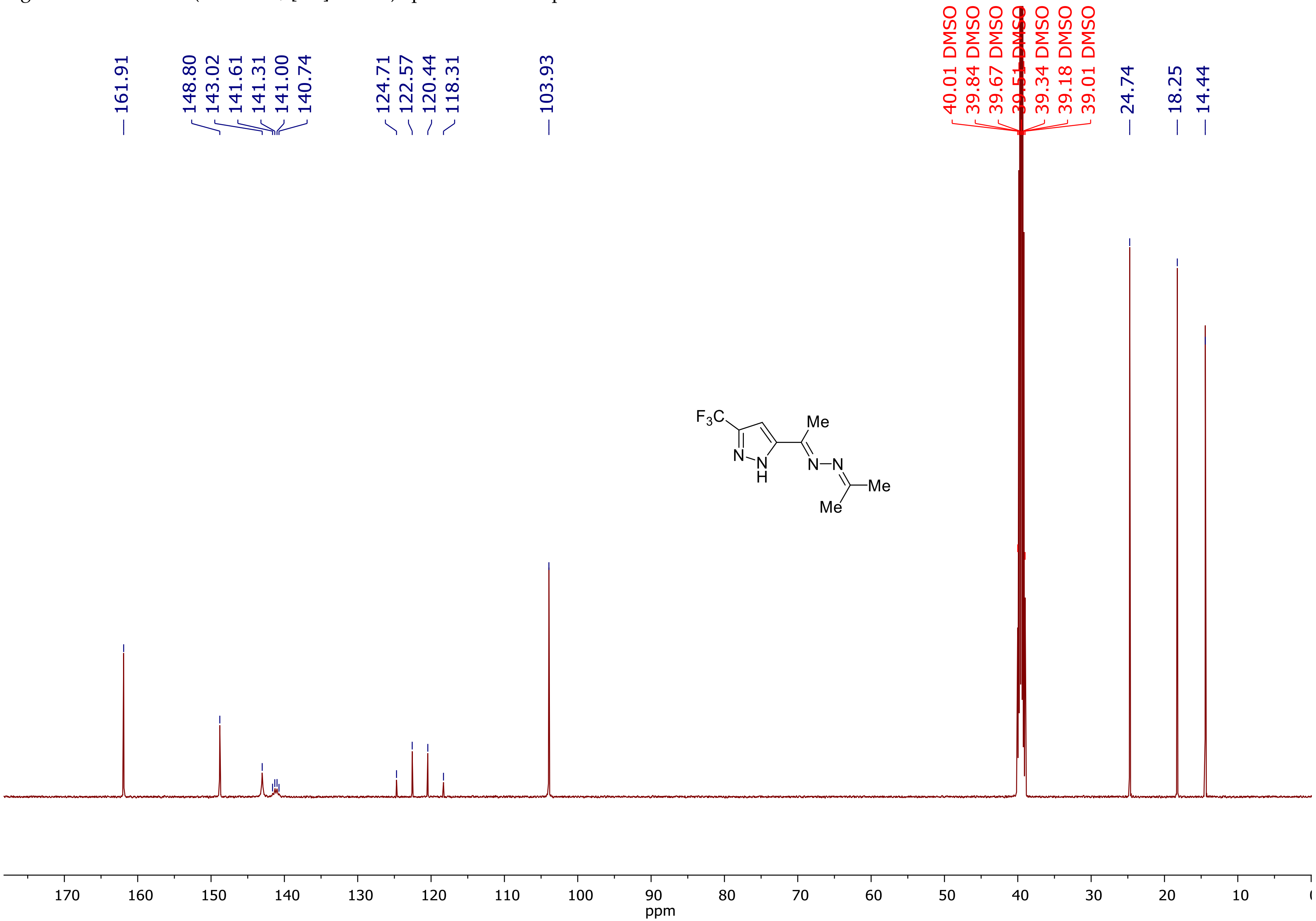

Figure S15. ^{19}F NMR (376 MHz, [D6]DMSO) spectrum of compound **15**

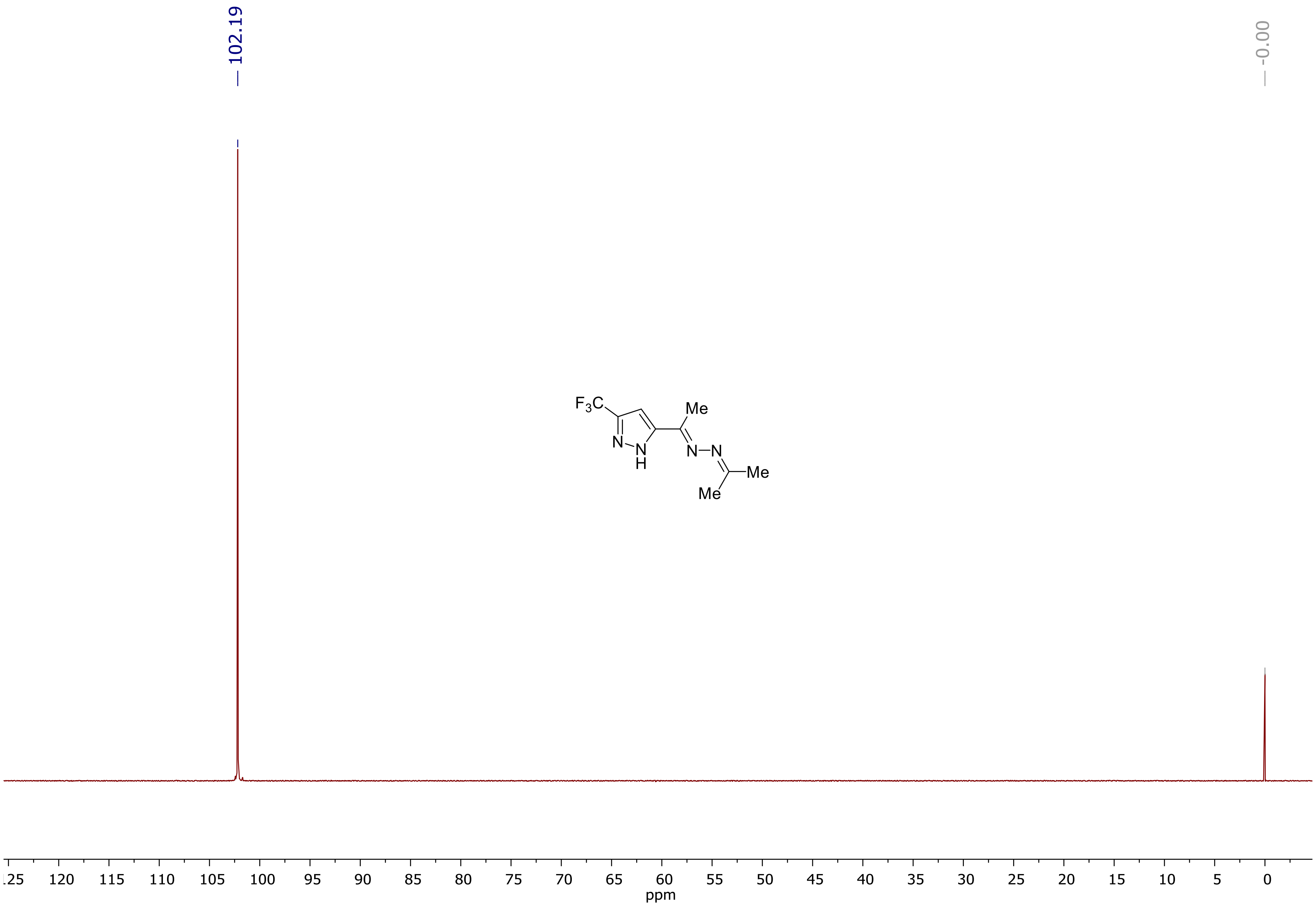

Figure S16. ^{1}H NMR (400 MHz, $CDCl_3$) spectrum of compound **16**

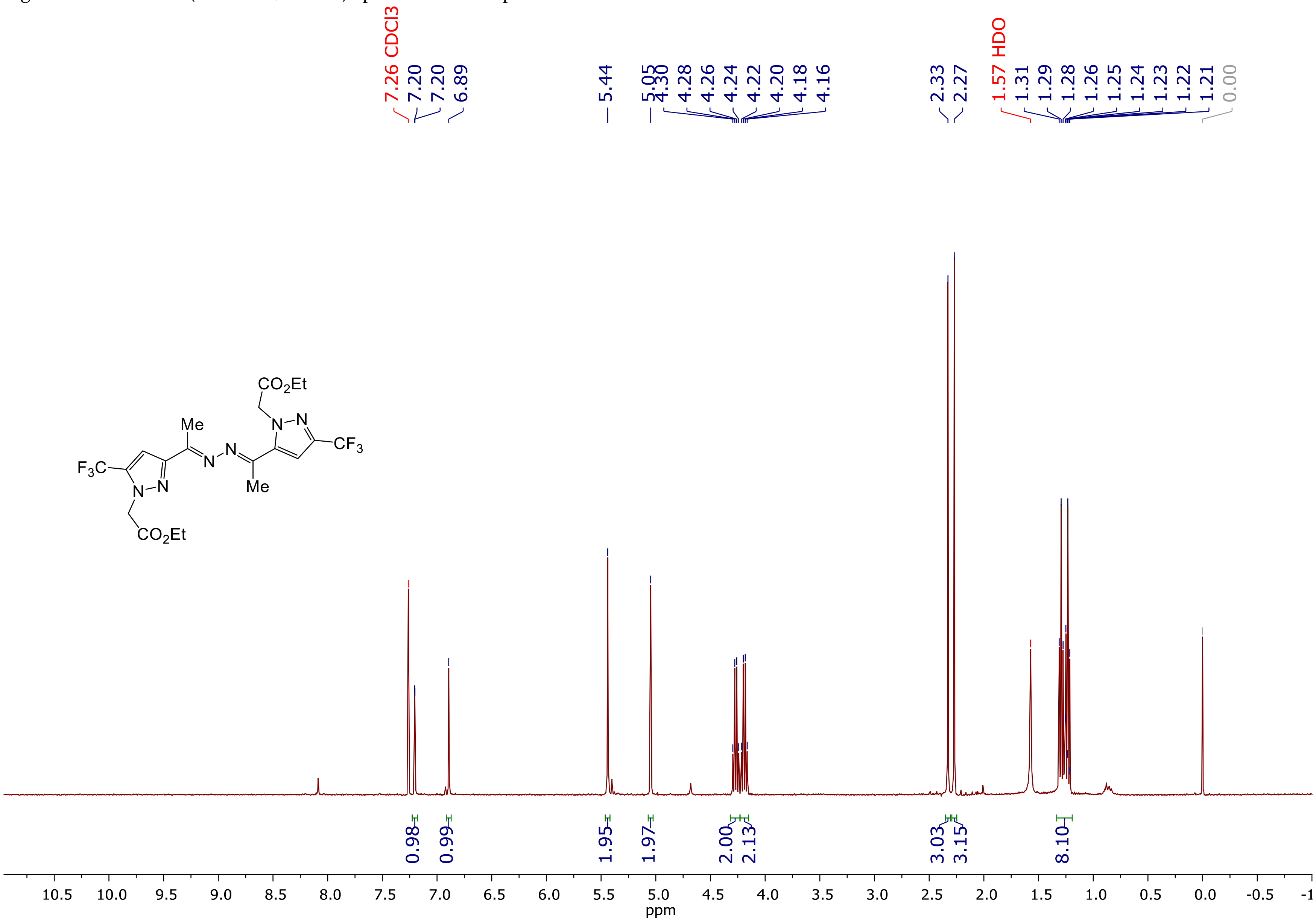

Figure S17. ^{13}C NMR (126 MHz, $CDCl_3$) spectrum of compound **16**

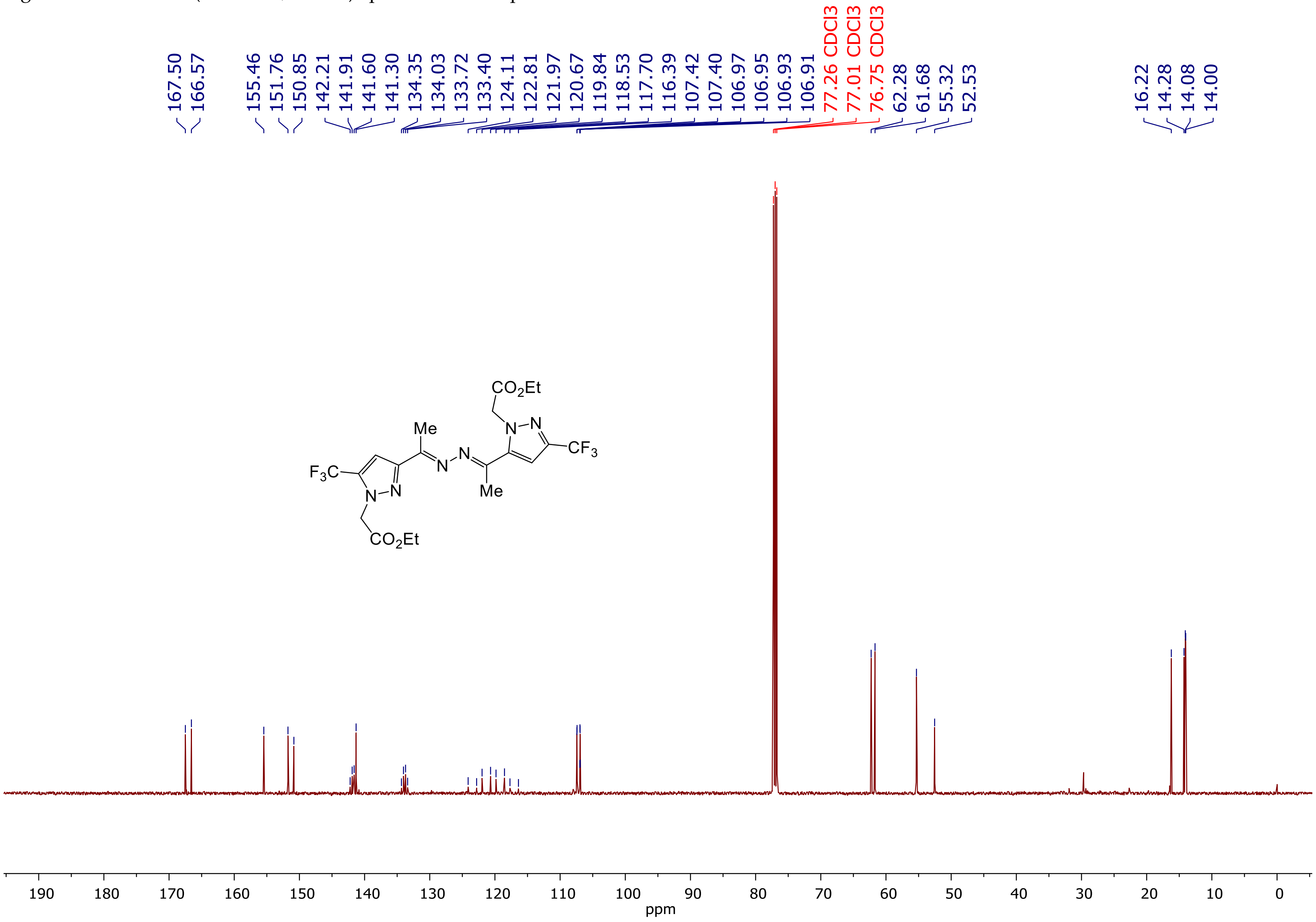

Figure S18. ^{19}F NMR (376 MHz, $CDCl_3$) spectrum of compound **16**

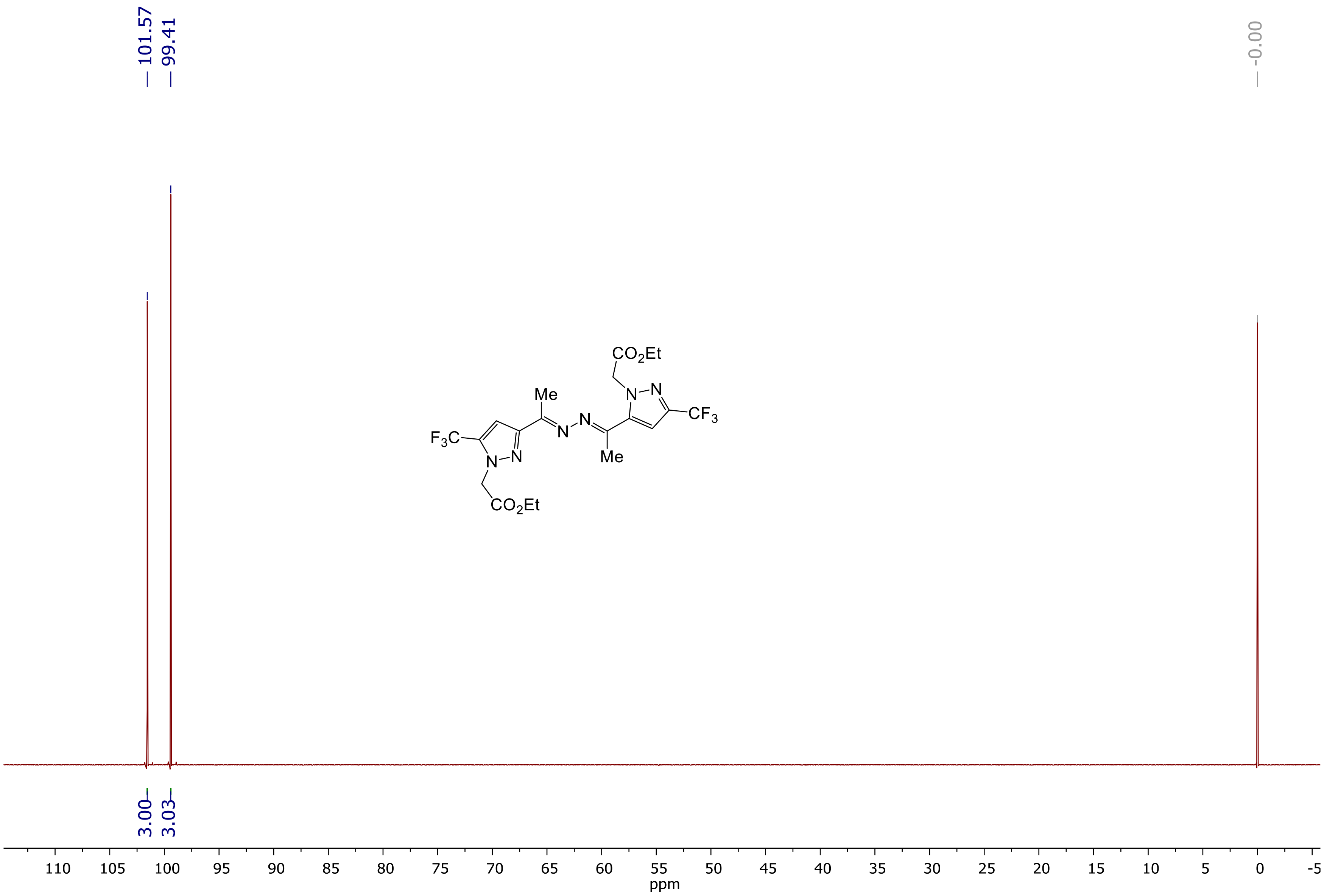

Figure S19. ^{1}H NMR (400 MHz, [D6]DMSO) spectrum of compound **17**

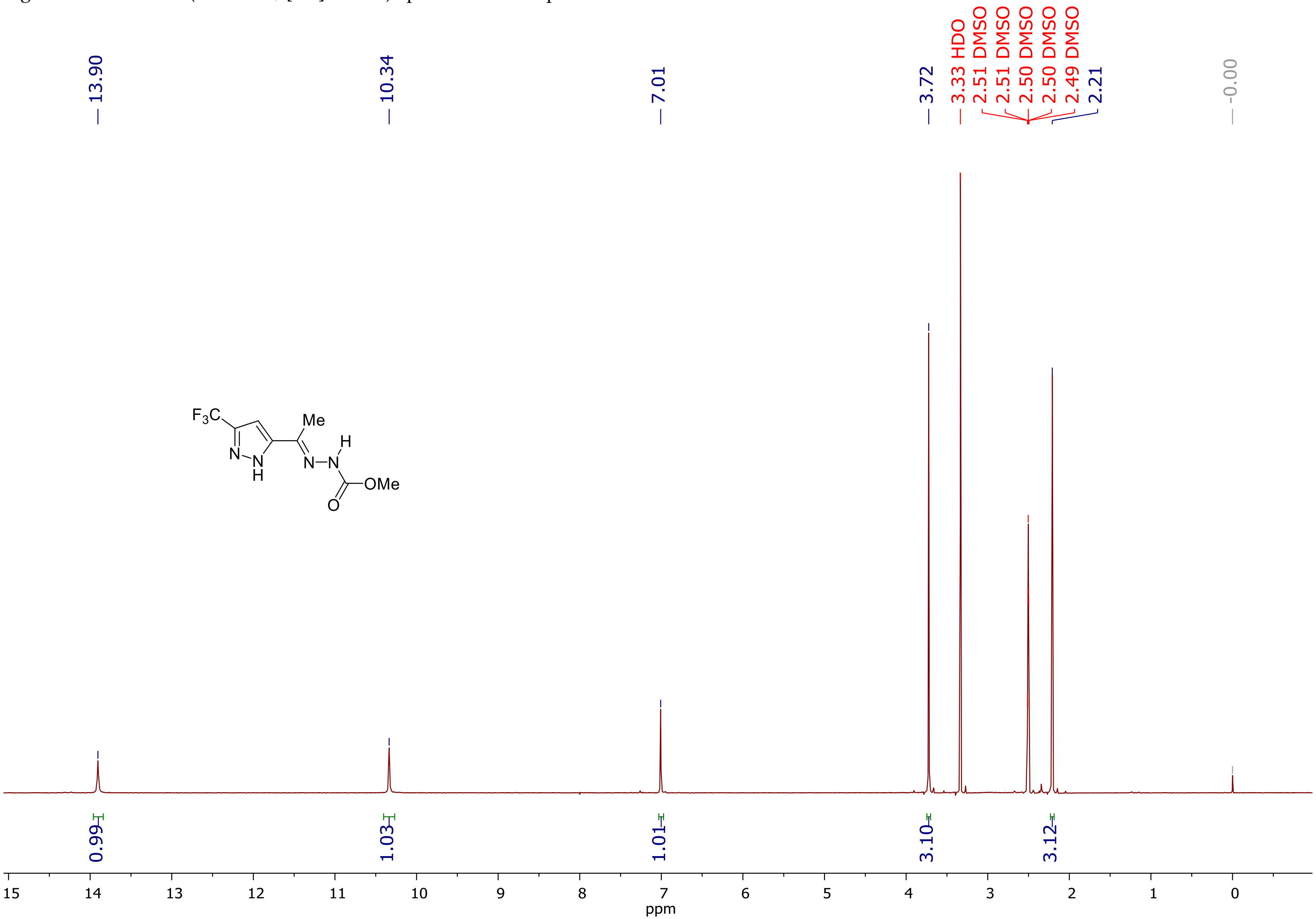

Figure S20. ^{13}C NMR (126 MHz, [D6]DMSO) spectrum of compound **17**

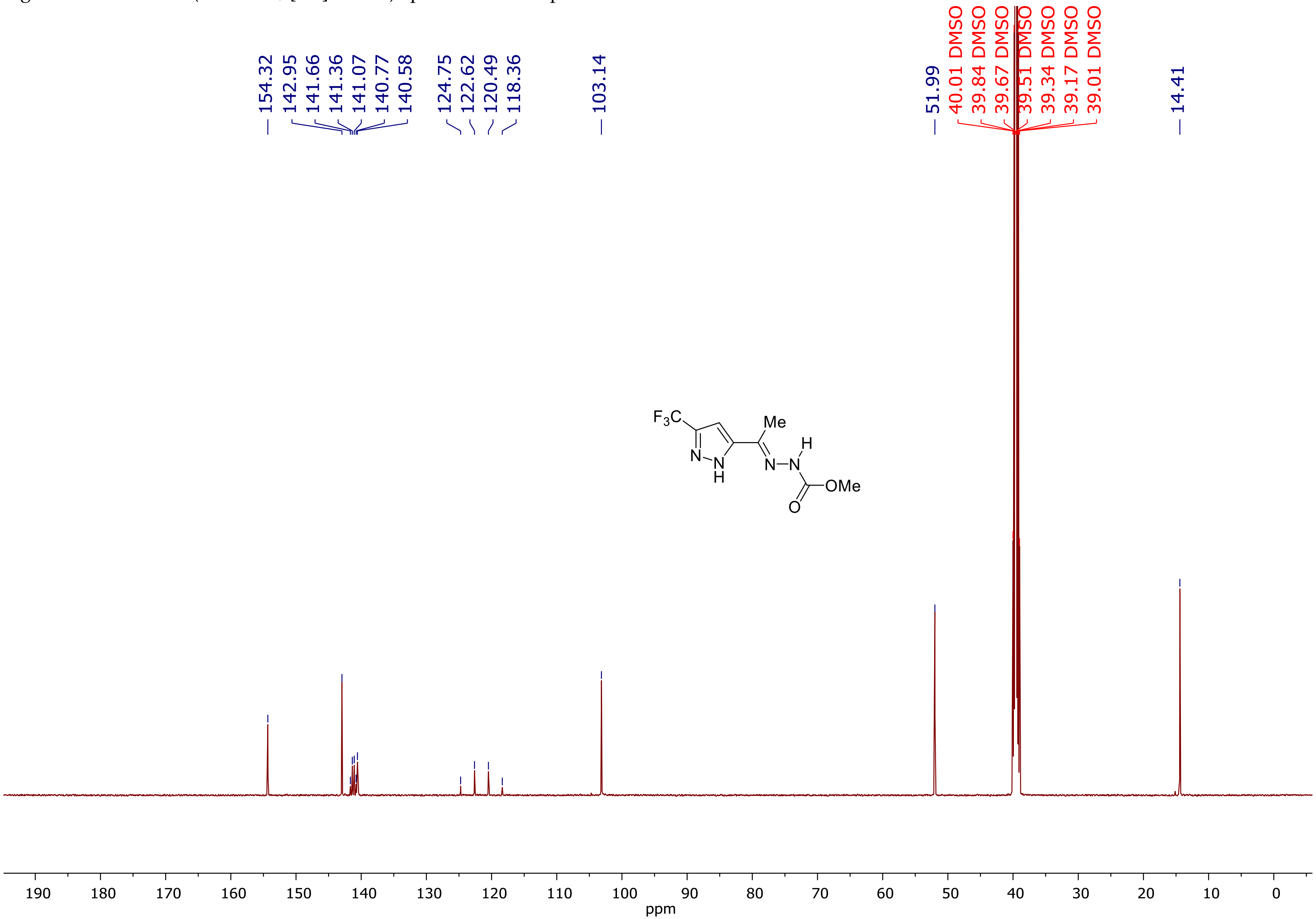

Figure S21. ^{19}F NMR (376 MHz, [D6]DMSO) spectrum of compound **17**

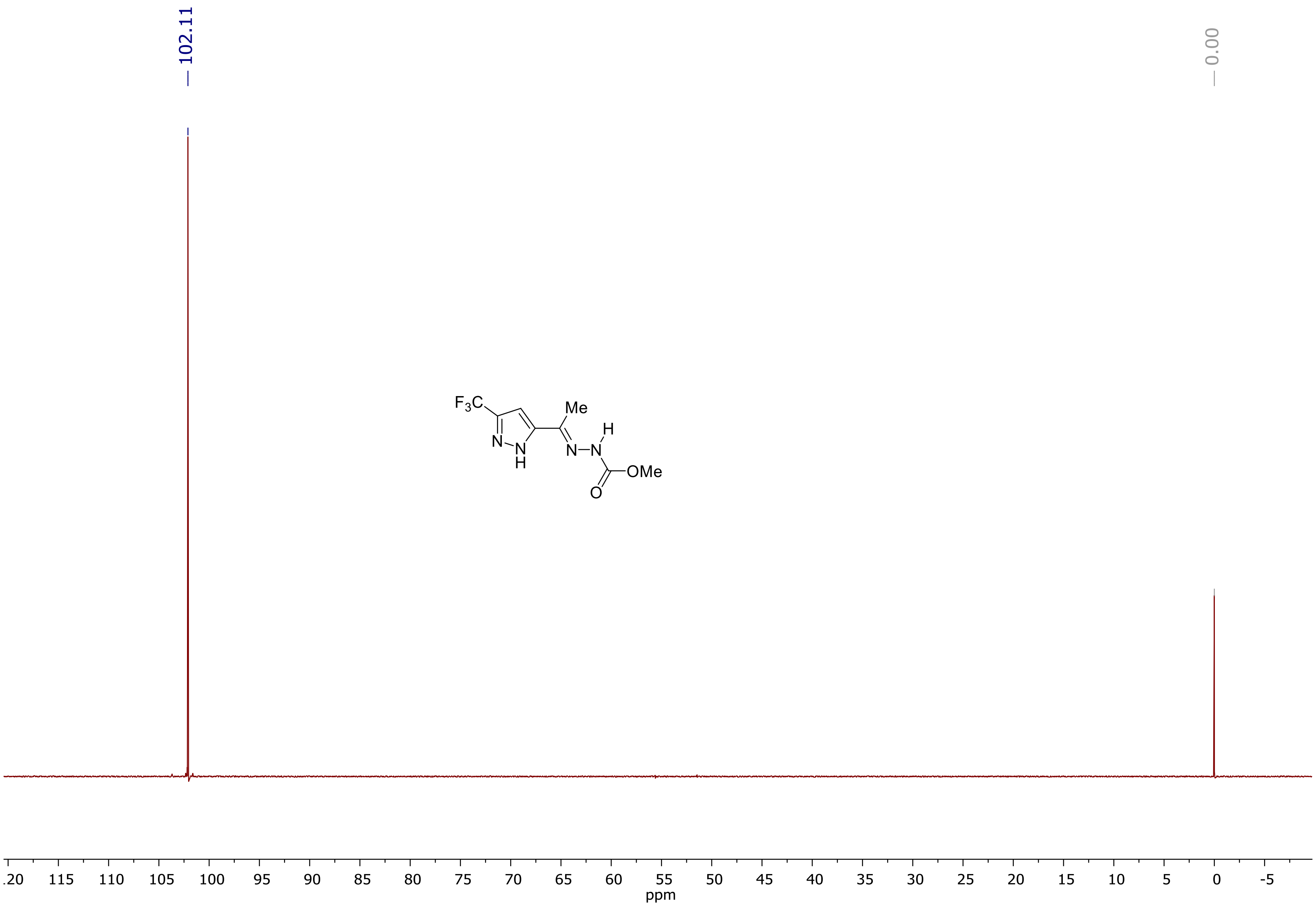

Figure S22. ^{1}H NMR (400 MHz, [D6]DMSO) spectrum of compound **18**

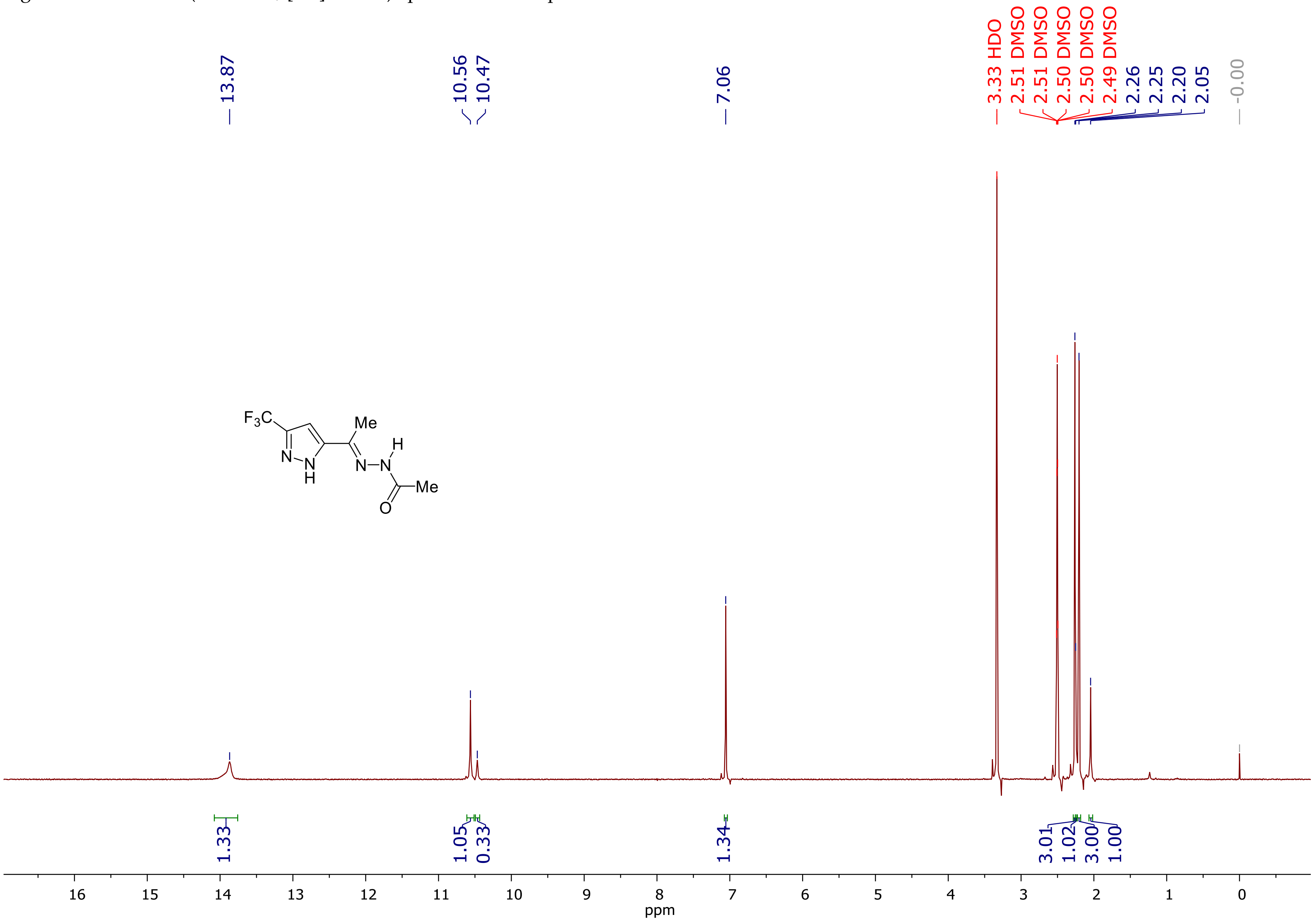

Figure S23. ^{13}C NMR (126 MHz, [D6]DMSO) spectrum of compound **18**

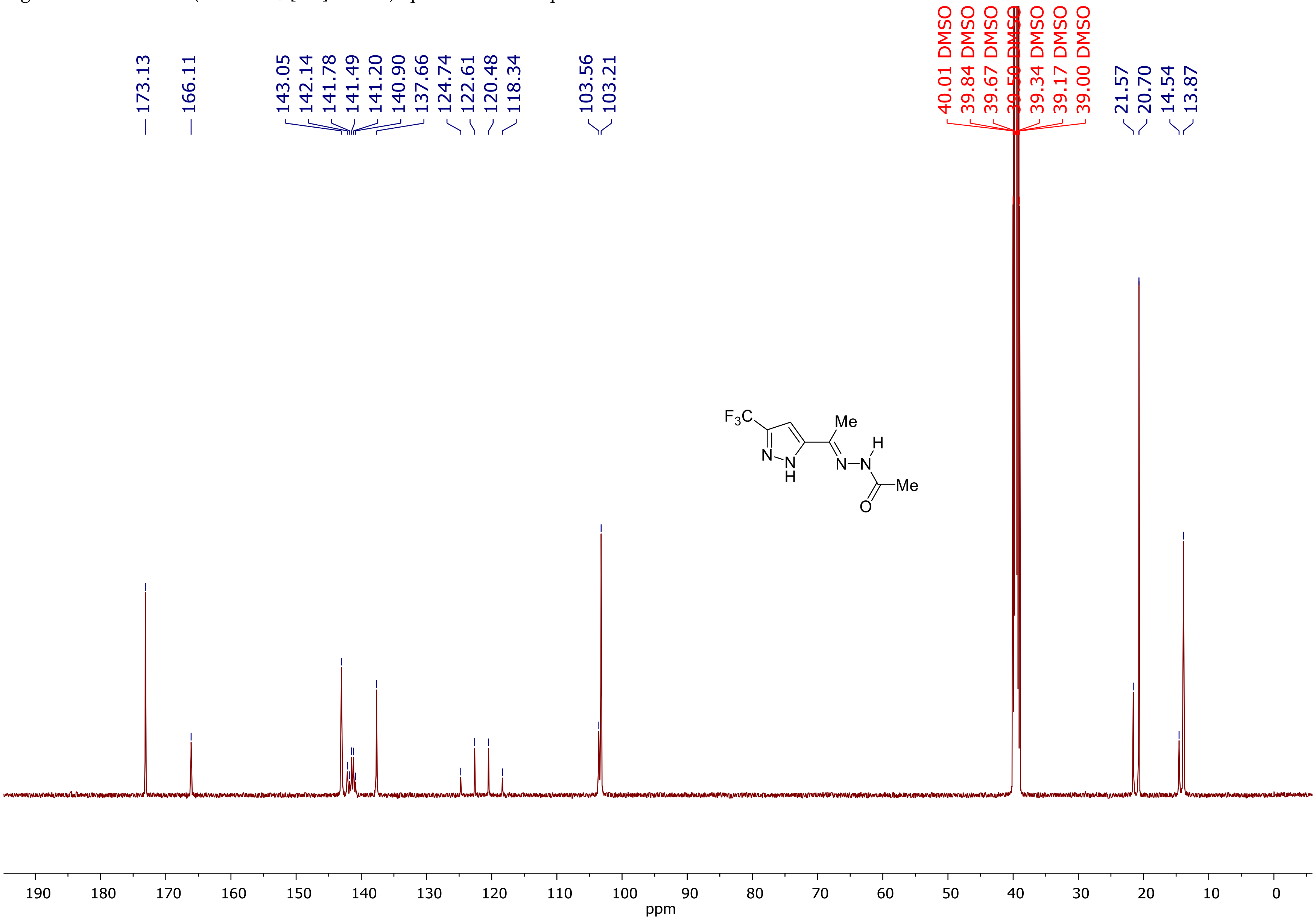

Figure S24. ^{19}F NMR (376 MHz, [D6]DMSO) spectrum of compound **18**

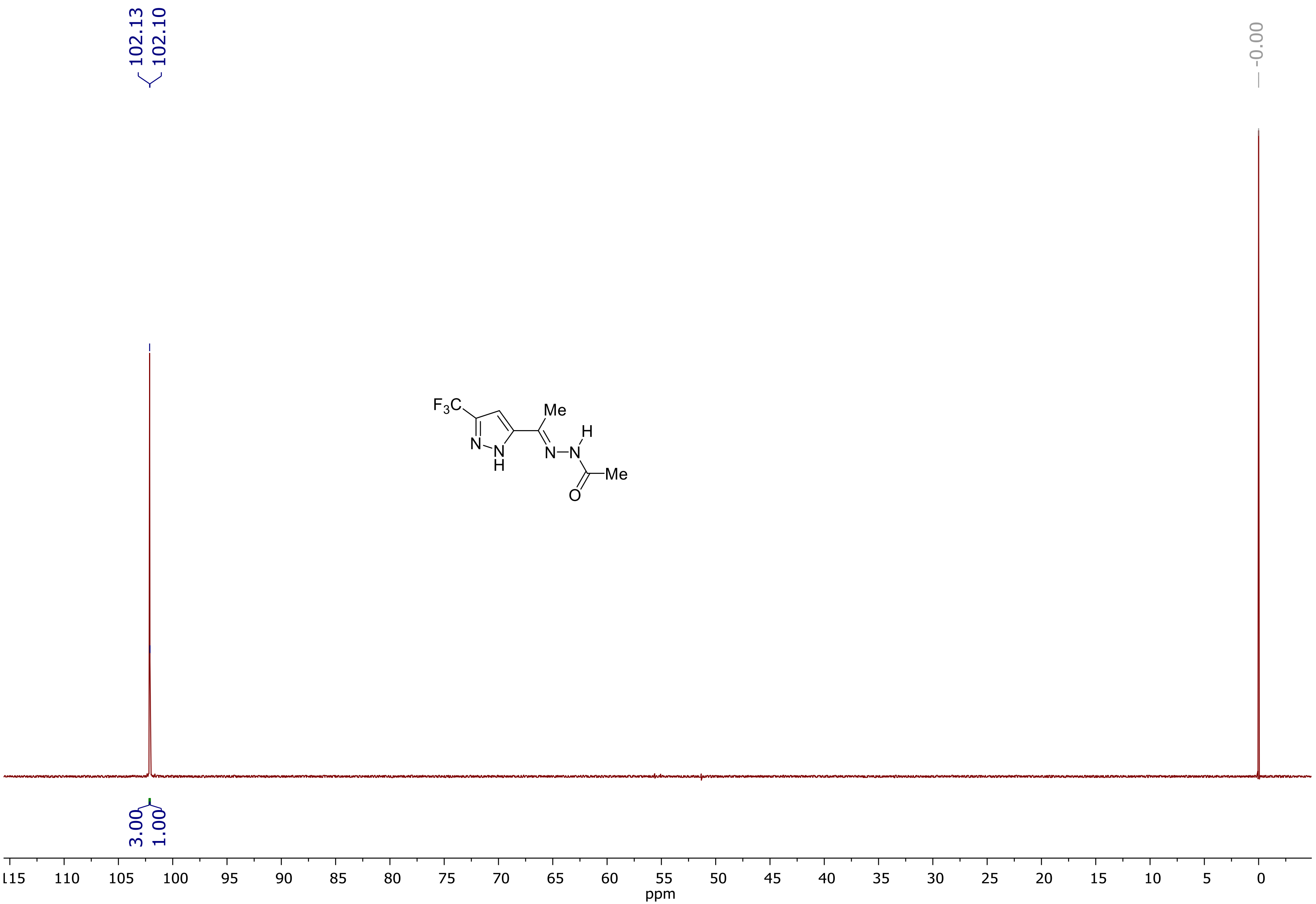

Figure S25. ^{1}H NMR (400 MHz, [D6]DMSO) spectrum of compound **19**

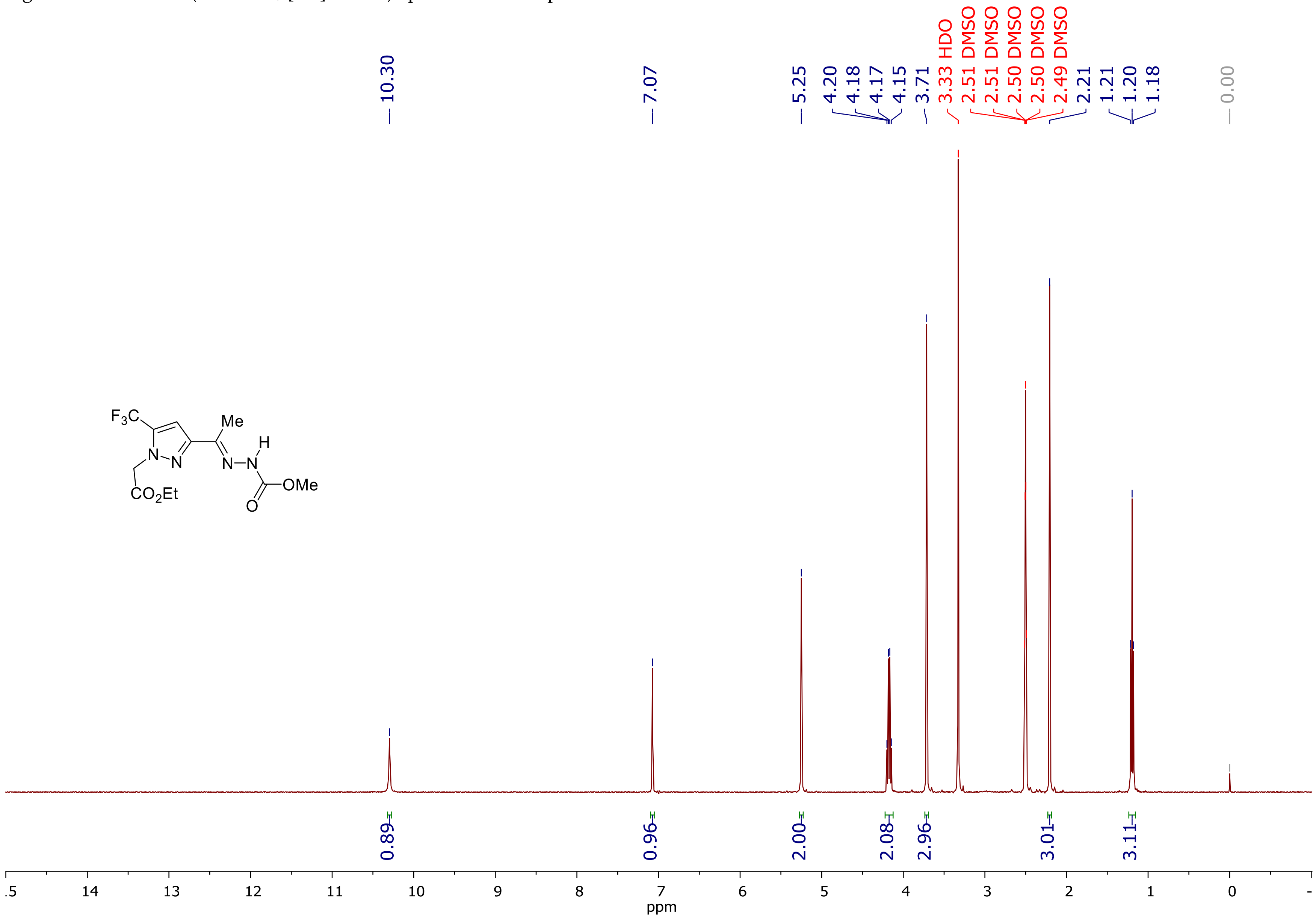

Figure S26. ^{13}C NMR (126 MHz, [D6]DMSO) spectrum of compound **19**

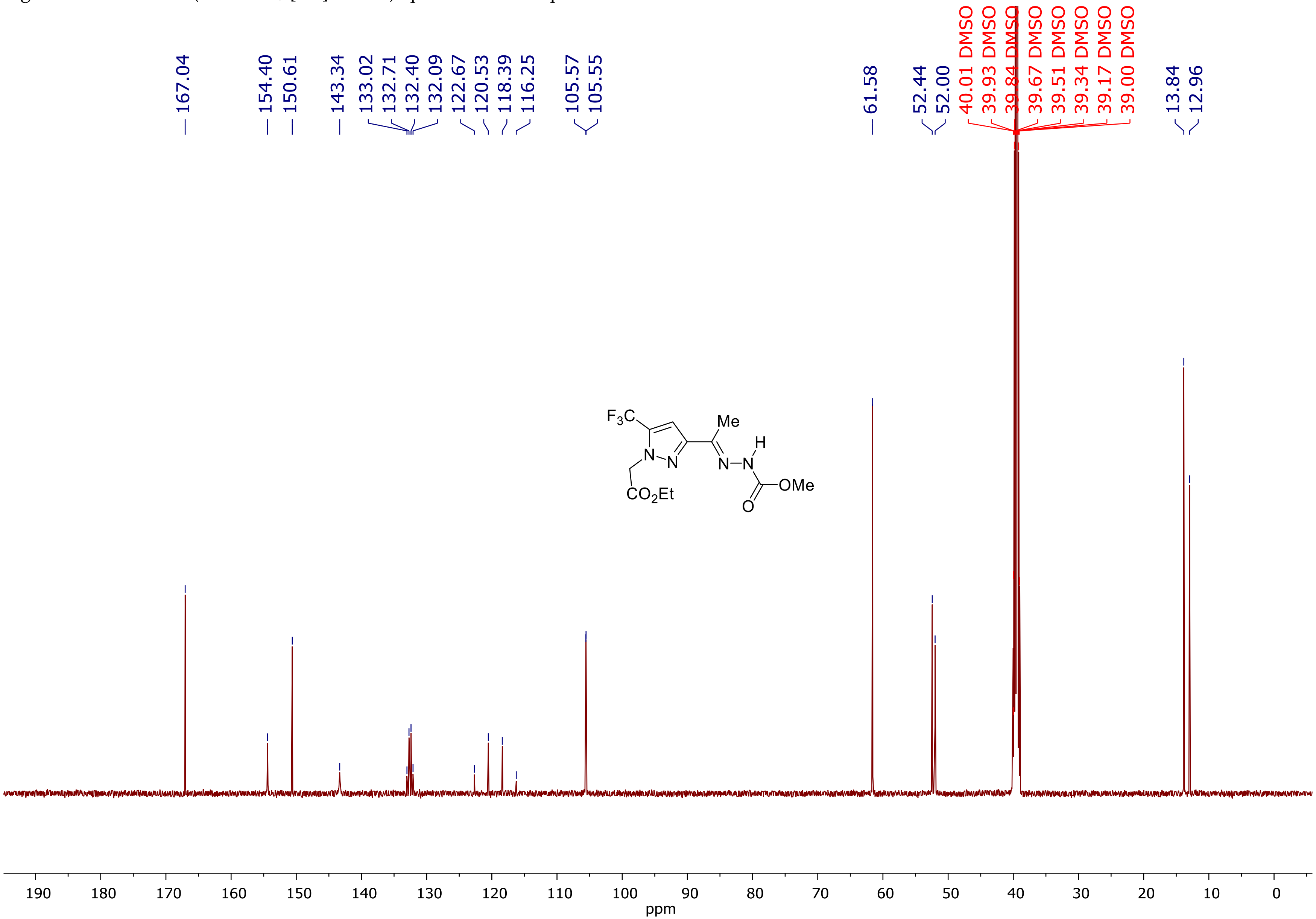

Figure S27. ^{19}F NMR (376 MHz, [D6]DMSO) spectrum of compound **19**

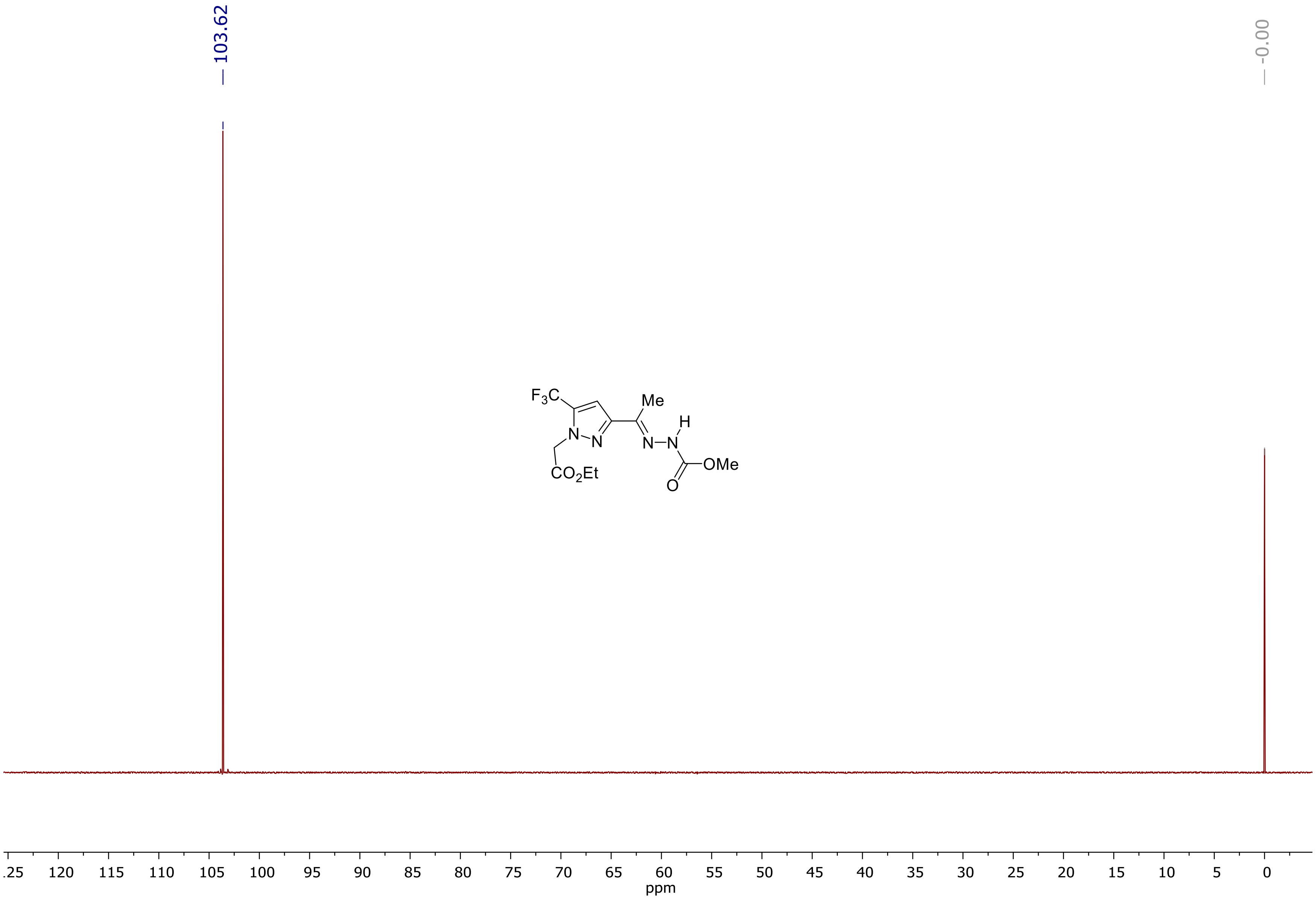

Figure S28. ^{1}H NMR (400 MHz, [D6]DMSO) spectrum of compound **20**

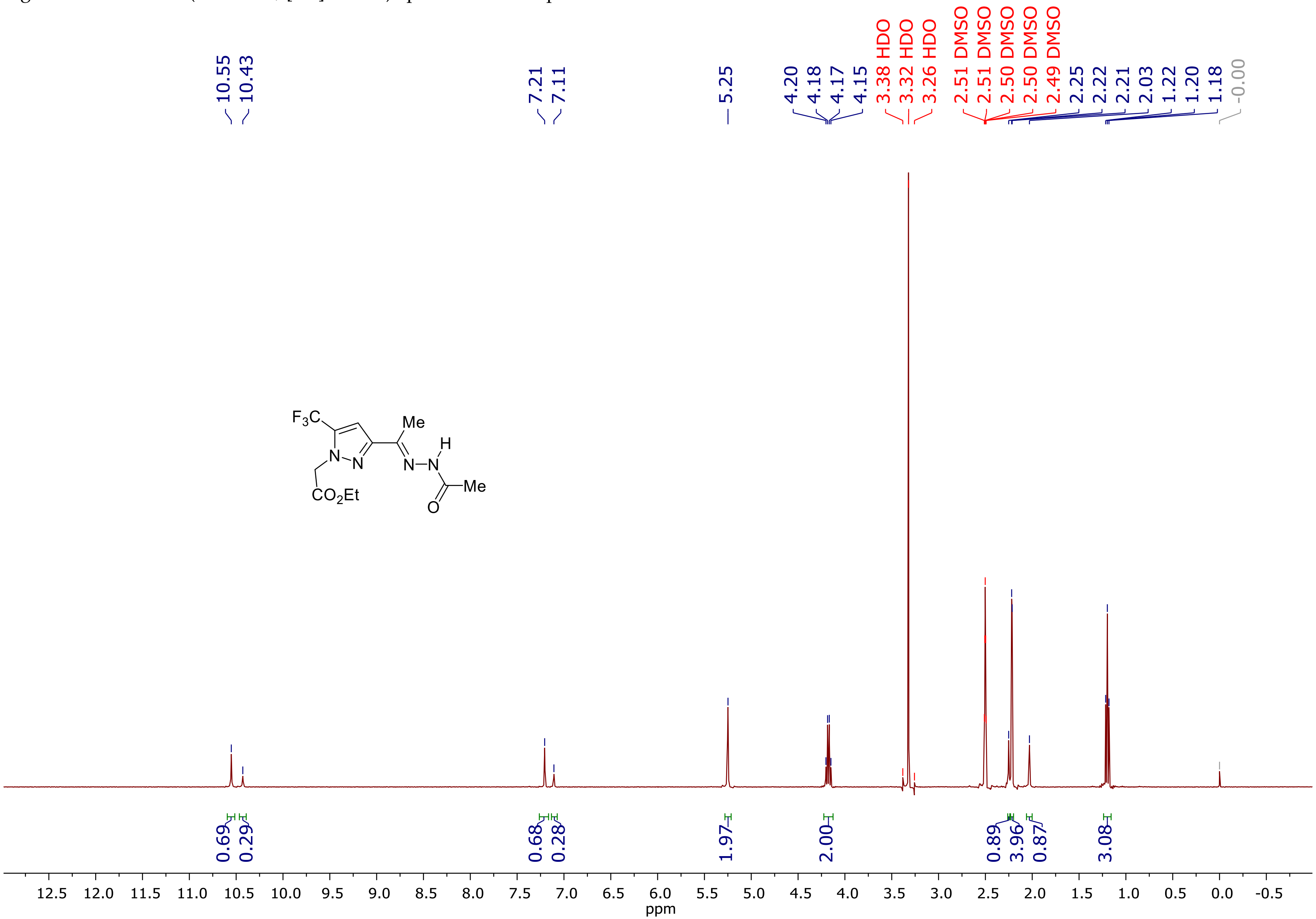

Figure S29. ^{13}C NMR (126 MHz, [D6]DMSO) spectrum of compound **20**

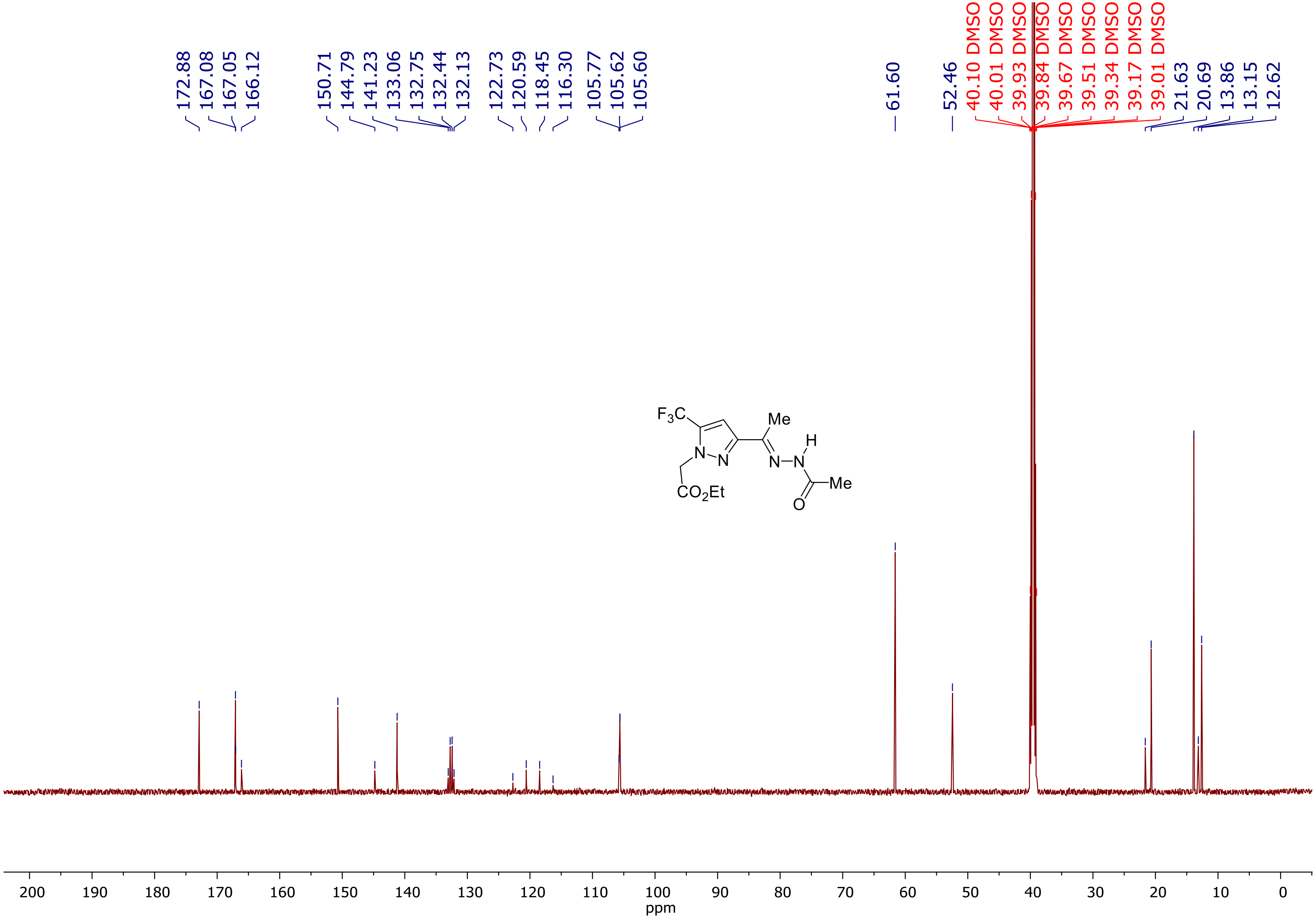

Figure S30. ^{19}F NMR (376 MHz, [D6]DMSO) spectrum of compound **20**

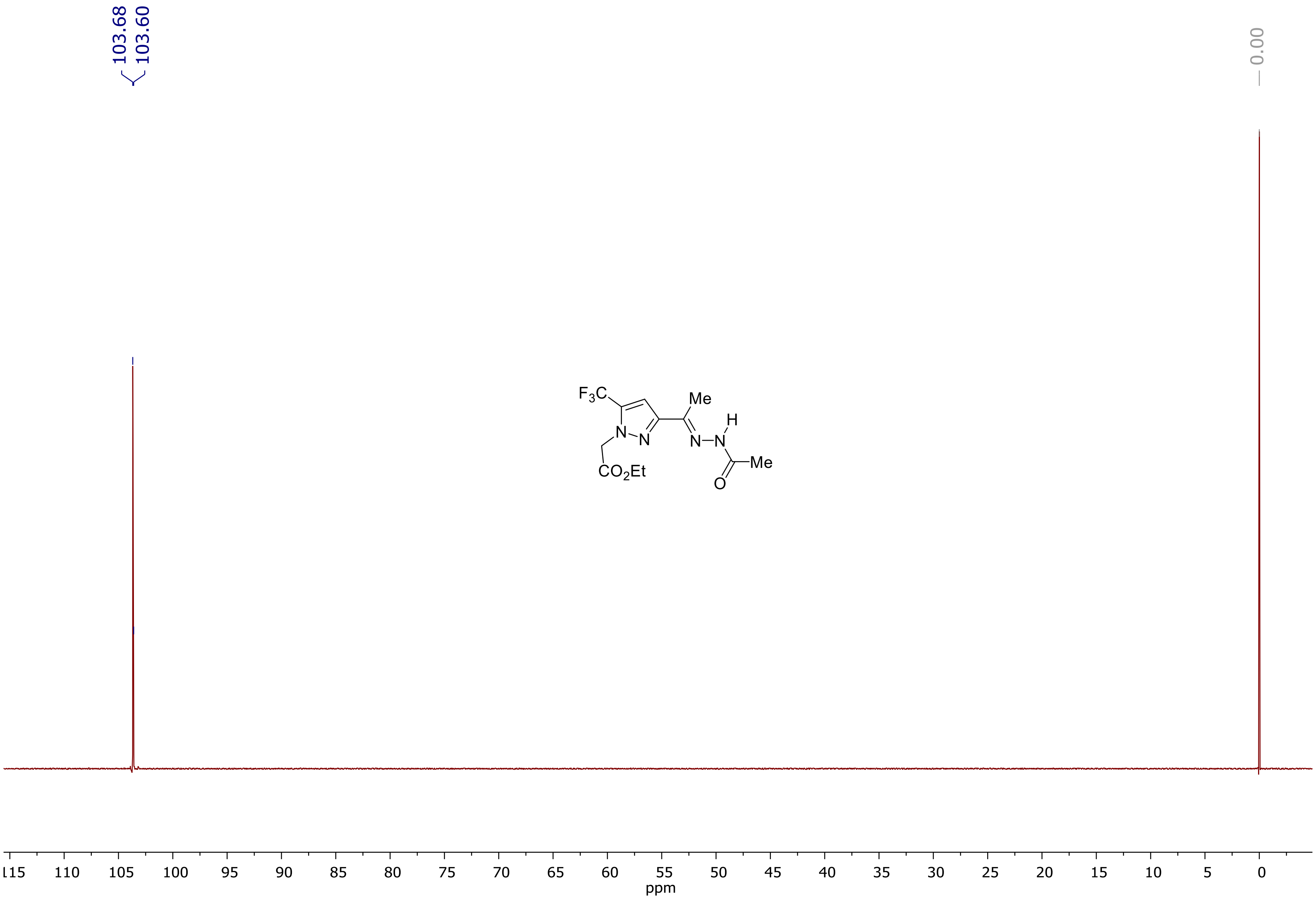

Figure S31. ^{1}H NMR (500 MHz, [D6]DMSO) spectrum of compound **22**

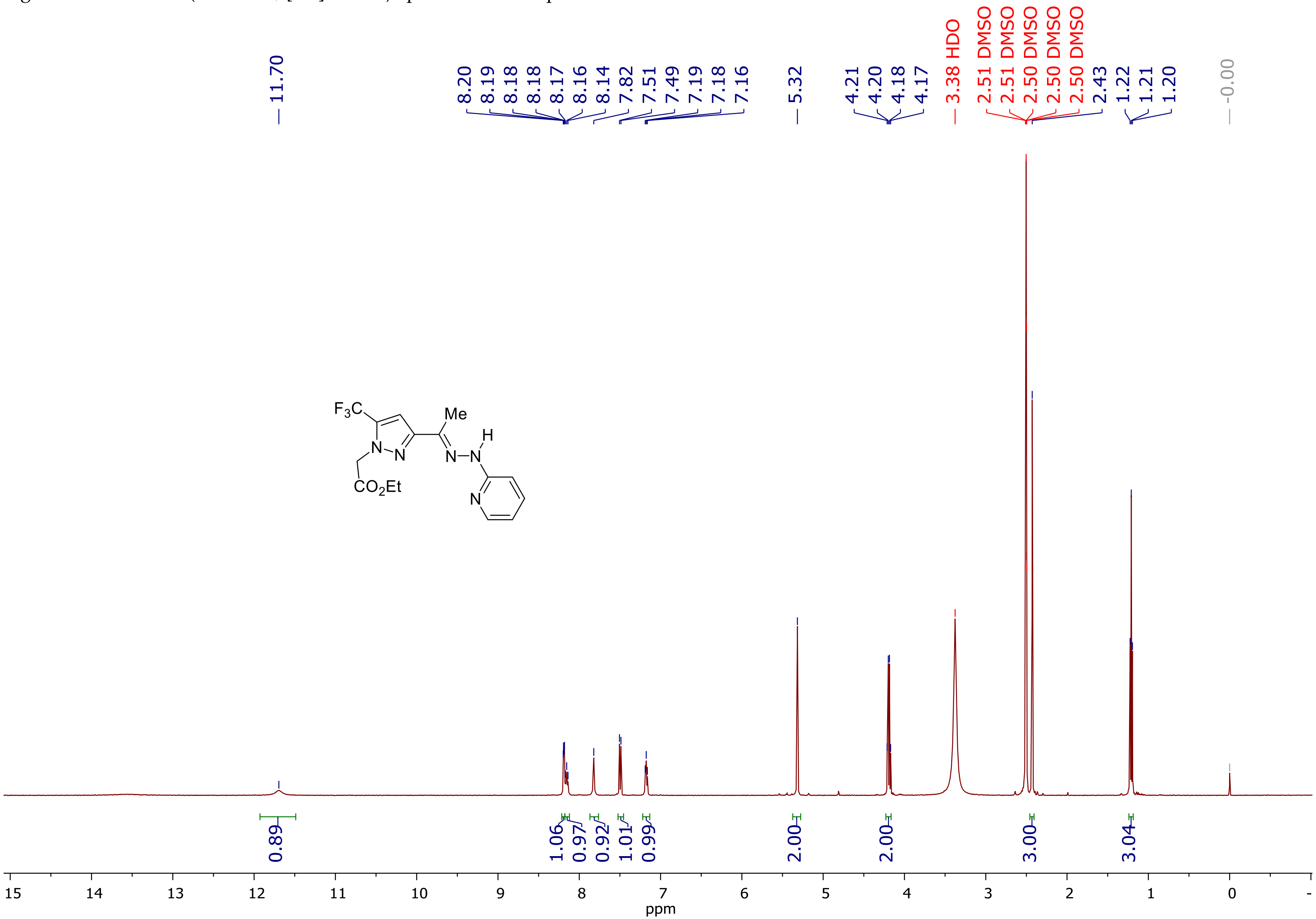

Figure S32. ^{13}C NMR (126 MHz, [D6]DMSO) spectrum of compound **22**

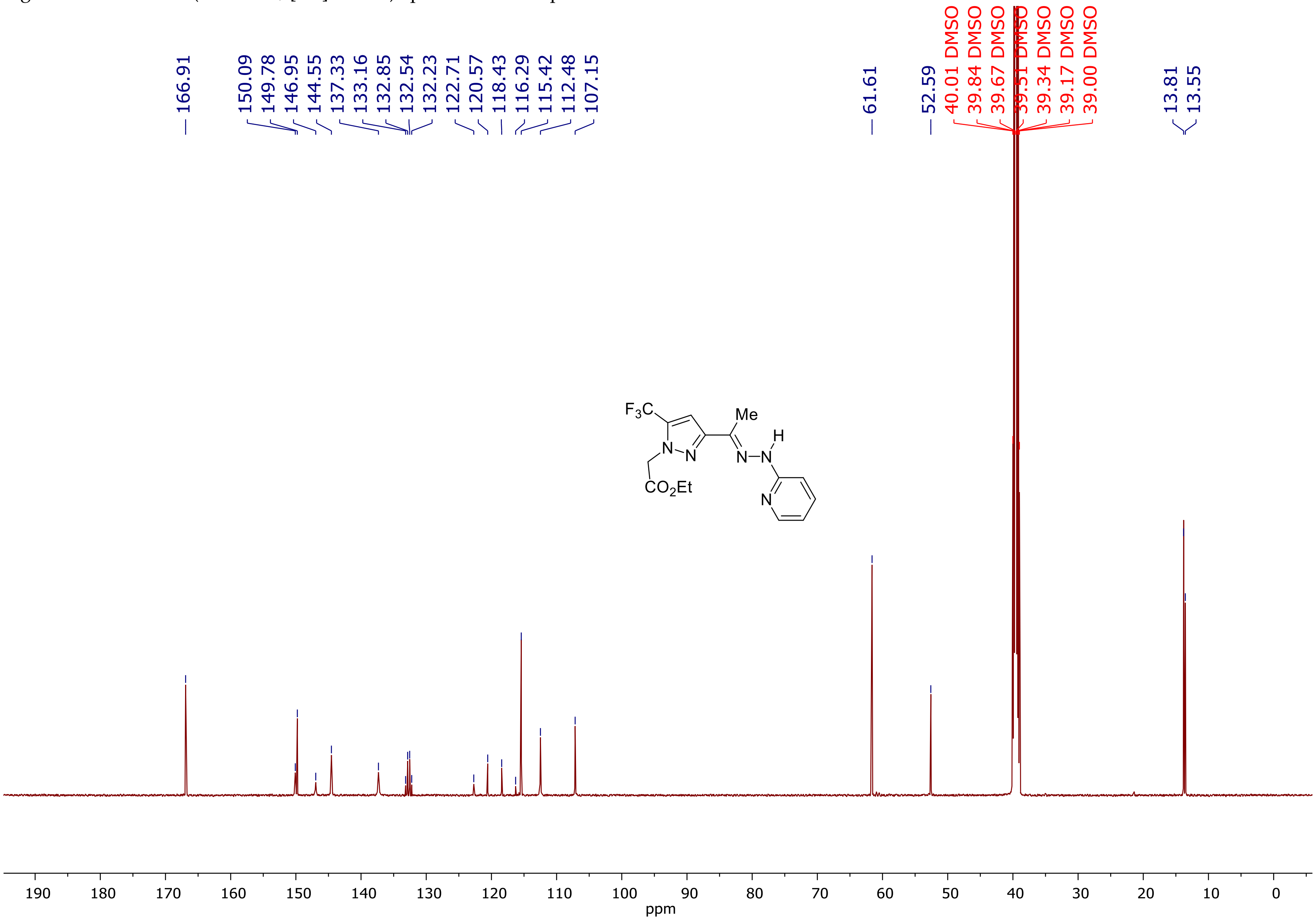

Figure S33. ^{19}F NMR (470 MHz, [D6]DMSO) spectrum of compound **22**

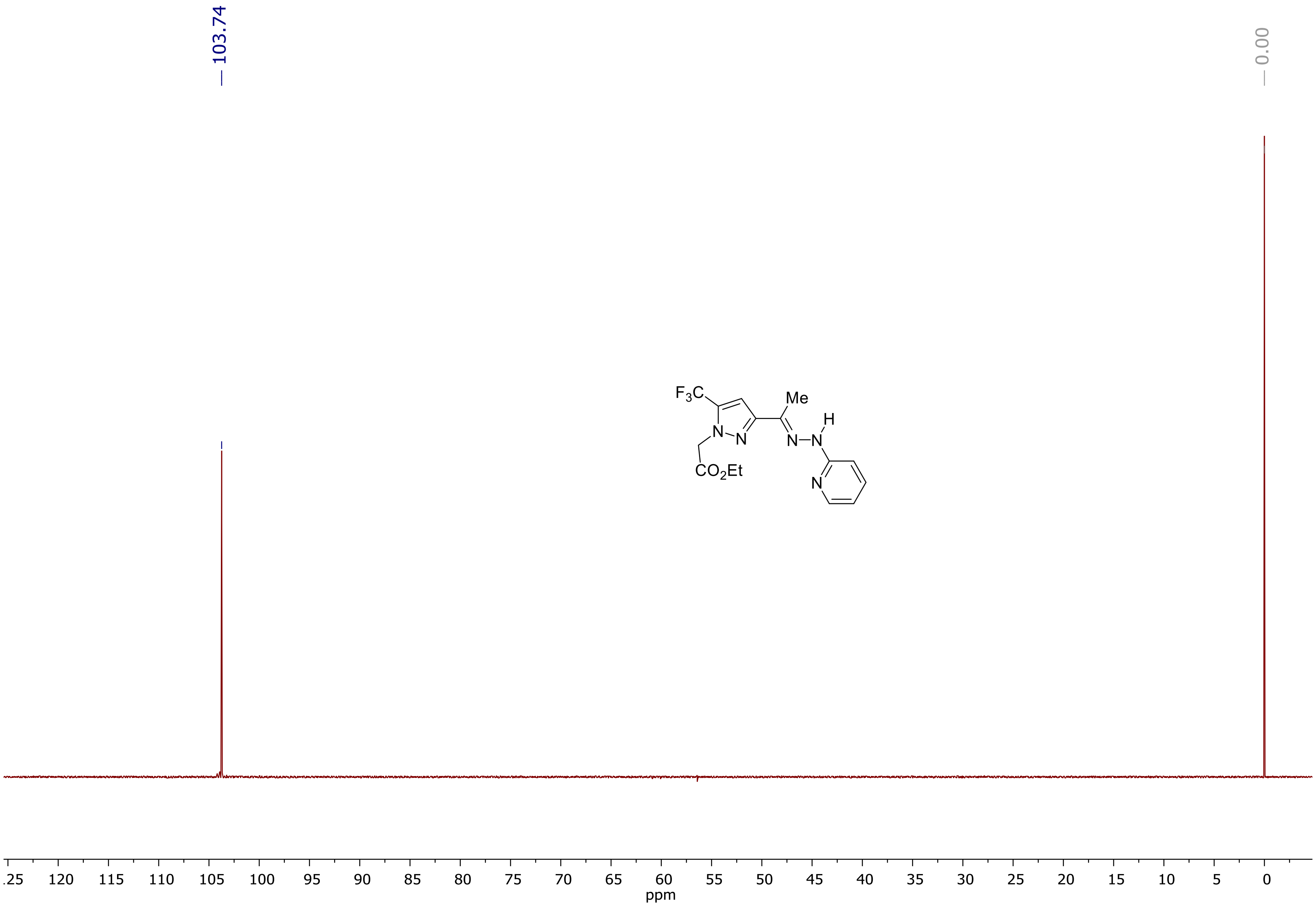

Figure S34. ¹H NMR (400 MHz, [D6]DMSO) spectrum of compound **23**

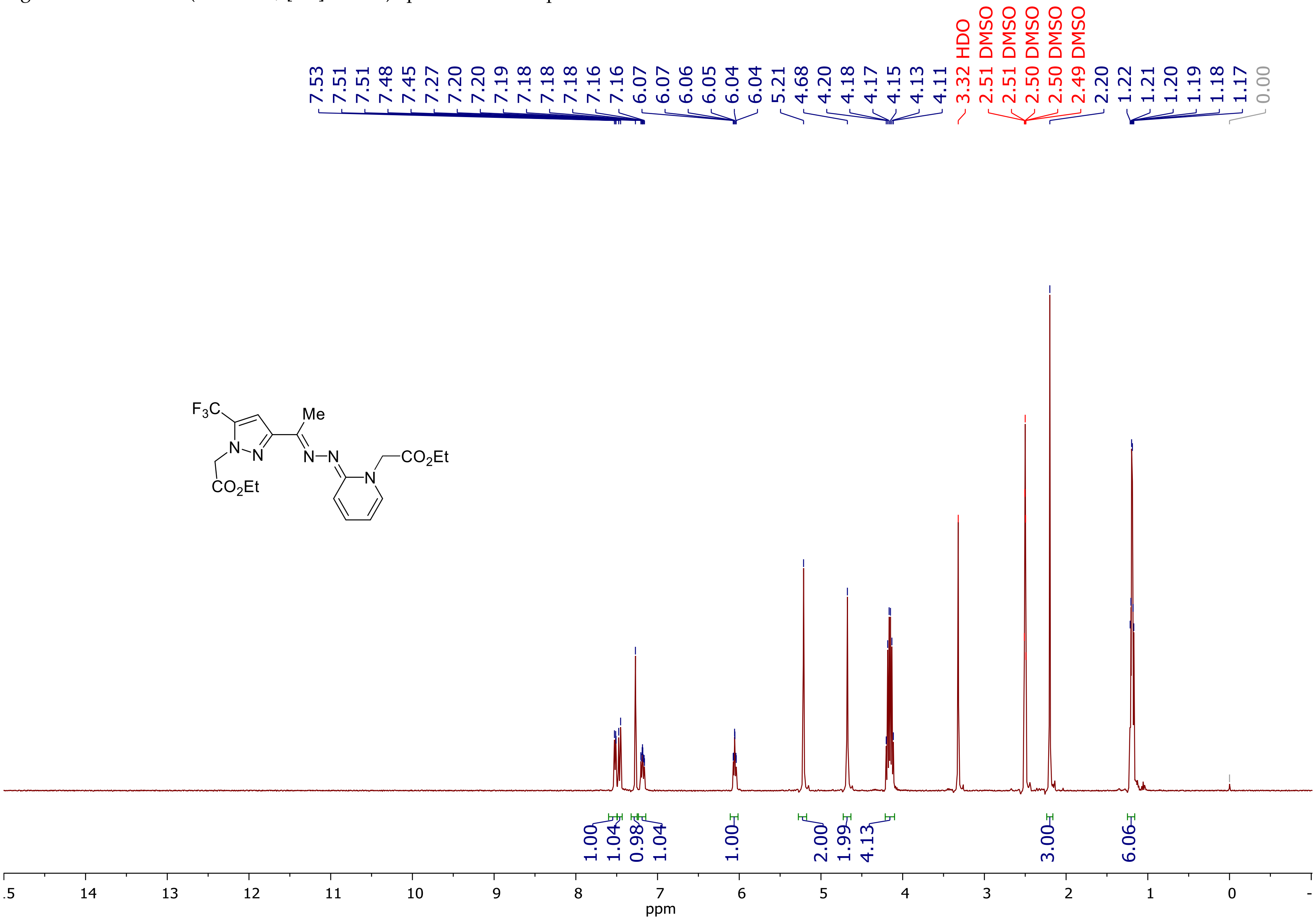

Figure S35. ^{13}C NMR (126 MHz, [D6]DMSO) spectrum of compound **23**

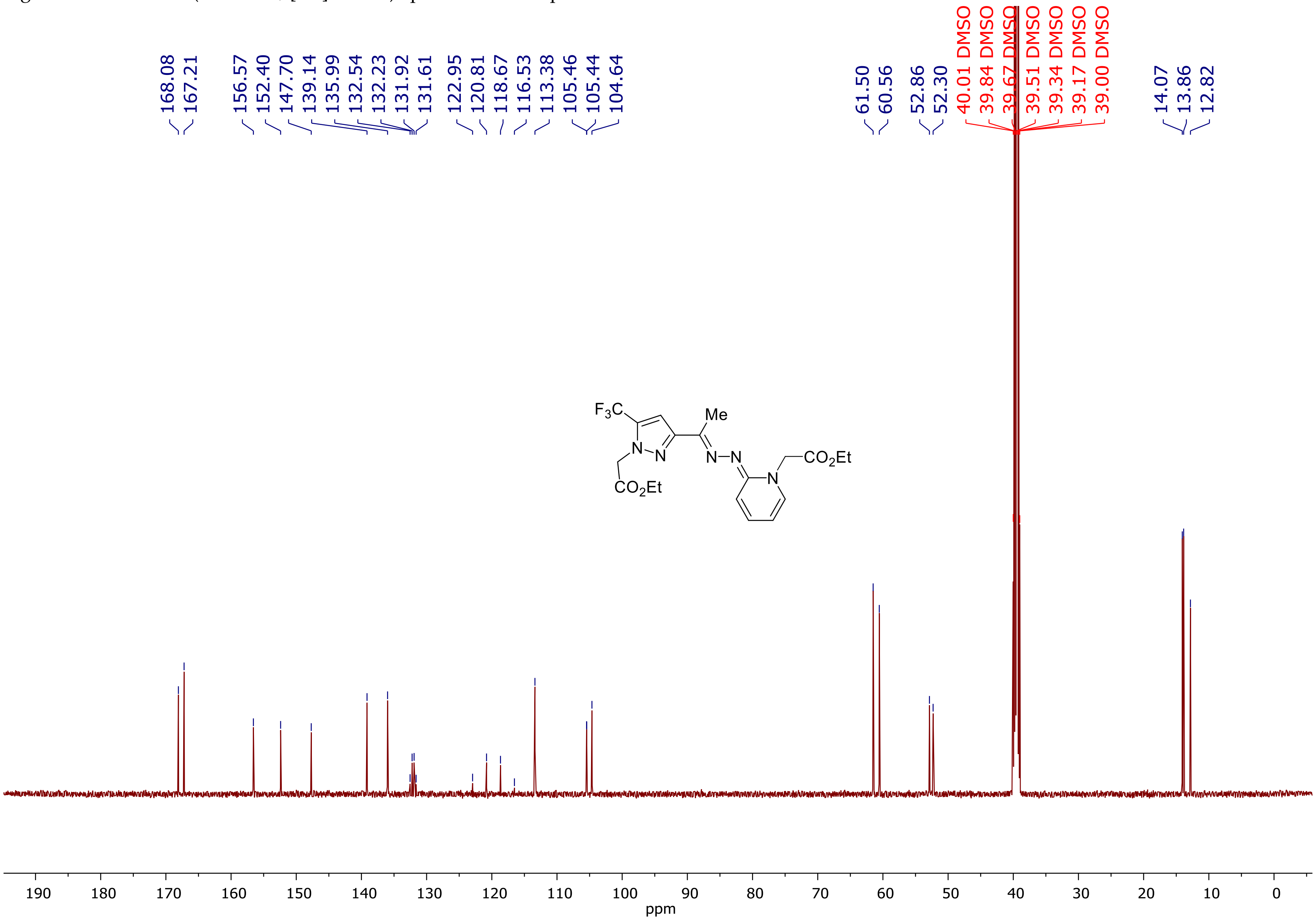

Figure S36. ^{19}F NMR (376 MHz, [D6]DMSO) spectrum of compound **23**

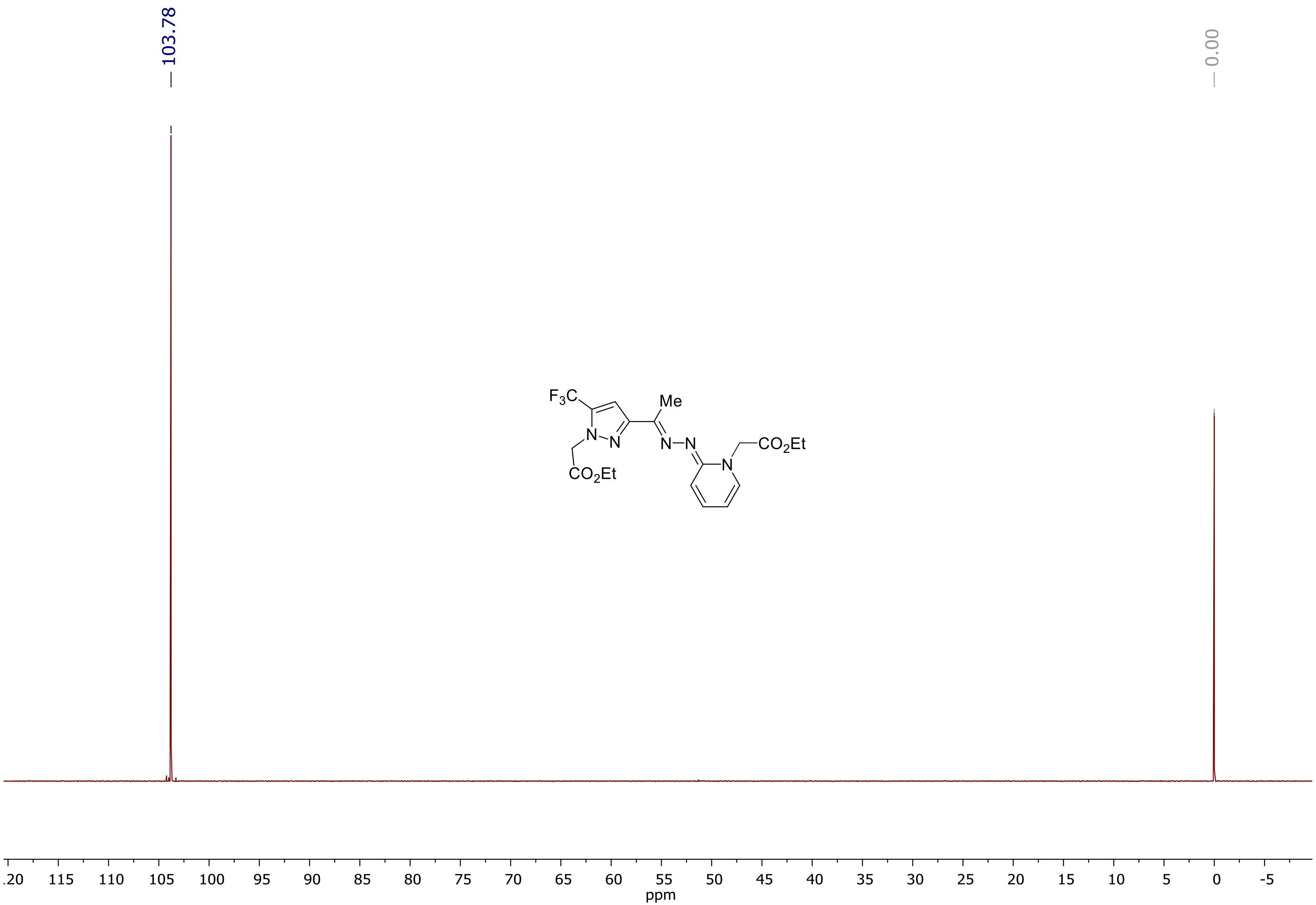

Figure S37. ^{1}H NMR (400 MHz, [D6]DMSO) spectrum of compound **24**

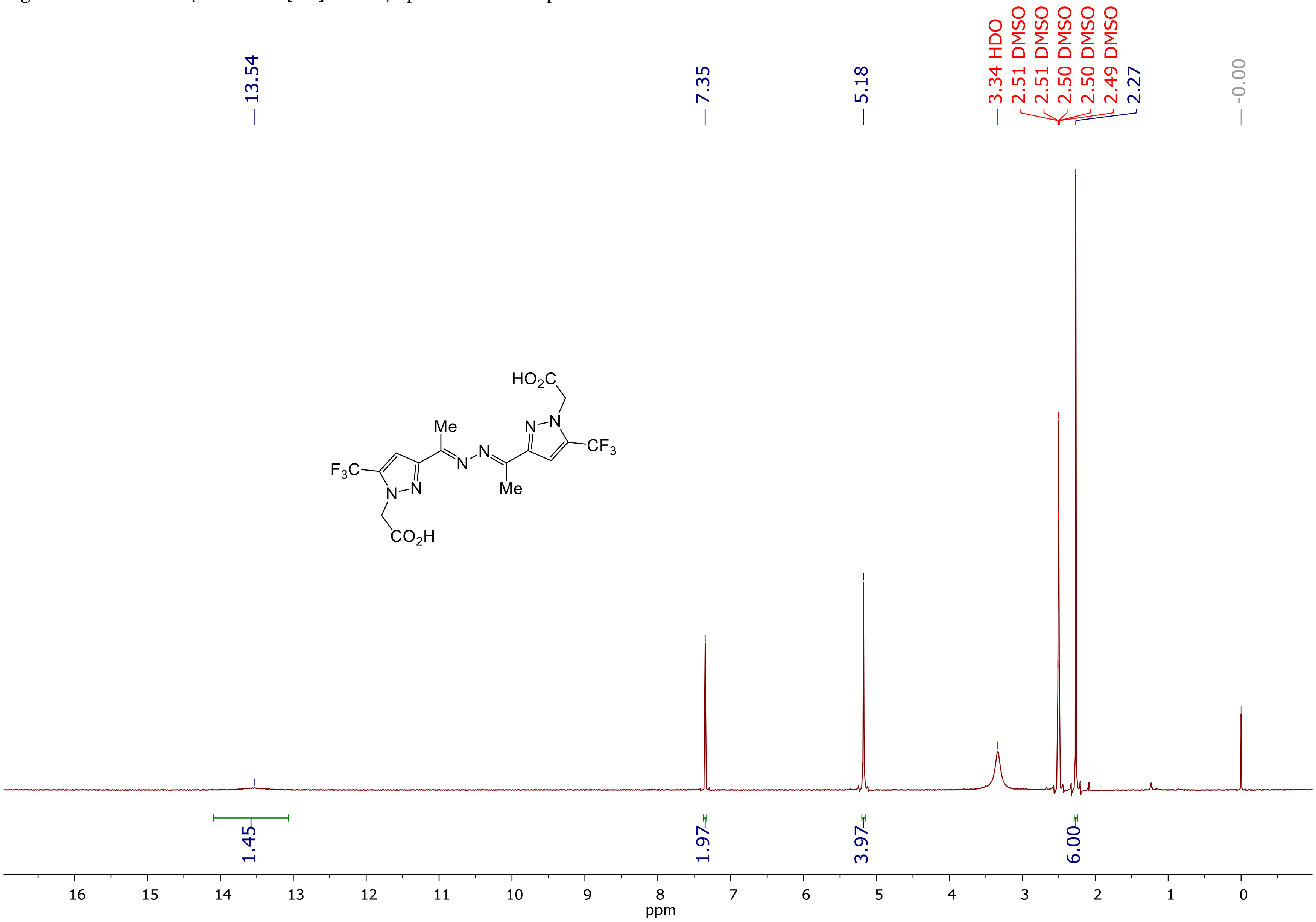

Figure S38. ^{13}C NMR (126 MHz, [D6]DMSO) spectrum of compound **24**

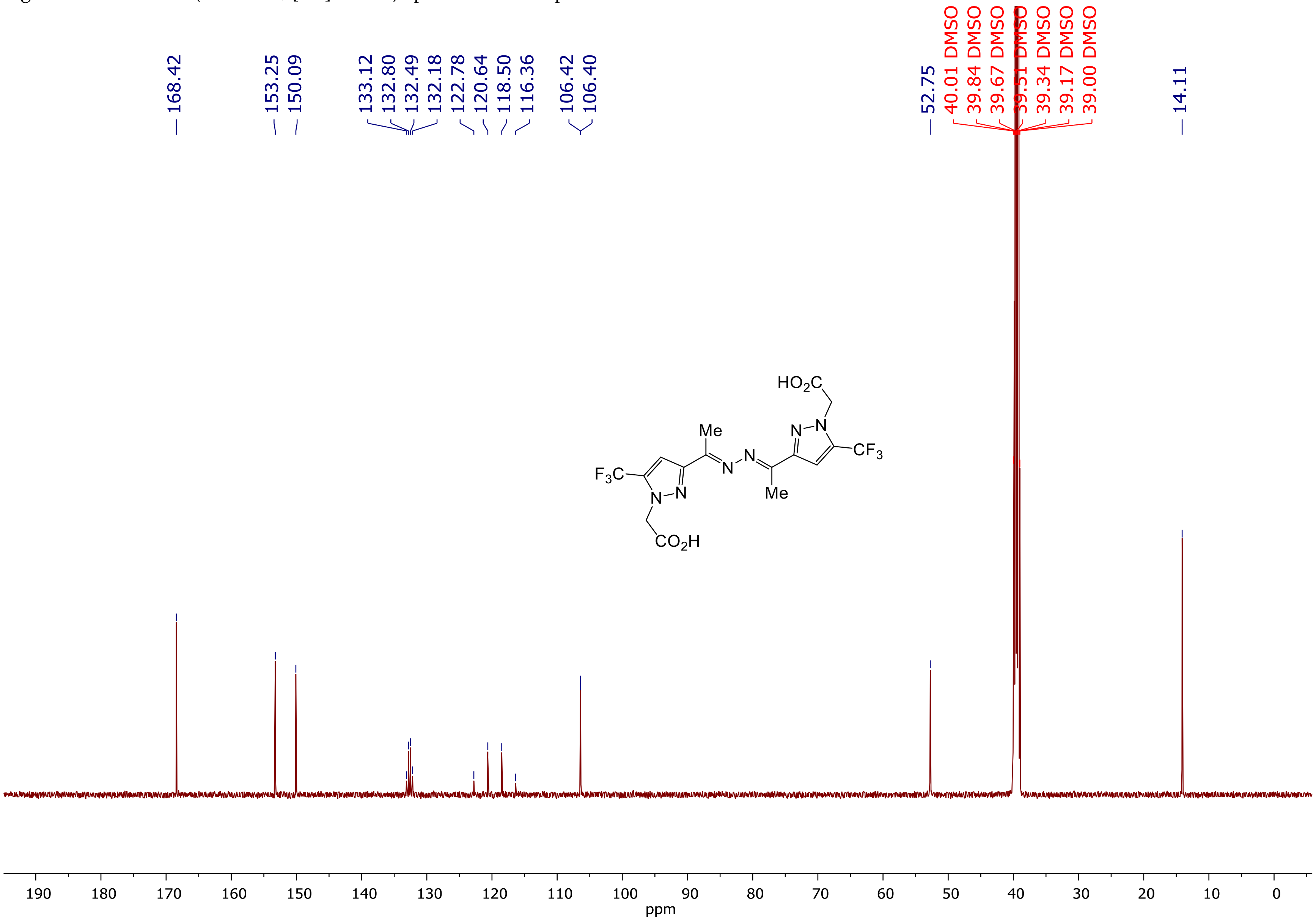

Figure S39. ^{19}F NMR (376 MHz, [D6]DMSO) spectrum of compound **24**

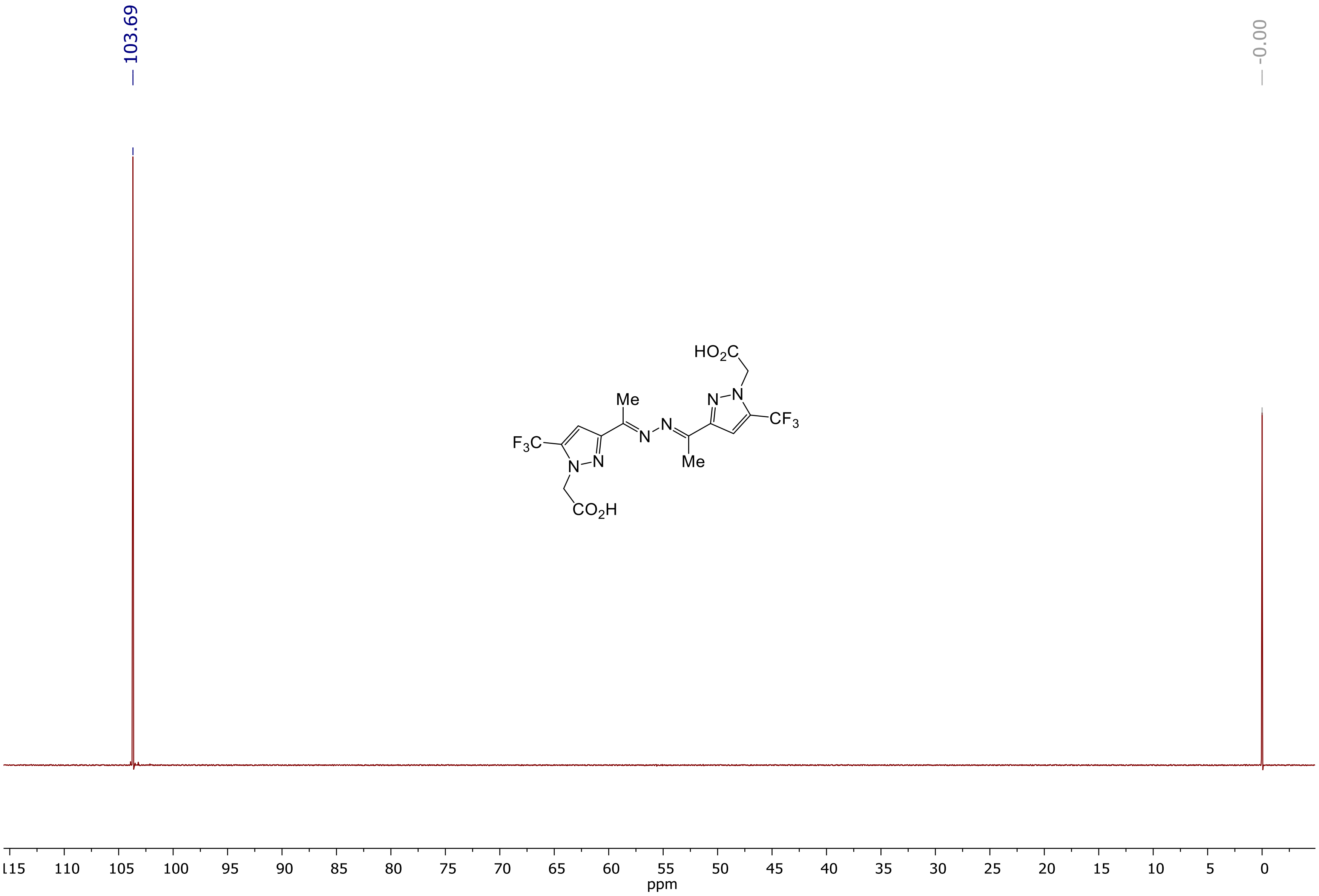

Figure S40. ^{1}H NMR (500 MHz, [D6]DMSO) spectrum of compound **25**

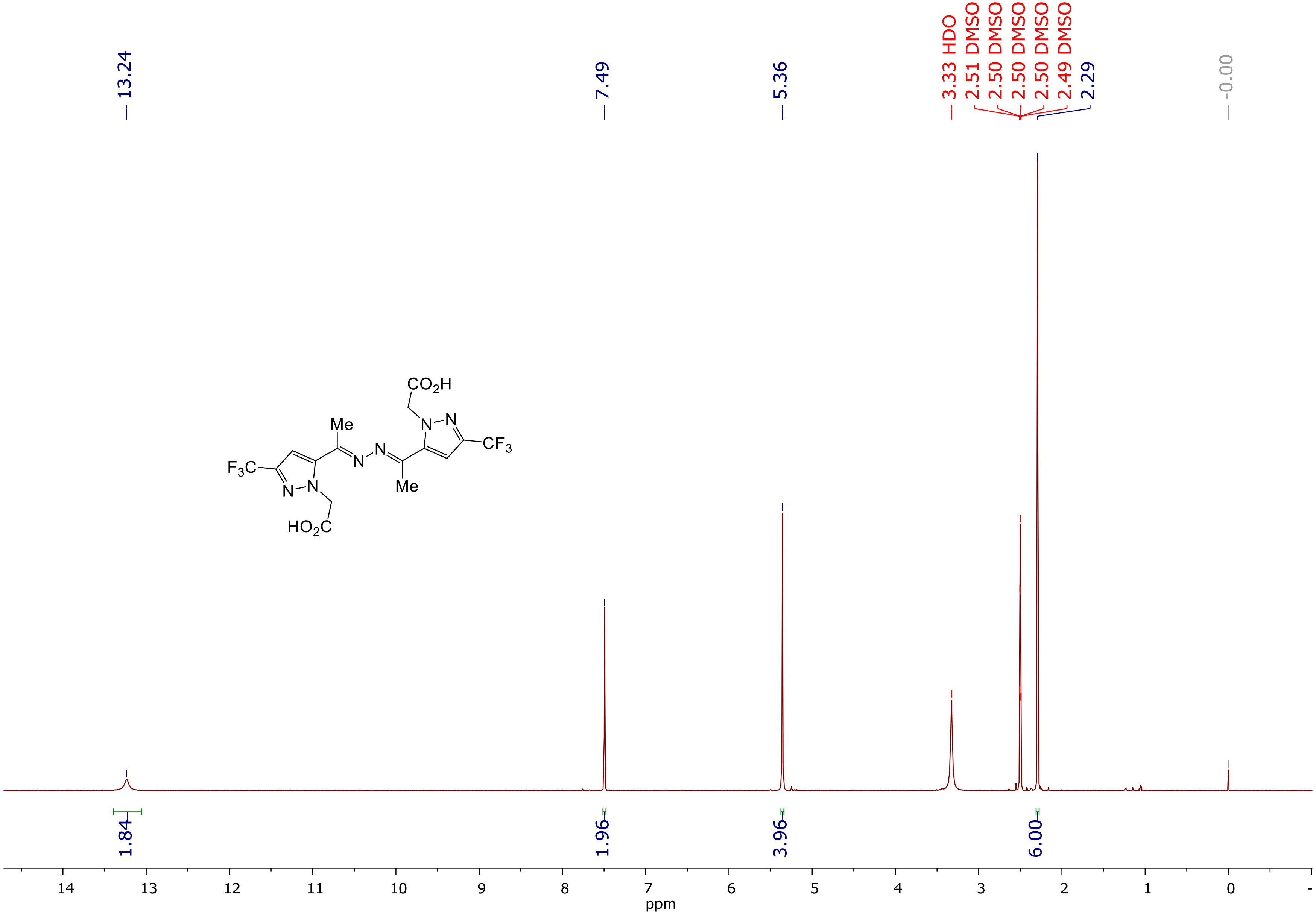

Figure S41. ^{13}C NMR (126 MHz, [D6]DMSO) spectrum of compound **25**

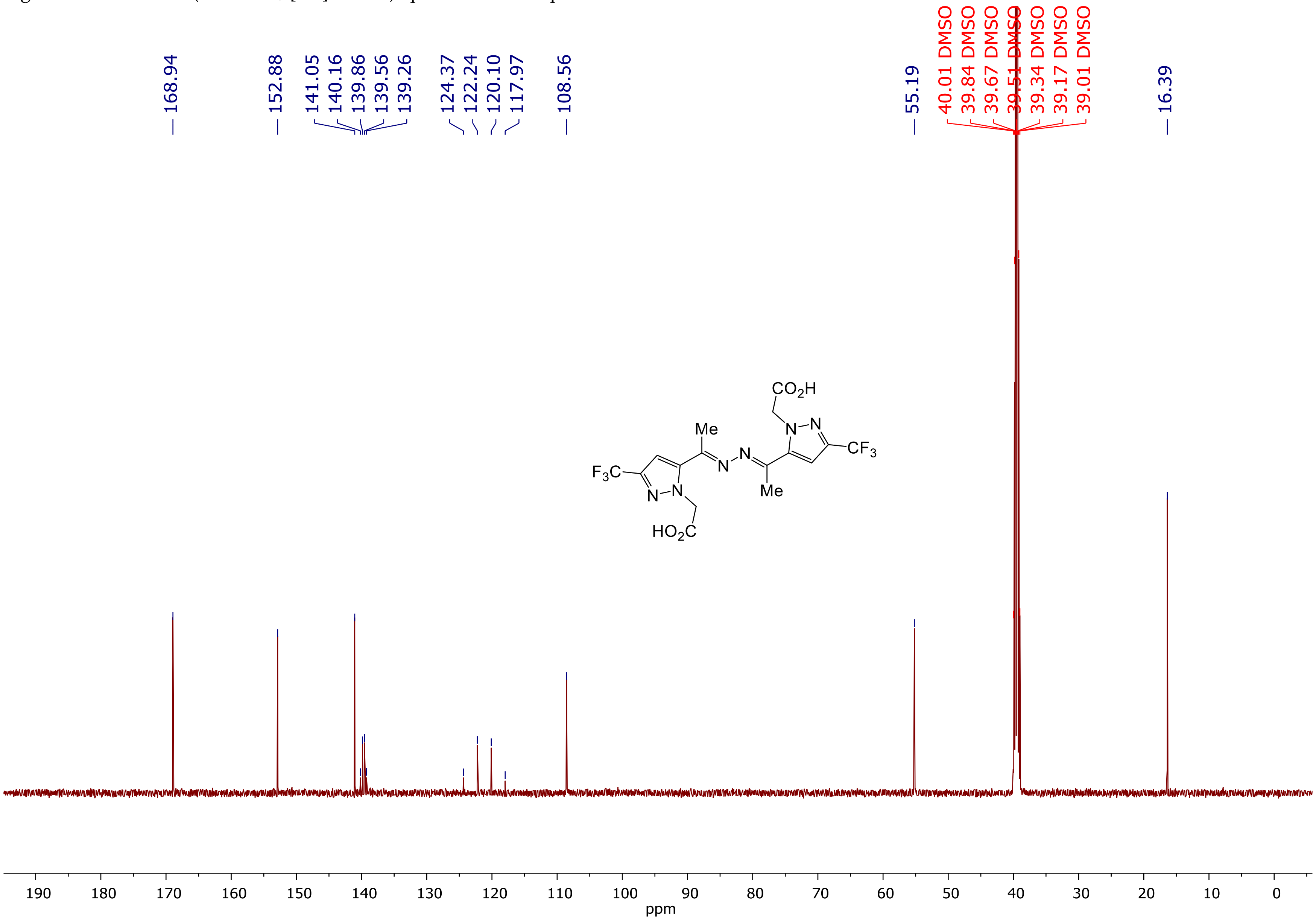

Figure S42. ^{19}F NMR (470 MHz, [D6]DMSO) spectrum of compound **25**

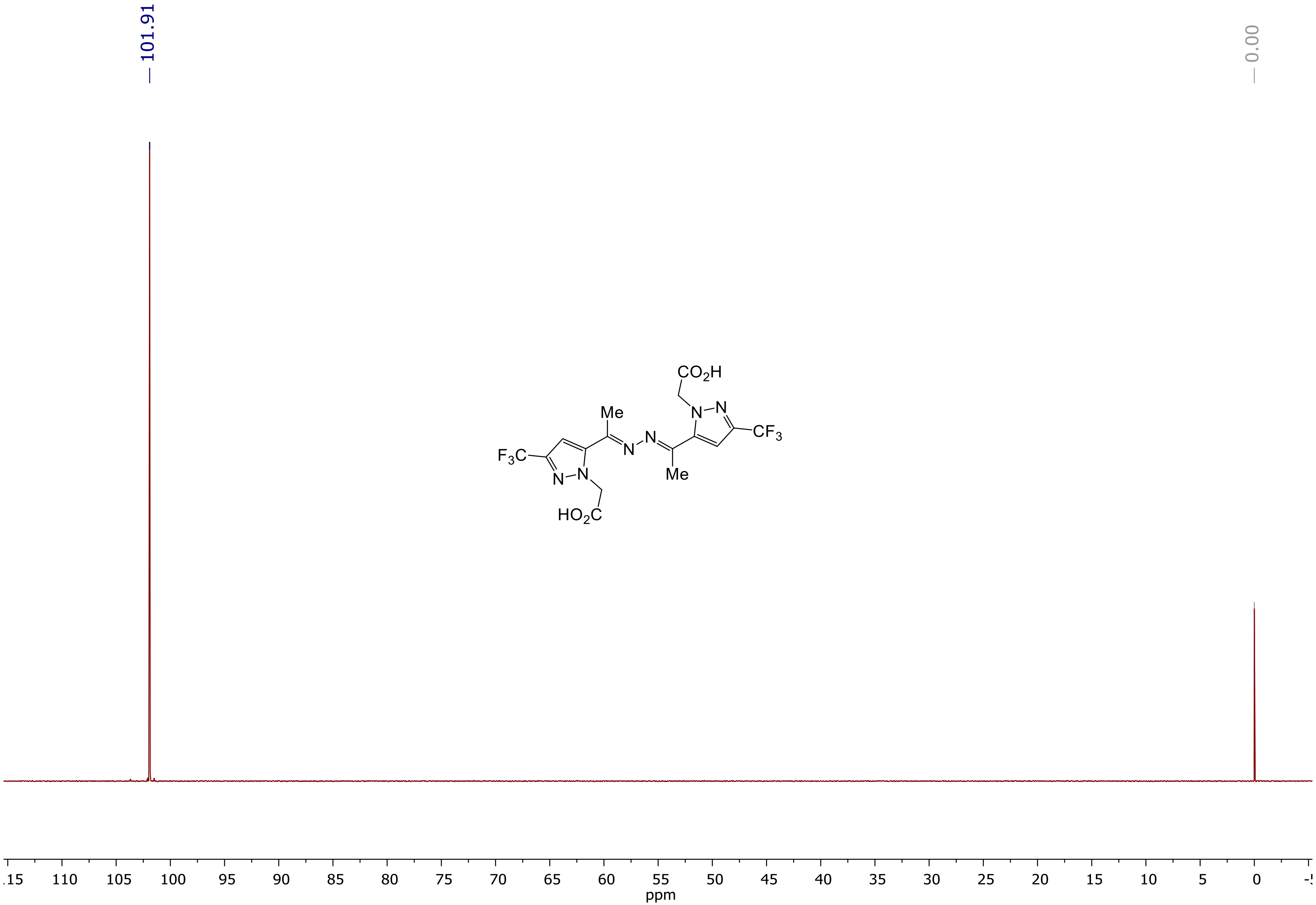

Figure S43. The structure of compound **15** showing the arrangement of substituents at the hydrazone fragment in two planes

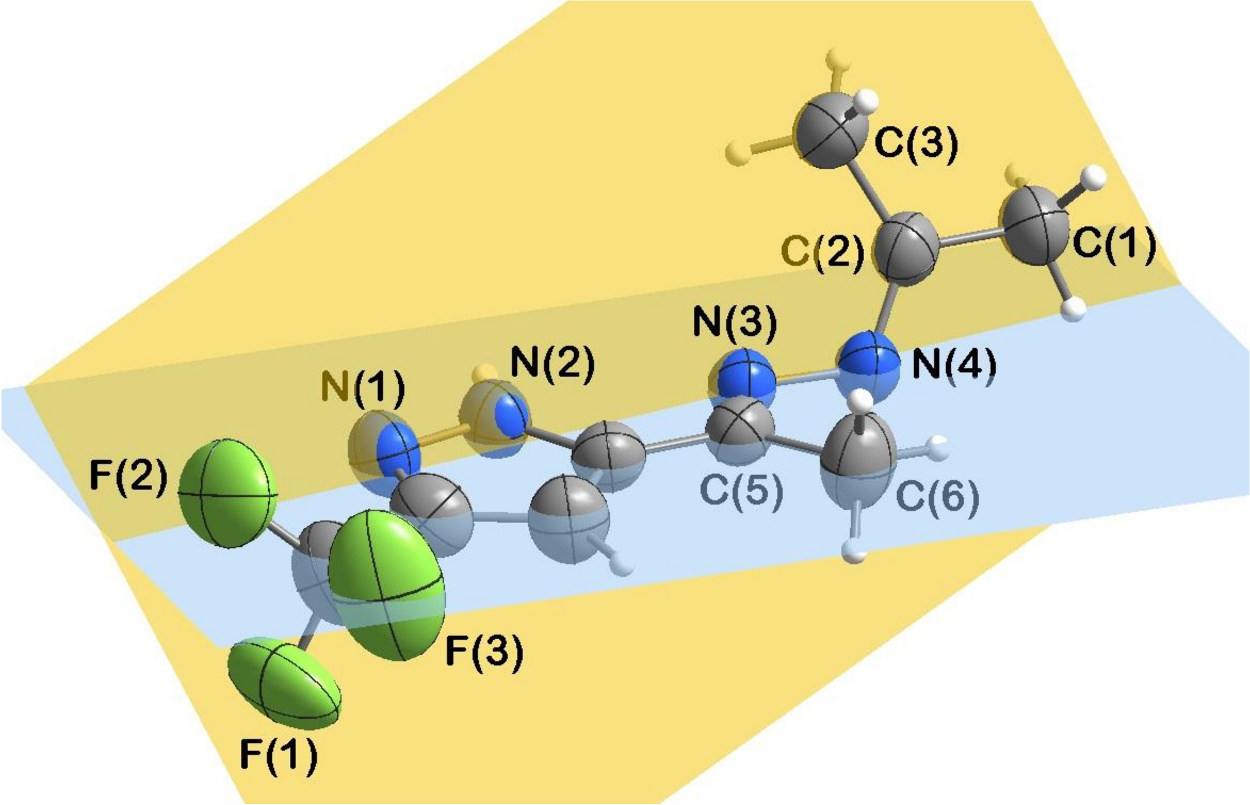

Figure S44. Fragment of the crystal packing of compound **4**

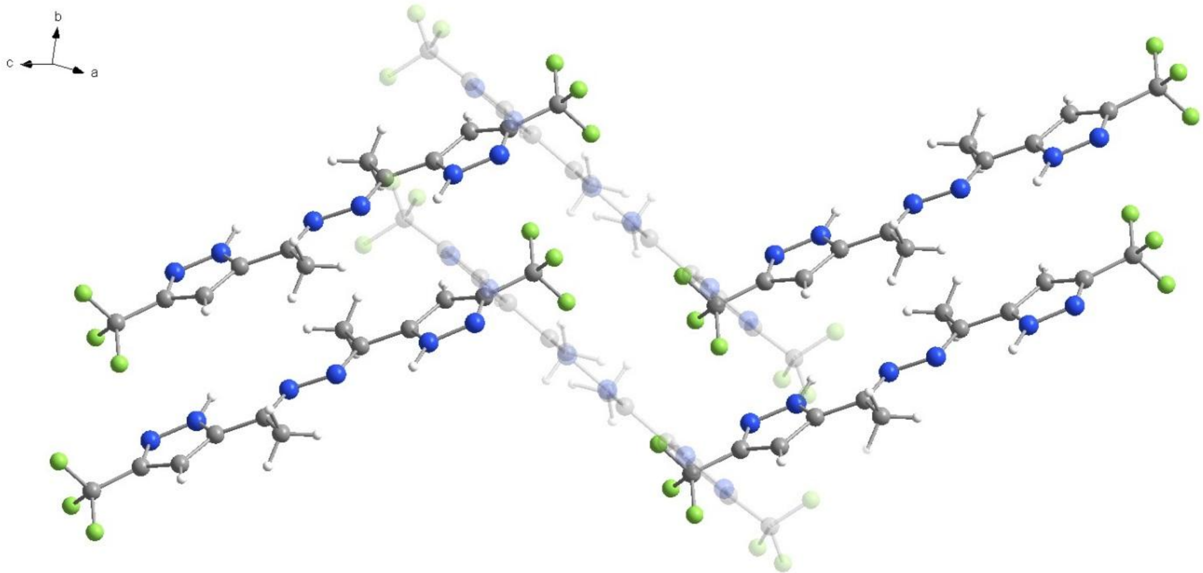

Figure S45. Fragment of the crystal packing of compound **13**

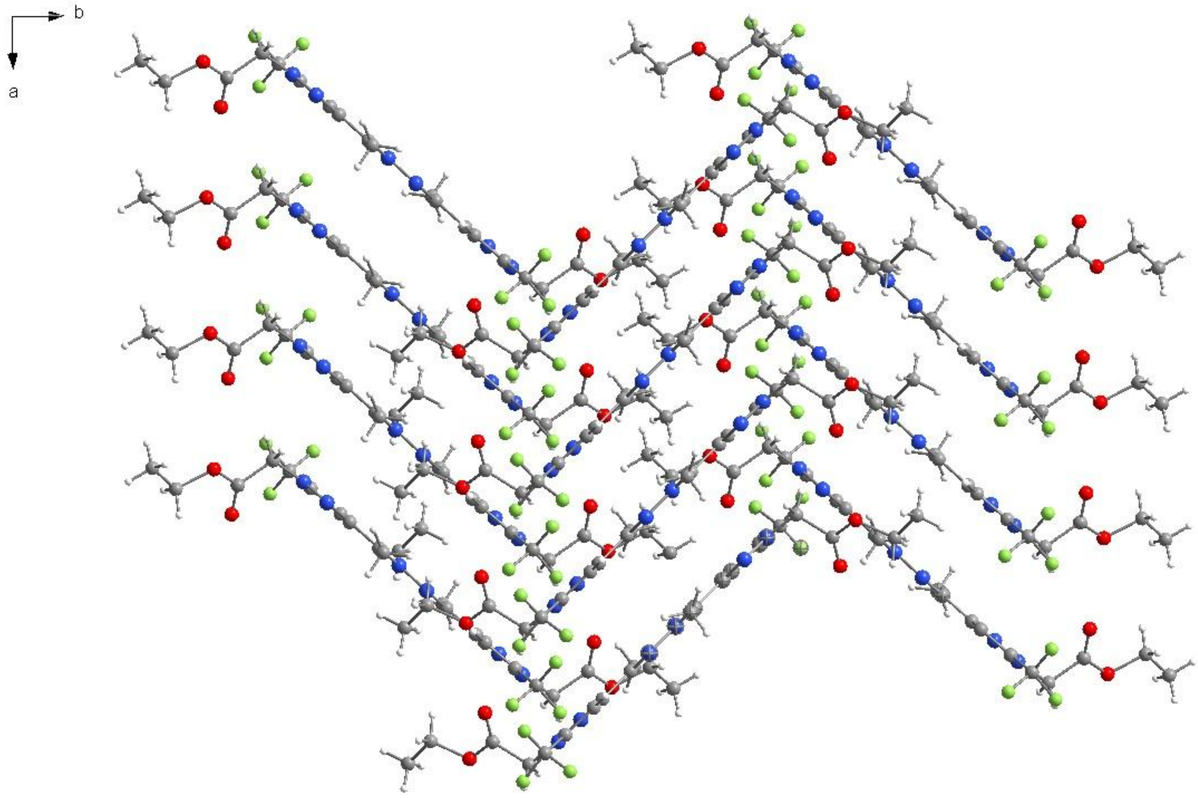

Figure S46. Fragment of the crystal packing of compound **14**

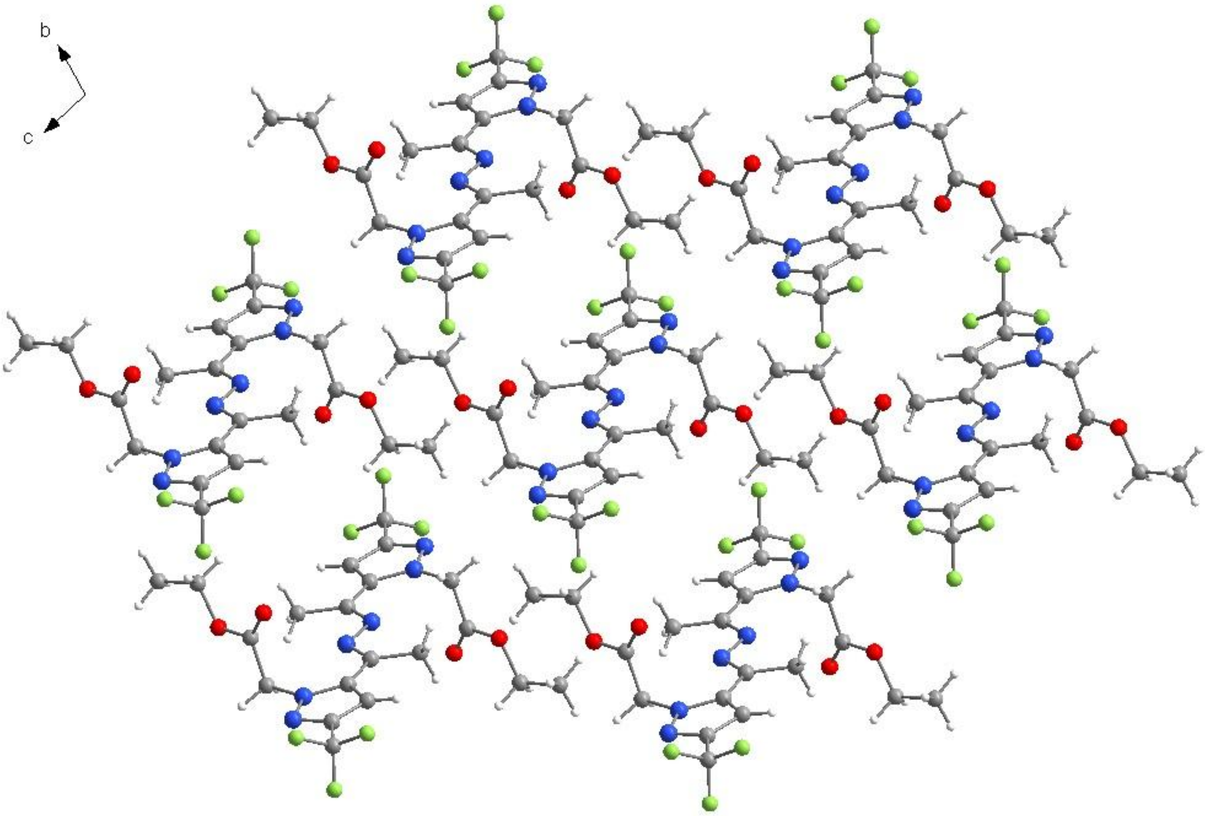

Figure S47. Fragment of the crystal packing of compound **15** showing the H(1)…N(3) intermolecular hydrogen bond equal to 2.202(27) Å

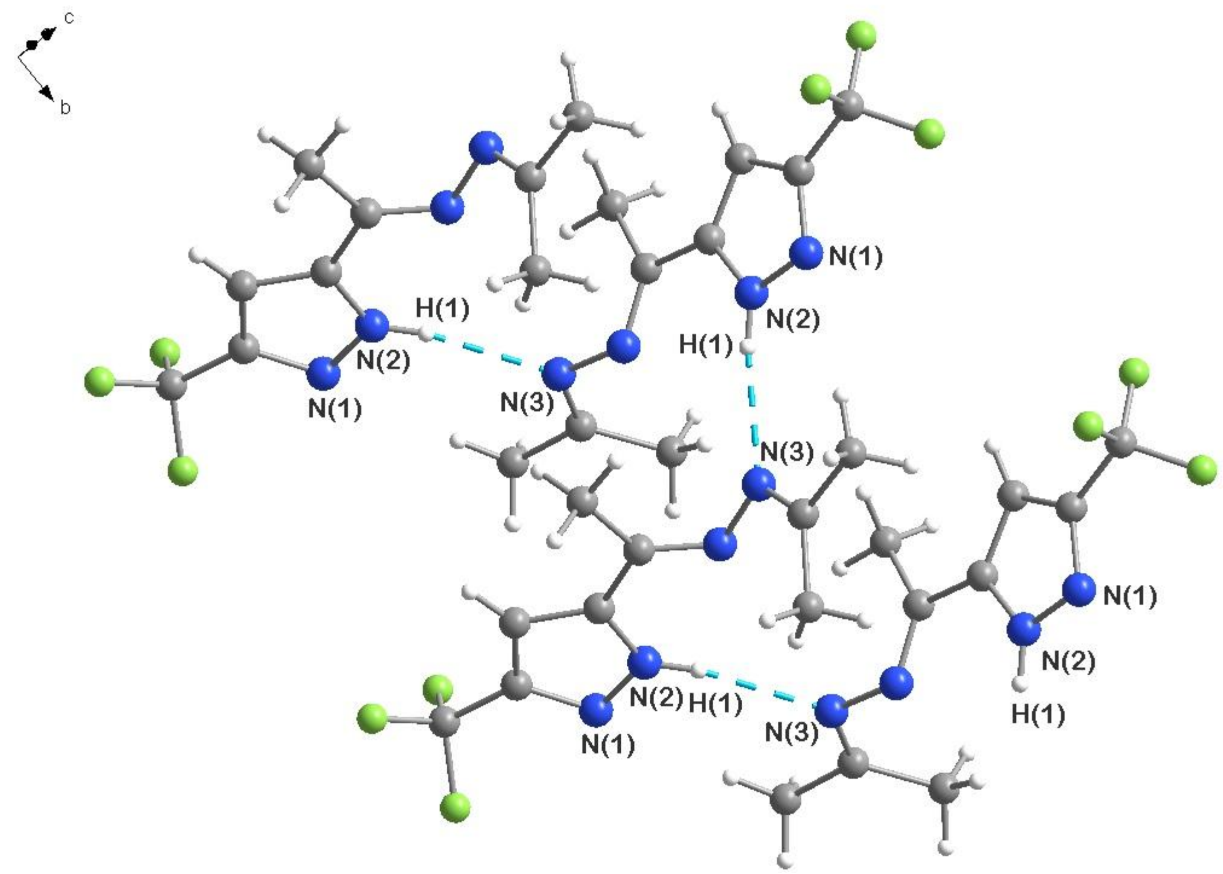

Figure S48. Fragment of the crystal packing of compound **16**

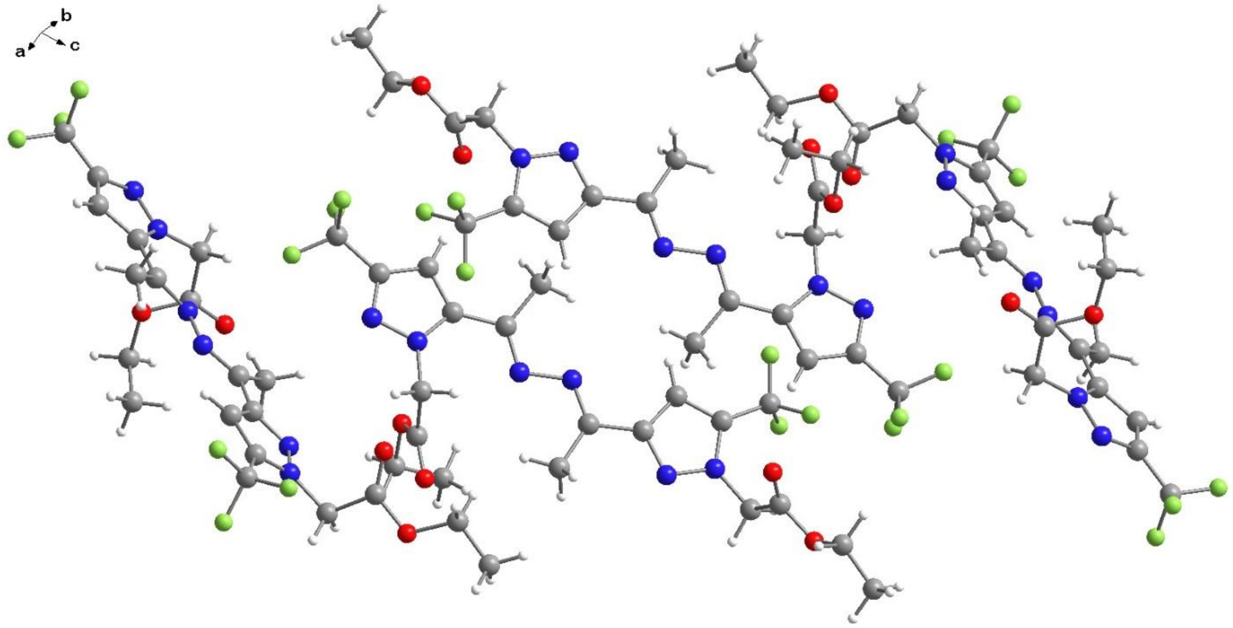

Figure S49. Fragment of the crystal packing of compound **25**

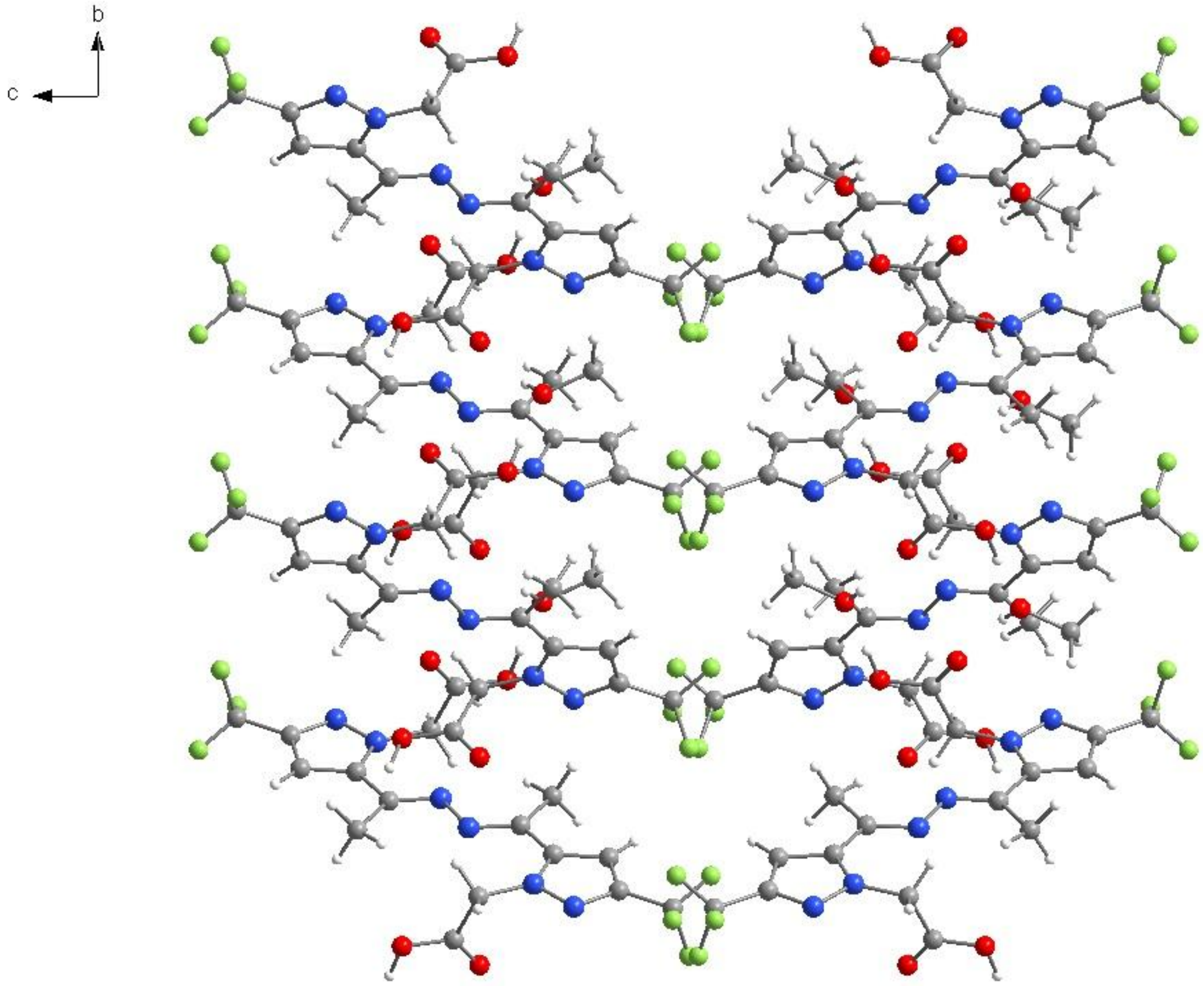

Table S1. Crystallographic parameters and structure refinement statistics for pyrazoles **4, 13** and **14**

Parameter	Compound		
	**4**	**13**	**14**
Molecular formula	$C_{12}H_{10}F_6N_6$	$C_{20}H_{22}F_6N_6O_4$	
M	352.24	524.43	
Temperature, K		295(2)	
Crystal system	Monoclinic	Monoclinic	Triclinic
Space group	$I2/a$	$P2_1/n$	$P\bar{1}$
Z	8	2	1
a, Å	16.5862(15)	4.7529(3)	7.8801(6)
b, Å	4.6384(4)	17.2477(7)	8.0915(7)
c, Å	21.197(2)	14.6423(11)	9.9714(7)
α, °	90	90	102.620(7)
β, °	113.788(12)	96.749(6)	100.052(7)
γ, °	90	90	95.363(7)
V, Å^3	1492.2(3)	1192.01(13)	605.15(8)
d_{calc}, g·cm^{-3}	1.568	1.461	1.439
θ_{min}-θ_{max}, deg	2.66-28.28	2.74-31.13	2.60-30.35
μ(MoKα), mm^{-1}	0.153	0.134	0.132
Reflection indices ranges	$-21 \leq h \leq 22$<br>$-6 \leq k \leq 6$<br>$-27 \leq l \leq 28$	$-6 \leq h \leq 4$<br>$-23 \leq k \leq 23$<br>$-13 \leq l \leq 21$	$-9 \leq h \leq 10$<br>$-10 \leq k \leq 8$<br>$-14 \leq l \leq 14$
Number of reflections	4893	5544	4370
Independent reflections	1813	3276	3174
Reflections with $I > 2\sigma(I)$	874	1575	1885
R_{int}	0.0381	0.0208	0.0244
$F(000)$	713	540	270
GOOF	1.012	1.000	1.040
R_1 / wR_2 for $I > 2\sigma(I)$	0.0622 / 0.1525	0.0618 / 0.1607	0.0640 / 0.1595
R_1 / wR_2 for all data	0.1384 / 0.2166	0.1372 / 0.2183	0.1064/ 0.2033
CCDC number	2488507	2488508	2488509

Table S1 (continued). Crystallographic parameters and structure refinement statistics for pyrazoles **15, 16** and **25**

Parameter	Compound		
	**15**	**16**	**25**
Molecular formula	$C_9H_{11}F_3N_4$	$C_{20}H_{22}F_6N_6O_4$	$C_{18}H_{22}F_6N_6O_4$*
M	232.22	524.43	532.41
Temperature, K		295(2)	
Crystal system	Monoclinic	Monoclinic	Orthorhombic
Space group	$P2_1/n$	$P2_1/c$	*Pbca*
Z	4	4	4
a, Å	9.7591(7)	12.4017(5)	10.0991(5)
b, Å	6.7668(5)	13.6975(4)	10.2412(6)
c, Å	18.0130(11)	15.5085(5)	23.5097(13)
α, °	90	90	90
β, °	93.643(6)	111.426(4)	90
γ, °	90	90	90
V, Å^3	1187.14(14)	2452.40(16)	2431.5(2)
d_{calc}, g·cm^{-3}	1.299	1.420	1.454
θ_{min}-θ_{max}, deg	2.44-28.28	2.31-30.99	2.66-30.86
μ(MoKα), mm^{-1}	0.116	0.131	0.139
Reflection indices ranges	$-5 \le h \le 13$<br>$-8 \le k \le 9$<br>$-20 \le l \le 24$	$-17 \le h \le 17$<br>$-19 \le k \le 15$<br>$-11 \le l \le 22$	$-7 \le h \le 14$<br>$-8 \le k \le 14$<br>$-17 \le l \le 31$
Number of reflections	4832	12046	6829
Independent reflections	2902	6640	3340
Reflections with $I > 2\sigma(I)$	1248	3245	1686
R_{int}	0.0272	0.0242	0.0275
$F(000)$	480	1080	1096
GOOF	0.979	1.026	1.024
R_1 / wR_2 for $I > 2\sigma(I)$	0.0645 / 0.1406	0.0650 / 0.1645	0.0634 / 0.1500
R_1 / wR_2 for all data	0.1554 / 0.2004	0.1428 / 0.2144	0.1312/ 0.1903
CCDC number	2488510	2488511	2488512

* Molecular formula of compound **25** was calculated including the two solvate methanol molecules.
